# Evaluation of Genetic Diversity and Development of a Core Collection of Wild Rice (*Oryza rufipogon* Griff.) Populations in China

**DOI:** 10.1371/journal.pone.0145990

**Published:** 2015-12-31

**Authors:** Wen Liu, Muhammad Qasim Shahid, Lin Bai, Zhenzhen Lu, Yuhong Chen, Lan Jiang, Mengyang Diao, Xiangdong Liu, Yonggen Lu

**Affiliations:** State Key Laboratory for Conservation and Utilization of Subtropical Agro-bioresources, South China Agricultural University, Guangzhou, China; Louisiana State University Agricultural Center, UNITED STATES

## Abstract

Common wild rice (*Oryza rufipogon* Griff.), the progenitor of Asian cultivated rice (*O*. *sativa* L.), is endangered due to habitat loss. The objectives of this research were to evaluate the genetic diversity of wild rice species in isolated populations and to develop a core collection of representative genotypes for *ex situ* conservation. We collected 885 wild rice accessions from eight geographically distinct regions and transplanted these accessions in a protected conservation garden over a period of almost two decades. We evaluated these accessions for 13 morphological or phenological traits and genotyped them for 36 DNA markers evenly distributed on the 12 chromosomes. The coefficient of variation of quantitative traits was 0.56 and ranged from 0.37 to 1.06. SSR markers detected 206 different alleles with an average of 6 alleles per locus. The mean polymorphism information content (PIC) was 0.64 in all populations, indicating that the marker loci have a high level of polymorphism and genetic diversity in all populations. Phylogenetic analyses based on morphological and molecular data revealed remarkable differences in the genetic diversity of common wild rice populations. The results showed that the Zengcheng, Gaozhou, and Suixi populations possess higher levels of genetic diversity, whereas the Huilai and Boluo populations have lower levels of genetic diversity than do the other populations. Based on their genetic distance, 130 accessions were selected as a core collection that retained over 90% of the alleles at the 36 marker loci. This genetically diverse core collection will be a useful resource for genomic studies of rice and for initiatives aimed at developing rice with improved agronomic traits.

## Introduction

*Oryza rufipogon* Griff., also known as common wild rice, is the progenitor of Asian cultivated rice (*Oryza sativa* L). Wild rice is widely distributed in the tropics and subtropics of Asia. In China, wild rice is distributed from 19 to 28°N in eight provinces, i.e., Hainan, Guangdong, Guangxi, Yunnan, Fujian, Taiwan, Hunan, and Jiangxi. Many populations of genetically diverse wild rice are present in these provinces [1–7]. It is essential that wild germplasm be characterized so that plant breeders can identify and conserve accessions with agronomically favorable traits. South China is considered to be the center of genetic diversity of wild rice. Wild rice plants with a high level of genetic diversity for adaptive traits (such as drought and disease resistance) were reported to exist in Guangdong and Hainan provinces [1,8–11].

The natural habitats of wild rice species have been shrinking due to urbanization [1,12]. Consequently, it is critical to evaluate the genetic diversity of available populations and develop a system to conserve the wild germplasm [13]. Wild rice is a vast reservoir of beneficial genes that can be used to breed rice with improved properties, such as increased resistance to biotic and abiotic stresses [1]. Therefore, evaluating the genetic diversity of wild rice will provide information about how best to use this germplasm in rice breeding. Molecular marker analysis is one of the most useful methods of investigating the genetic diversity of common wild rice [13–15]. However, one type of data may not be sufficient to accurately assess the genetic variability of a germplasm and to develop effective core collections that capture the maximum genetic diversity of a germplasm [15,16]. Therefore, both molecular marker and morphological data should be considered when constructing a core collection [17].

Morphological data are valuable for assessing and comparing diversity patterns within and among populations [18]. Molecular markers are increasingly being used to identify and evaluate genetic variation in wild rice populations [19,20]. Simple sequence repeats (SSRs) are powerful genetic markers due to their high levels of polymorphism, co-dominance of alleles, and wide distribution across the genome. SSR markers are commonly used to estimate the genetic diversity of wild rice populations [21].

Developing a core collection is an efficient approach for exploring, characterizing, and capturing the genetic diversity of large populations. Over 5,000,000 accessions of crop species are held in gene banks around the world [22]. A core collection is composed of a small proportion of representative genotypes that capture the largest amount of genetic diversity in the entire population [22,23]. The size of the core collection varies from 5 to 30% of the entire population. Core collections of germplasm have been developed for rice, *Glycine max* (soybean), *Arachis hypogaea* (peanut), and *Sesamum indicum* (sesame) crops [22,24–26]. Core collections facilitate the efficient exploration of novel genes in germplasm resources. Analyzing differences in genetic diversity of the core collection and entire population is a useful approach for testing the effectiveness of the core collection [17]. Genetic diversity parameters, such as total alleles, na, ne, h, I, PIC, and PCA, are frequently used to evaluate the genetic diversity of the core collection [17,27–29].

Previous collections of common wild rice were limited to individual populations from a few ecological regions [30–32]. However, this study focused on eight populations isolated from regions 19° to 28°N in China. In an effort to conserve and utilize this important progenitor of Asian cultivated rice, we: 1) evaluated the genetic diversity of these eight populations of wild rice based on quantitative and qualitative traits and DNA marker loci; 2) developed a core collection of wild germplasm to facilitate *ex situ* conservation; and 3) ensured that the core collection retained the genetic diversity of the entire population.

## Materials and Methods

### Ethics statement

All wild rice sites/populations are not open to the public, but researchers studying rice crops are allowed to visit the natural habitats of all wild rice germplasms. Because all the germplasms were collected by wild rice scientists, we did not need any specific permit to collect wild rice germplasms from those sites. The state encourages and supports scientific research of wild plants and the *in situ* and *ex situ* conservation of wild plants. All participants in this project have provided consent for publication of images (Figs A-C in S1 File).

### Wild rice populations

Wild rice plants from eight populations were collected from their natural habitats in Guangdong, Hainan, and Jiangxi provinces from 1996 to 2006. A maximum of one plant was collected per square meter of wild rice populations (S1 Table, Figs A and B in S1 File). These plants, distributed from 19 to 28°N represent eight populations (Table 1). There are six sub-populations of wild rice in Gaozhou; some sub-populations exist along riverbanks, while others grow in deep marshes. Dongxiang and Qionghai populations grow on hilly areas, whereas the Zengcheng population exists along the banks of the Yangtian River. The Fogang population grows on the banks of a mountain pool and is surrounded by wild plants, while the Huilai, Boluo, and Suixi populations are surrounded by mountains and wild rice is the dominant type of vegetation in these sites (S1 Table). The plants were numbered and transplanted (in bottomless pots of 16 cm in diameter and 16 cm in height; at a plant-to-plant distance of 25 cm and a row-to-row distance of 45 cm) in a protected garden at South China Agricultural University in Guangzhou (23°16'N, 113°8'E; Fig C in S1 File). Steel pipes covered with a net were used to separate the materials to ensure that the common wild rice accessions were under complete isolation. The growing conditions for the wild rice plants were similar to those of cultivated rice. However, we cut the plants manually in June and December every year, and stubbles (~20 cm) were left in the field for ratooning (i.e., growing from the stubbles of the previous season’s crop). After emergence from the leaf sheath, panicles were enclosed in paper bags to prevent out-crossing and seed shattering. A total of 885 accessions were planted in our wild rice garden and all these accessions were used in this study (Table 1).

**Table 1:** Geographical distribution and sampling information of wild rice populations.

Populations^a^	Geographical location	Number of accessions^b^	Core collection^c^	Sampling ratio (%)	Province
Qionghai (QH)	19°14′ N, 110°46′ E	70	14	20.0	Hainan
Suixi (SX)	21°38′ N, 110°24′E	83	10	12.1	Guangdong
Gaozhou (GZ)	21°95′ N, 110°83′E	188	25	13.3	Guangdong
Huilai (HL)	23°07′ N, 116°29′E	106	13	12.3	Guangdong
Zengcheng (ZC)	23°13′ N, 113°81′E	144	24	16.7	Guangdong
Boluo (BL)	23°18′ N, 114°28′E	84	12	14.3	Guangdong
Fogang (FG)	23°86′ N, 113°52′E	73	10	13.7	Guangdong
Dongxiang (DX)	28°14′ N, 116°35′E	137	22	16.1	Jiangxi

^a^Populations are represented by the names of the counties from where the wild rice plants were collected.

^b^Total number of accessions collected for this research.

^c^Number of accessions selected for core collection.

### Phenotypic identification of morphological traits

In this study, a total of ten quantitative traits (i.e., plant height, panicle length, awn length, spikelet length, spikelet width, spikelet length-to-width ratio, number of spikelets per panicle, number of filled spikelets per panicle, seed setting, and number of secondary branches), and three qualitative traits (i.e., leaf color, flowering rate, and growth habit) were evaluated at maturity following the protocol developed by the International Rice Research Institute (IRRI). These qualitative and quantitative traits, which have a large impact on rice yield and significantly affect the genetic diversity of the germplasm [14,20], were used to evaluate the genetic diversity of common wild rice according to previous studies [16,18,30,33]. All thirteen traits were evaluated in November 2013. The qualitative traits were scored as follows: leaf color was scored 1 for “dark green” or 2 for “light green”; flowering was scored as 1 for “absence of reproductive parts” or 2 for “presence of reproductive parts” [30]; and growth habit was scored 1 for “erect” (stalk is upright), 2 for “semi-erect” (part of the stalk is upright and part is inclined), 3 for “inclining” (the entire stalk is above the ground but not upright), and 4 for “creeping” (the stalk grows horizontally along the ground). Plant height was measured from the base to the top of the plant. Spikelet length and width were measured with a digital vernier caliper for a random sample of ten fully filled seeds. Number of spikelets per panicle, filled spikelets per panicle, and secondary branches were counted for five panicles on a plant. Seed set was calculated as the percentage of filled to the total number of spikelets on a plant [18,22].

### Marker selection and genotyping

Molecular markers with high levels of genetic diversity were selected from our previous study of genetic diversity in a core collection of cultivated rice [22]. From these markers, thirty-six SSR (simple sequence repeat) markers were selected and used to genotype the 885 accessions of wild rice. The selected markers were distributed on all 12 chromosomes of rice (S2 Table), with one marker on the short and two markers on the long arm of each chromosome [31,34]. Genomic DNA was extracted from leaves using a modified SDS method [35]. Polymerase chain reaction (PCR) was used to amplify specific genomic DNA sequences in a 20 μl volume containing 30 ng template, 0.15 μmol/L primer pairs, 1.0 μl dNTPs (2.0 mmol/l each), one unit Taq polymerase, and 1×PCR buffer (50 mmol/L KCl, 10 mmol/L Tris-HCl pH 8.3, 1.5 mmol/L MgCl_2_, 0.01% glutin). The PCR profile was 94°C for 5 minutes followed by 30 cycles of 94°C for 45 s, 55°C for 45 s, and 72°C for 45 s, and a final extension at 72°C for 5 minutes. PCR products were separated by electrophoresis on a 6% polyacrylamide denaturing gel and displayed by silver nitrate staining [36]. A DNA ladder of 100 to 1000 bp was used to estimate the size of the PCR products. Marker alleles on the gel were imaged using the BIO Imaging System and Genetools software (SynGene). Each image was read twice to reduce errors [34].

### Data analysis and genetic parameter estimation

Phenotypic and genotypic data were used to estimate genetic diversity and construct the core collection. Dendrograms of SSR markers and morphological traits were analyzed by NTSYSpc version 2.10 and SPSS17.0 software, respectively [37,38]. Box plots were used to determine the frequency distribution of quantitative traits among populations [39,40]. Box plots showing the distribution of ten quantitative traits from eight populations were developed using Sigmaplot software version 12.5. To assess whether the relationship between the qualitative traits (i.e., leaf color, flowering rate, and growth habit) and latitude was linear, graphs were created using Sigmaplot software version 12.5. A Mantel test was conducted using NTSYSpc version 2.10 to test for correlation between percentage of accessions exhibiting a particular qualitative trait (for instance, dark green leaf color, growth habit and flowering) and geographical locations.

Packing diagrams illustrate the genetic diversity in populations detected using SSR loci [41], and were created in EXCEL. The number of alleles at each locus (ne; effective number of alleles) is also a commonly used parameter to evaluate the genetic diversity of populations. Polymorphic alleles, total alleles, percentage of polymorphic bands (PPB), average number of alleles (na), and Shannon's information index (I) were estimated using POPGENE [42]. PPB, na, ne, and I were used to analyze the molecular data, and these parameters reflect the population genetic diversity and support the conservation strategies. POPGENE was also used to estimate the total genetic diversity (Ht) from all the wild rice populations and the mean genetic diversity within wild rice populations (Hs). The coefficient of gene differentiation (Gst; estimated by partitioning of the total genetic diversity residing among populations) and gene flow (Nm) were calculated using POPGENE. Shannon's information index (I) was calculated using the equation: I = −$\sum_{i=1}^{S}$ *P*_*i*_ln*P*_*i*_ (*P*_*i*_ = n/N, where n represents number of accessions in a population and N is the total number of accessions in all populations; ln is the natural log; Σ is the sum of calculations; and *S* is the number of populations), and accounts for the abundance or richness of a species [13,16]. Nei's gene diversity (h) was calculated using the equation: h = 2n (1−Σ*Pi* ^2^) /(2n - 1) (where n is the number of populations sampled and *pi* is the allele frequency at a given locus) to estimate the average genetic diversity per locus within an individual population [43]. The polymorphism information content (PIC = 1−$\sum_{i=1}^{l}$ *P* ^*2*^_*i*_−$\sum_{i=1}^{l-1}$ $\sum_{l=i+1}^{l}$2*P* ^2^_*i*_ *P* ^2^_*j*_, *P*_*i*_ and *P*_*j*_ are the population frequency of the *i* ^th^ and *j* ^th^ allele) was estimated using PICcale software (Yellow Sea Fisheries Research Institute, Chinese Academy of Fishery Sciences, 2007). PIC is a measure of a marker’s ability to detect polymorphisms in a population, based on the number of alleles detected and their frequency distribution; hence, it provides an estimate of the discriminating power of a marker [44].

To assess variation in the morphological traits and molecular data, the analysis of variance (ANOVA) and analysis of molecular variance (AMOVA) were performed using SPSS17.0 [37] and ARLEQUIN, respectively [45]. The percentage of variation among and within populations was estimated using AMOVA. Nei's genetic distance and genetic identity are useful indexes to determine similarities or differences among populations and were estimated using POPGENE. The morphological and molecular data were combined to analyze genetic diversity using SPSS17.0. Principal component analysis (PCA) implemented in SPSS17.0 was used to analyze the relationships between populations [37].

### Development of a core collection of wild rice

Three strategies were used to select wild rice accessions for long-term conservation [38]. (1) Strategy 1 was based on the magnitude of genetic distance, which is an estimate of the genetic divergence between populations within a species, determined using two individual phylogenetic trees (constructed using phenotypic and genotypic data), and accessions with high genetic distance were selected. (2) In Strategy 2, accessions were grouped by hierarchical cluster analysis based on genetic distance. (3) In Strategy 3, the core collection was selected from different groups of clusters, and the sampling proportion of the core collection was 10–20% of a population. A core collection was developed using QGAStation 2.0 (http://ibi.zju.edu.cn/software/qga/) and Venn analysis (http://bioinfogp.cnb.csic.es/tools/venny/) was performed to compare this core collection with the one based on genetic distance.

Furthermore, genetic diversity parameters, including number of alleles, na, ne, h, I, and PIC, were used to estimate the representativeness of the core collections. These genetic diversity parameters were compared between each population and its core collection using Student's *t*-test (*X* ^*2*^). If the p-value was greater than 0.05, then the difference between the core and entire population was considered to be non-significant. PCA was used to identify the major sources of variation between the accessions selected as the core collection and the eight populations. If the core sets were distributed uniformly in the PCA distribution graph, the core collections were considered to maintain a high level of genetic diversity and to be representative of the whole germplasm.

## Results

### Genetic diversity in morphological traits

**Fig 1:**
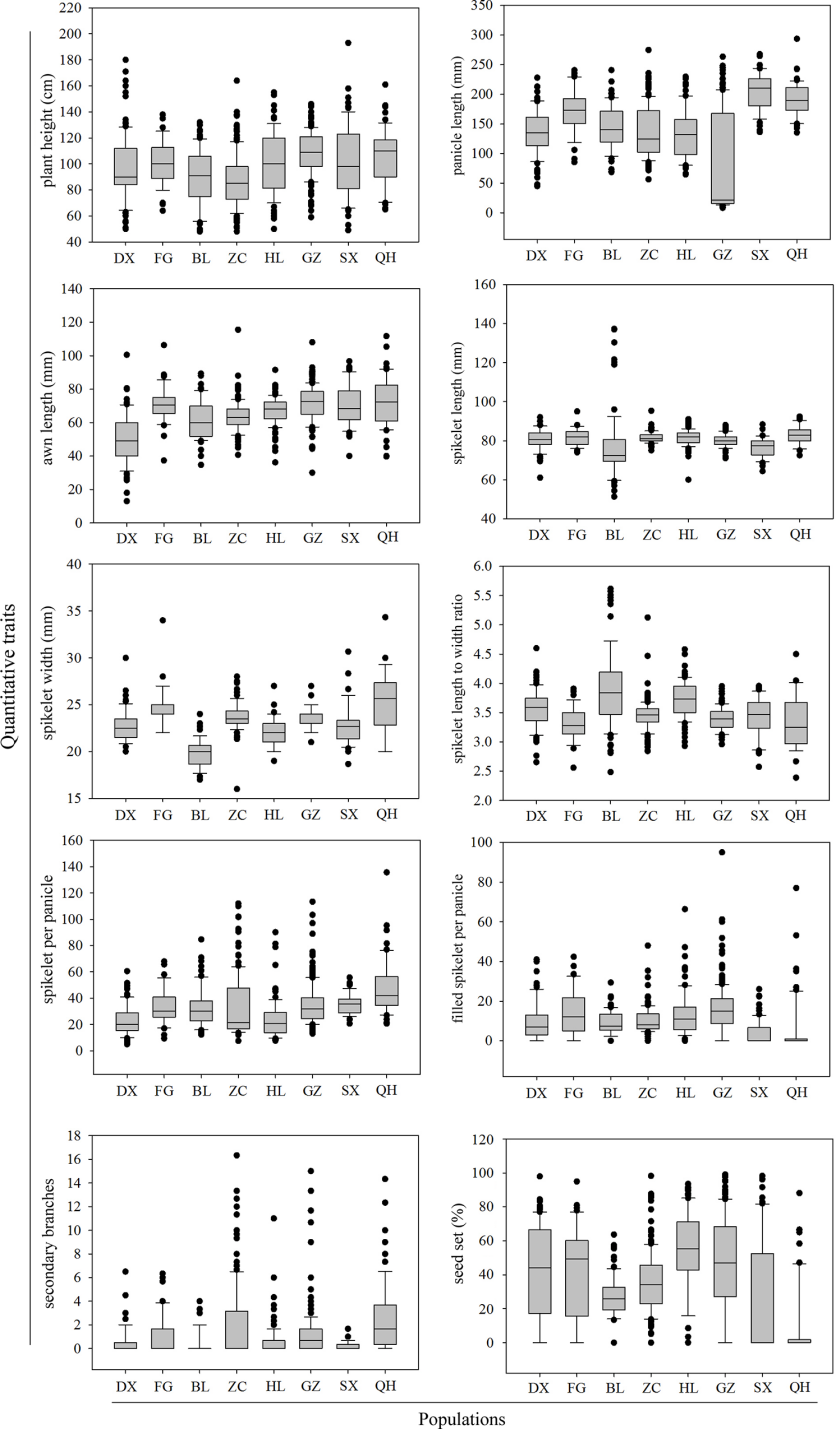
Box plots showing the distributions of ten quantitative traits from eight populations of wild rice. The upper, median, and lower quartiles of gray boxes represent the 75^th^, 50^th^, and 25^th^ percentiles of the populations, respectively. The vertical lines represent the variation of the population. Dots represent outliers. Dongxiang (DX) from Jiangxi province, Qionghai (QH) from Hainan province, and Fogang (FG), Boluo (BL), Zengcheng (ZC), Huilai (HL), Gaozhou (GZ), and Suixi (SX) from Guangdong province.

Box plots in Fig 1 show the frequency distribution for each of ten quantitative traits in the eight populations of wild rice collected (Table 1). For the plant height trait, the Dongxiang (DX) and Suixi (SX) populations displayed the largest and the Fogang (FG) population exhibited the smallest variation. There was a great variation in panicle length between populations, and the SX and Qionghai (QH) populations were found to have longer panicles than the other populations. The DX population had the shortest awns, whereas the awn length of other populations did not differ. The Zengcheng (ZC), Gaozhou (GZ), and QH populations had more spikelets per panicle and more secondary branches than did the other populations, which suggests that these three populations are close relatives. The Boluo (BL) population had longer spikelets and larger spikelet length-to-width ratios than did the other populations. The spikelet width was greatest for the QH population, while the BL population had the lowest spikelet width. The number of filled spikelets per panicle and the percentage of seed setting were higher in GZ and Huilai (HL) than in the other populations. These results demonstrate that substantial differences exist between the eight common wild rice populations derived from different environments. Quantitative trait diversity assessment showed significant variation between the eight populations for the ten quantitative traits assessed. Plant height, panicle length, and awn length ranged from 87–105 cm, 83–204 mm, and 50–73 mm, respectively. For yield-related traits, the QH population had the most spikelets per panicle (48) and the most secondary branches (3), but the fewest filled spikelets per panicle (7). The HL population had the highest seed setting percentage (60.3%). Spikelet length, spikelet width, and spikelet length-to-width ratio also differed among the wild rice populations.

The coefficient of variation (CV) is a useful statistic for comparing the variability of a morphological trait among populations. The highest variability among populations was observed for the number of secondary branches (CV = 8.00), while seed setting percentage (CV = 0.03) exhibited the lowest variability. The BL population has the highest mean CV for the ten quantitative traits, due to the particularly high CV of the number of secondary branches. Although the CV of awn length and number of spikelets per panicle was higher than that of the number of secondary branches in the QH population, a comparative analysis revealed that the CV of the number of secondary branches was significantly higher than that of the seven remaining traits (Table 2). We performed ANOVA (analysis of variance) to examine the significance of the variation in the ten quantitative traits among populations, and found that the difference in the ten traits among populations were significant (p<0.05; S3 Table).

**Table 2:** Summary of statistics for ten quantitative traits in wild rice populations.

Populations	Plant height (cm)	Panicle length (mm)	Awn length (mm)	Spikelet length (mm/ten spikelets)	Spikelet width (mm/ten spikelets)	Spikelet length-to-width ratio	Number of filled spikelets per panicle	Number of spikelets per panicle	Seed setting (%)	Number of secondary branches	Mean coefficient of variance
Dongxiang (DX)	95.3 (0.30)	135.2 (0.29)	50.3 (0.29)	81.0 (0.07)	23.0 (0.08)	3.6 (0.09)	12.2 (0.76)	23.0 (0.51)	53.0 (0.42)	0.6 (2.18)	0.50 (0.63)
Boluo (BL)	88.5 (0.27)	144.7 (0.26)	61.3 (0.45)	77.6 (0.07)	19.8 (0.19)	3.9 (0.19)	9.7 (0.38)	32.8 (0.55)	29.5 (0.22)	0.0 (8.00)	1.06 (2.44)
Zengcheng (ZC)	86.9 (0.24)	136.2 (0.32)	63.6 (0.70)	81.5 (0.06)	23.7 (0.08)	3.5 (0.15)	10.2 (0.50)	33.2 (0.63)	30.7 (0.03)	2.1 (1.61)	0.43 (0.48)
Gaozhou (GZ)	108.2 (0.15)	82.7(0.08)	70.4 (0.46)	80.1 (0.05)	23.7 (0.06)	3.4 (0.18)	17.9 (0.49)	35.3 (0.65)	50.7 (0.04)	1.4 (1.63)	0.47 (0.52)
Huilai (HL)	99.7 (0.25)	132.6 (0.31)	66.8 (0.62)	81.5 (0.07)	22.1 (0.19)	3.6 (0.13)	14.4 (0.36)	23.9 (0.76)	60.3 (0.05)	0.7 (2.21)	0.50 (0.65)
Fogang (FG)	101.4 (0.17)	172.4 (0.22)	70.6 (0.41)	81.8 (0.08)	24.9 (0.08)	3.3 (0.16)	16.9 (0.54)	33.6 (0.62)	50.3 (0.05)	1.4 (1.35)	0.37 (0.40)
Suixi (SX)	98.4 (0.32)	204.4 (0.16)	70.0 (0.22)	76.7 (0.10)	22.8 (0.10)	3.4 (0.18)	7.6 (1.45)	35.2 (0.90)	21.6 (0.07)	0.3 (1.64)	0.51 (0.60)
Qionghai (QH)	105.2 (0.21)	191.5 (0.15)	72.6 (2.16)	82.8 (0.14)	25.2 (0.14)	3.3 (0.21)	7.1 (0.44)	48.2 (2.08)	14.7 (0.06)	3.0 (1.02)	0.66 (0.82)
Mean	98.0(6.98)	150.0 (36.15)	65.7 (6.84)	80.4 (2.00)	23.2 (1.60)	3.5 (0.19)	12.6 (3.82)	32.6(7.28)	38.9 (0.18)	1.2 (0.94)	0.56 (0.82)
Correlation coefficients	0.36	0.41	0.75	0.02	0.31	0.42	0.04	0.44	0.17	0.19	0.06

The values in parentheses are coefficients of variation, CV (i.e., the standard deviation divided by the sample mean).

**Fig 2:**
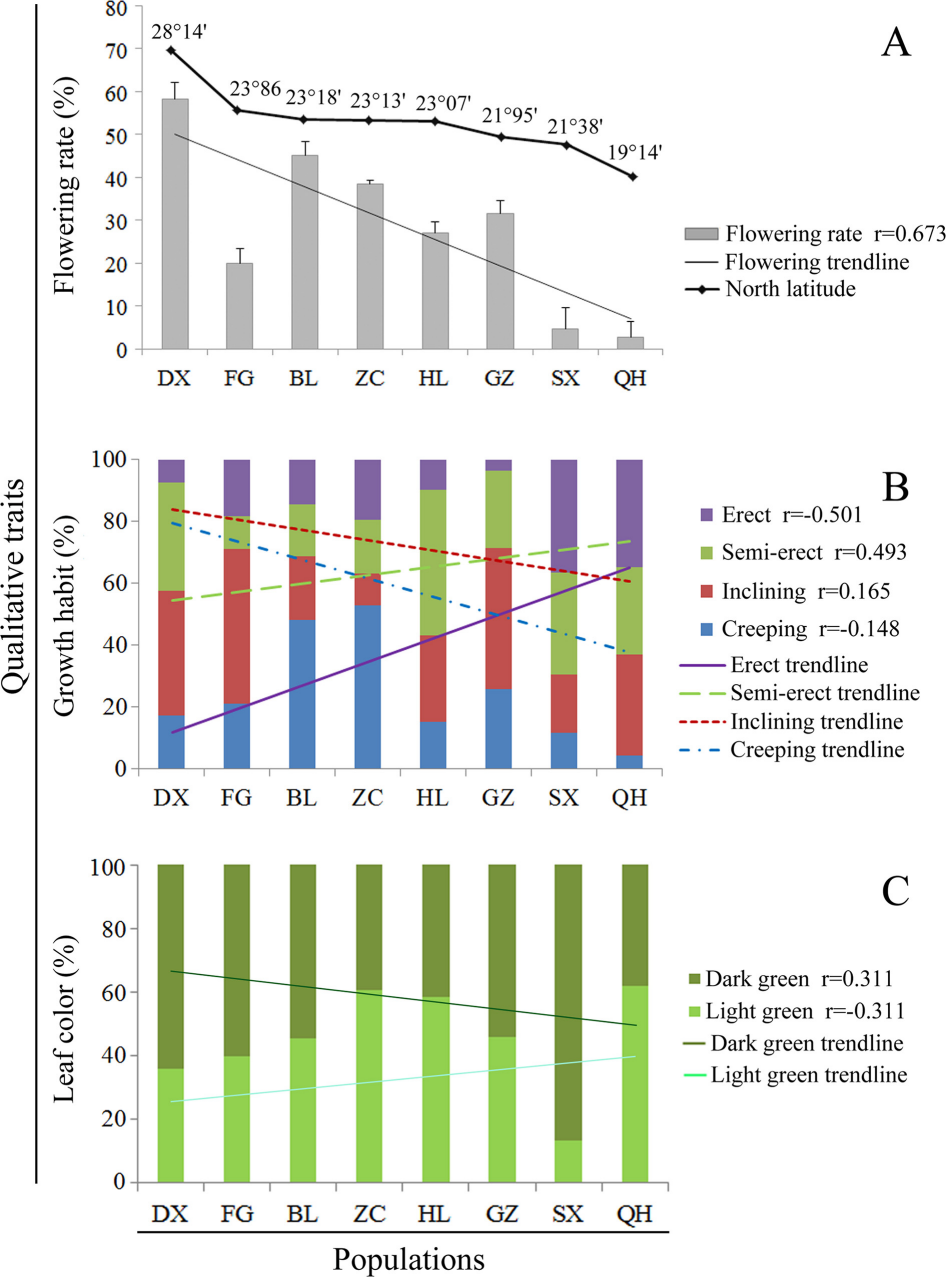
Correlation between quantitative traits and geographical locations of origin. Flowering rate, leaf color, and growth habit of entire populations were compared with different geographical locations/sites. Bars indicate SD (standard deviation) (Fig 2A), r values indicate correlation coefficients between qualitative traits and geographical locations of origin. Erect means the stalk is upright without physical support; semi-erect means some part of the stalk is upright and some is inclined; inclining means the entire stalk is above the ground but not upright; and creeping means the stalk grows horizontally along the ground. See Table 1 for details of populations.

Correlations between latitude and particular traits may represent genetic differentiation between populations in response to environmental characteristics [46]. We determined the correlation between the means of three qualitative traits (leaf color, flowering rate, and growth habit) and the latitudes from which the populations originated (Fig 2). Populations arising from further north latitudes had higher flowering rates when cultivated *ex situ* in Guangzhou. The Mantel test showed a significant correlation (r = 0.673, p = 0.005) between flowering rate and distance north of the equator (Fig 2A). The erect and semi-erect types of growth were more abundant in the SX and QH populations, while the creeping growth habit was more common in ZC and BL than in the other populations (Fig 2B). These results revealed that erect and semi-erect types of growth were more common in the low north latitudes. Except for the SX population (which originated in the lower north latitudes and had the highest proportion of accessions with dark green leaves), populations further north of the equator tended to have darker green leaves than those closer to the equator (Fig 2C). Moreover, correlation coefficient analysis between quantitative traits and latitude showed that awn length had the strongest (0.75) and spikelet length had the weakest (0.02) relationship with latitude (Table 2). These results suggest that genetic differentiation in the eight populations might have arisen due to geographic isolation.

### Genetic diversity at marker loci

**Fig 3:**
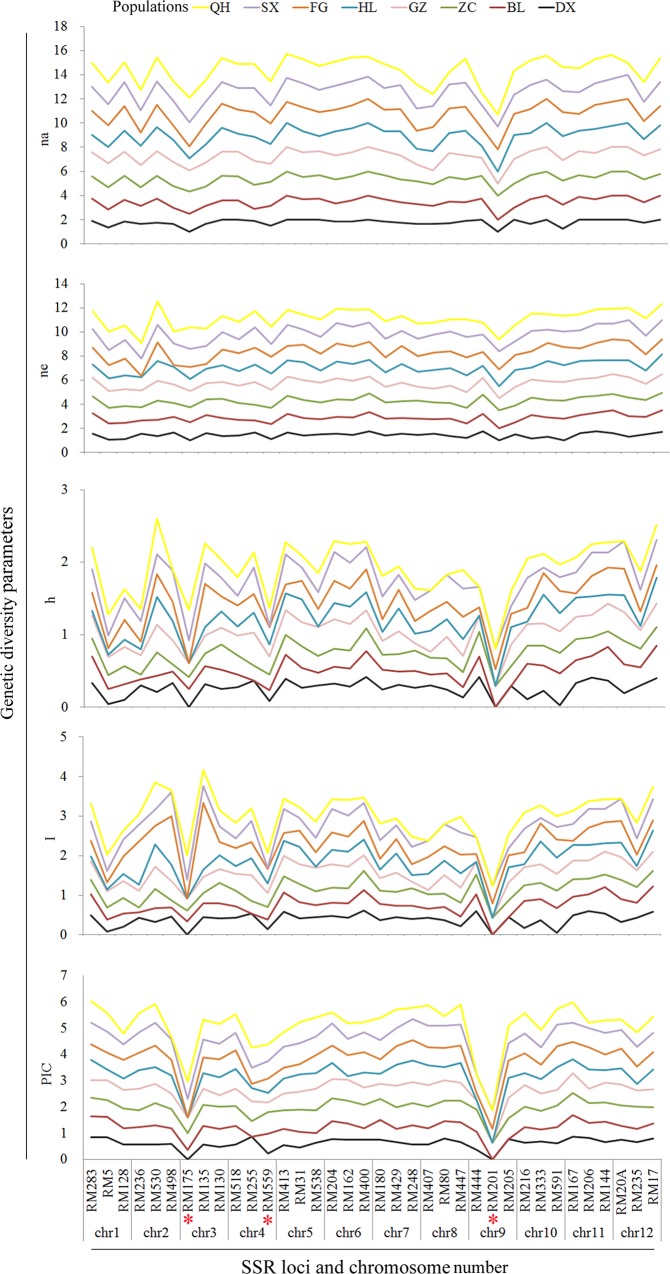
Packing diagram of the genetic diversity parameters at each locus of rice’s twelve chromosomes. na, ne, h, I, and PIC represent the number of alleles, effective number of alleles (number of alleles at each locus), Nei's gene diversity, Shannon's information index, and polymorphism information content, respectively. * indicates the conserved loci. See Table 1 for details of populations.

We next genotyped 885 accessions from eight populations for 36 SSR markers (S4 Table). Packing diagrams of na, ne, h, I, and PIC genetic parameters showed three conserved positions or loci in common wild rice populations. These three loci, including RM175, RM559, and RM201, were present on chromosome 3, 4, and 9, respectively (Fig 3).

**Fig 4:**
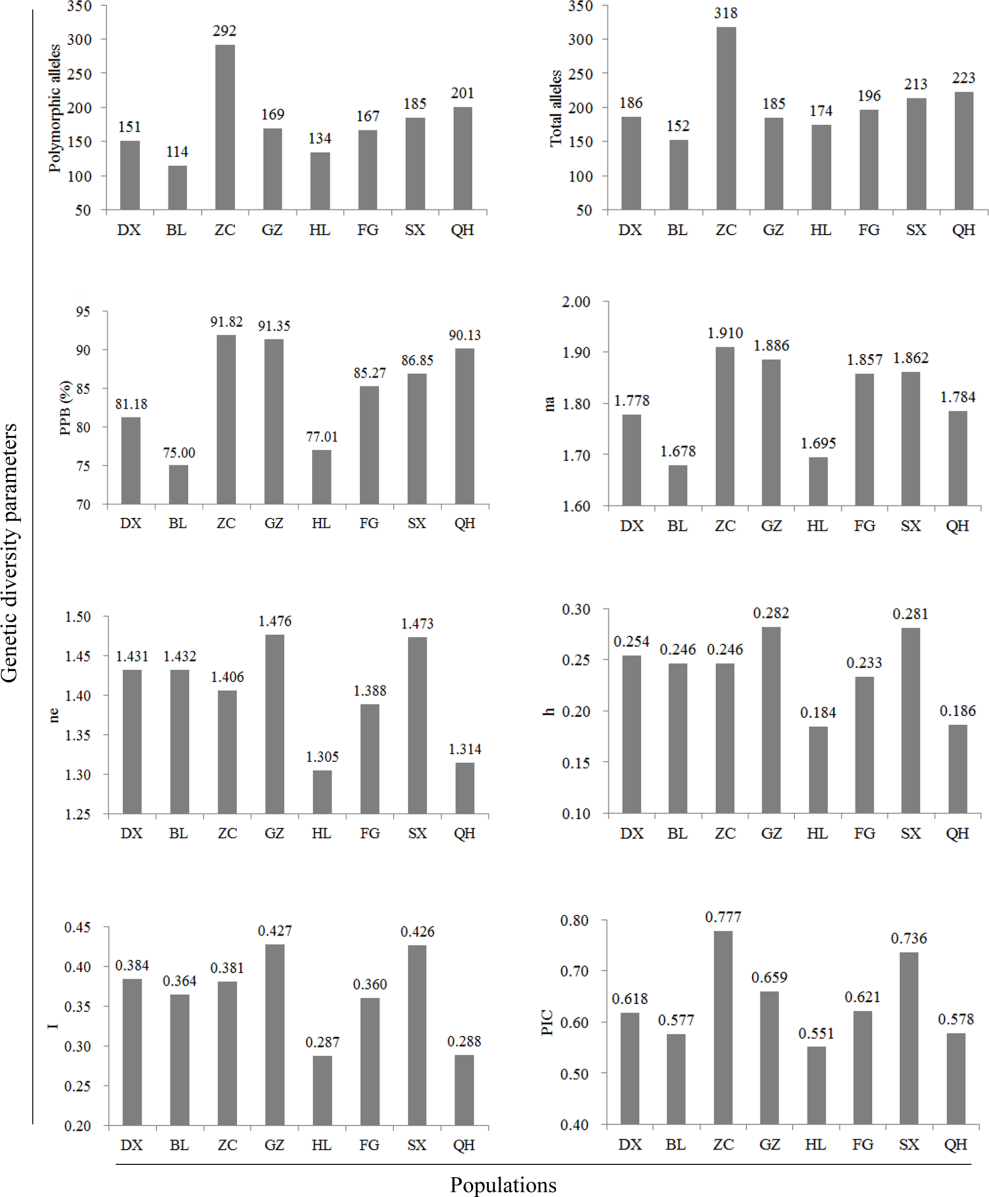
Comparisons of genetic diversity parameters among eight populations of wild rice. PPB, na, ne, h, I, and PIC indicate percentage of polymorphic bands, number of alleles, effective number of alleles, Nei's gene diversity, Shannon's information index, and polymorphism information content, respectively. See Table 1 for details of populations.

The SSR markers revealed a high level of genetic diversity (polymorphic allele = 176.6, total alleles = 205.9, PPB = 84.83%, na = 1.806, ne = 1.403, h = 0.239, I = 0.365, and PIC = 0.640) over the eight populations. Polymorphic alleles (292), total alleles (318), PPB (91.82%), na (1.910), and PIC (0.777) exhibited the highest level in the ZC population than in the other populations (Fig 4). The ne, h, and I in the GZ and SX populations were higher than in the other populations. The high number of effective alleles (ne) highlights the importance of alleles in the GZ and SX populations. Polymorphic alleles, total alleles, PPB, na, and PIC were lowest in the HL and BL populations. These results indicate remarkable differences in the genetic diversity of common wild rice populations. The ZC, GZ, and SX populations possess higher levels of genetic diversity, while the HL and BL populations have lower levels of genetic diversity than do the other populations.

**Fig 5:**
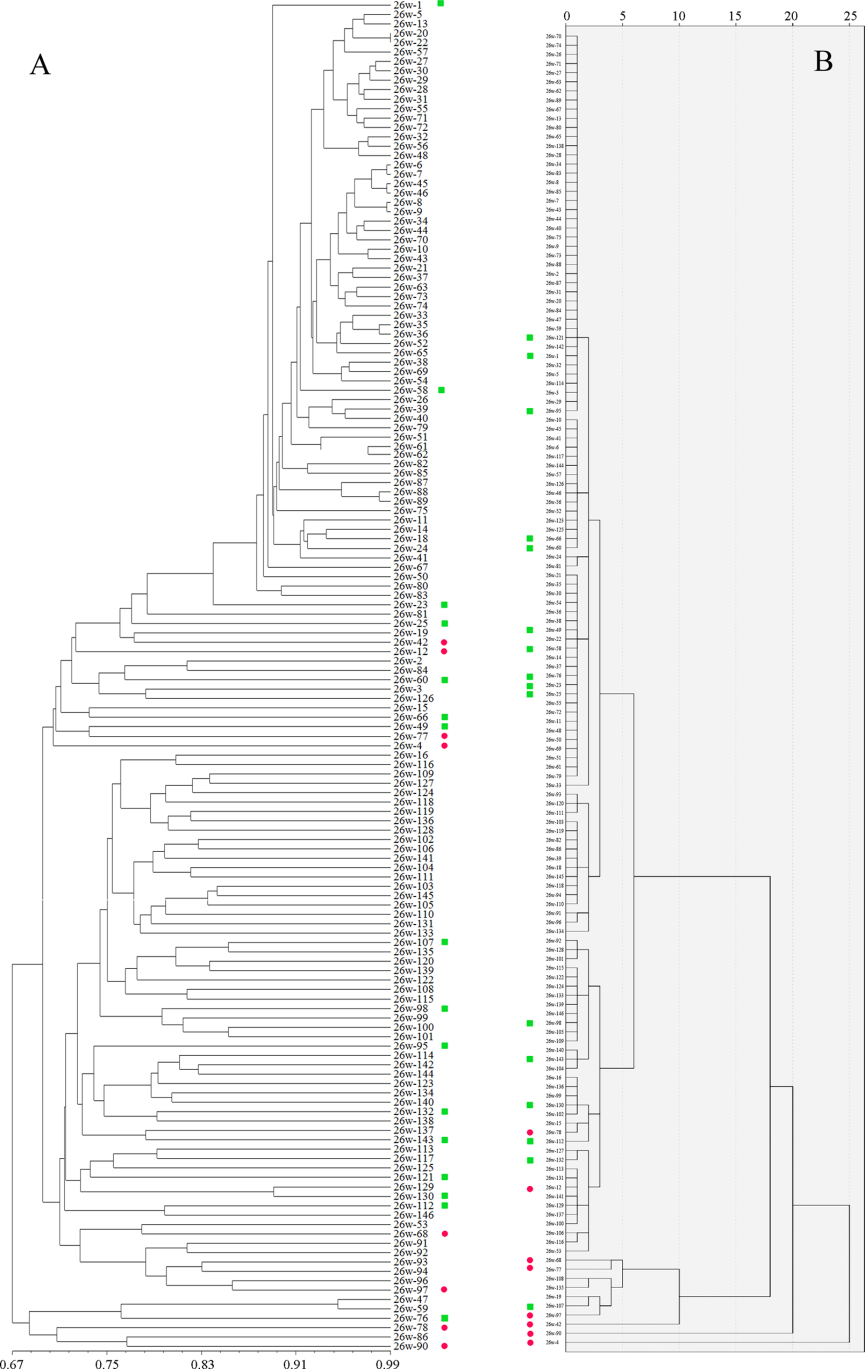
UPGMA dendrogram of the Zengcheng (ZC) wild rice population based on phenotypic and genotypic data. A and B show dendrograms based on phenotypic and genotypic data, respectively. Red dots show accessions selected for the core collection with high genetic distance and green squares denote accessions selected for the core collection with a gradual increase in genetic distance (from low to high). Numbers in vertical rows represent accessions selected as core collection. Numbers on the lower side of A represent genetic relevance and numbers on the upper side of B represent genetic distance. See Table 1 for details of populations.

Moreover, we conducted a combined analysis of morphological and molecular data, and the results were nearly consistent with the separate analyses of morphological and molecular data (Figs D-K in S1 File), i.e., both approaches identified similar individuals with high genetic distance. For example, six accessions of the ZC population, namely 26w-4, 26w-42, 26w-68, 26w-77, 26w-90, and 26w-97, exhibited a high genetic distance in both the combined and separate analyses of the morphological and molecular data (Fig 5, Fig G in S1 File). However, 26w-12 and 26w-78 exhibited high genetic distance in the molecular analysis, but low genetic distance in the combined analysis. The comparison of combined and separate analyses of data revealed that the genetic distance of separate clusters conformed to both molecular and morphological data.

### Relationships between the populations

**Fig 6:**
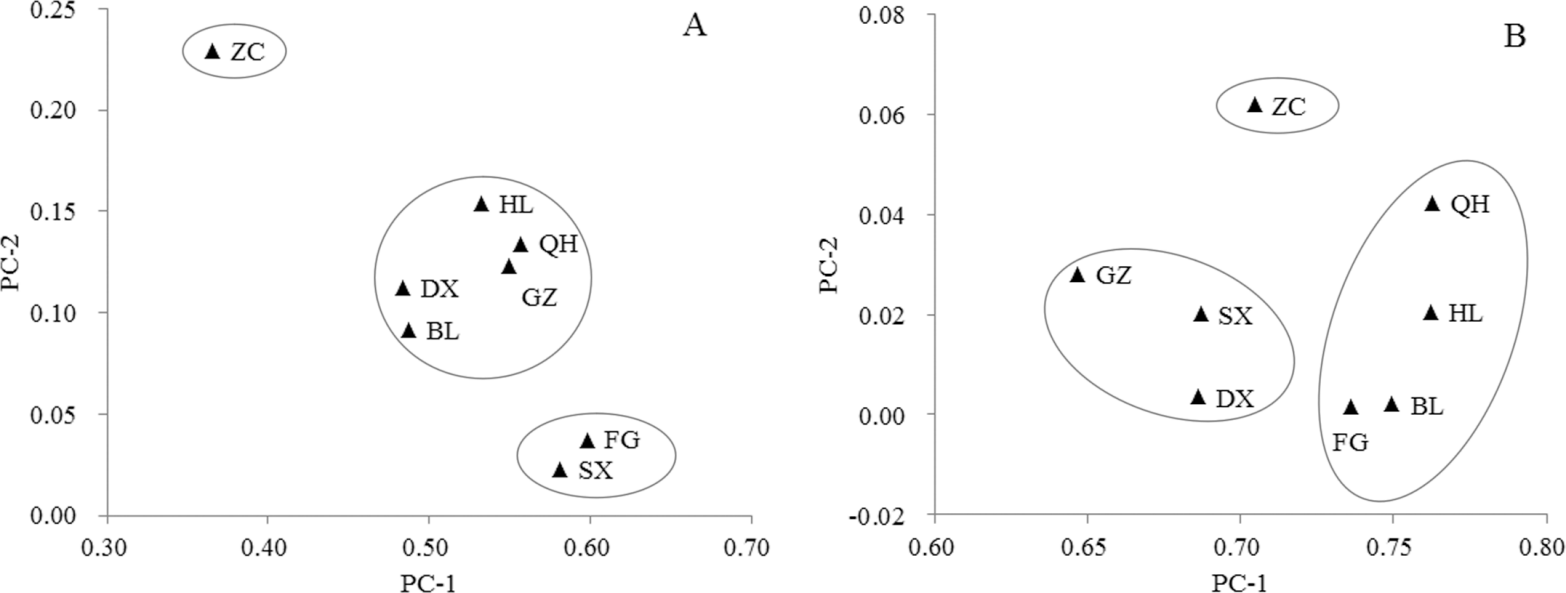
PCA relationship among eight populations of common wild rice. A and B represent PCA relationships among populations based on phenotypic and genotypic data, respectively. See Table 1 for details of populations.

We then performed principle component analysis (PCA) based on phenotypic and genotypic data (Fig 6). PCA revealed that the eight populations could be divided into three groups based on phenotypic data. The ZC population constituted one group, the FG and SX populations belonged to a second group, and the five remaining populations belonged to a third group. We also identified three distinct groups in the PCA distribution of genotypic data, with the ZC population falling into one group, GZ, SX, and DX into a second, and QH, HL, BL, and FG into a third. We calculated the Nei's genetic identity and genetic distance among the eight populations using POPGENE, and found that the genetic distance between accessions in the ZC population was greater than that between accessions in the other populations (S5 Table). These results are consistent with the PCA findings, which also clustered the ZC population into an independent group in both the phenotypic and genotypic PCA diagrams. Thus, the ZC population is of particular interest for plant breeders and germplasm conservation because it maintains the highest level of genetic diversity.

AMOVA revealed that molecular variation within populations (80.68%) was higher than among populations (19.32%) (Table 3). We determined the coefficient of gene differentiation (Gst, the proportion of total genetic diversity) and gene flow among wild rice populations (Table 4). The coefficient of genetic differentiation among populations was 0.482, and gene flow (Nm) was estimated as 0.538, indicating that there was a relatively low level of allele migration among populations. Furthermore, the total gene diversity (Ht = 0.309) among the eight populations and gene diversity within populations (Hs = 0.160) was low.

**Table 3:** Analysis of molecular variance (AMOVA) of genetic diversity of eight wild rice populations.

Source of variation	Sum of squares	Variance of components	Percentage of variation (%)
Among populations	38.60	0.07	19.32
Within populations	185.75	0.28	80.68
Total	224.35	0.35	100.00

**Table 4:** Genetic differentiation and estimation of gene flow in the wild rice populations using SSR loci.

	Ht	Hs	Gst	Nm
Mean	0.309	0.160	0.482	0.538
SD	0.034	0.012		

Ht: Total gene diversity; Hs: Gene diversity within populations; Gst: Coefficient of gene differentiation; Nm: estimate of gene flow from Gst; Nm = 0.5(1—Gst)/Gst; SD: Standard deviation.

Gene differentiation: genetic differentiation among populations and within a population.

Gene flow: an indicator of allele movement among and within populations.

### Development and effectiveness of core collections of wild rice

To develop a core collection, we constructed dendrograms based on separate and combined analyses of phenotypic and genotypic data. The dendrograms based on separate analyses revealed a greater genetic distance than did those based on the combined analysis of data. To validate that the accessions were representative of the entire population, the accessions with a high genetic distance (3 for phenotypic and 0.76 for genotypic data) were selected as the core sets from phenotypic and genotypic dendrograms (Fig 5, red dots), and 16 accessions were selected from the entire population by hierarchical cluster analysis (Fig 5, green squares). Similarly, we developed another seven core collections from different populations using genetic distance of phenotypic and genotypic dendrograms (Figs L-R in S1 File). Further, we used QGAStation 2.0 to construct the core collections (S6 Table). Venn analysis revealed that 60 similar core collections with high genetic distance were selected by both methods (Fig S in S1 File). For example, the twelve accessions in the ZC population (26w-1, 26w-4, 26w-12, 26w-25, 26w-49, 26w-60, 26w-68, 26w-77, 26w-78, 26w-90, 26w-95, and 26w-98) exhibit the greatest genetic distance and were selected as core collections by both methods. Other accessions with low genetic distances were randomly selected by the software. Some important individual accessions were not selected during the development of a core collection by QGAStation software. Therefore, we preferentially constructed core collections on the basis of genetic distance and hierarchical cluster analysis in the present study. Furthermore, some accessions exhibited agronomically desirable morphological traits, such as the non-shattering trait (4w-102 and 4w-103), which were directly selected as core sets (Fig L in S1 File, red squares). Finally, a total of 130 core accessions were selected from the 885 accessions derived from the eight populations (S7 Table, Fig T in S1 File). The size of the core collection varied from 12 to 20% of accessions in a population, with a mean ratio of 14.7% for all eight populations (Table 1). This strategy to develop a core collection retained the high genetic diversity of the entire pool of wild rice populations examined.

**Fig 7:**
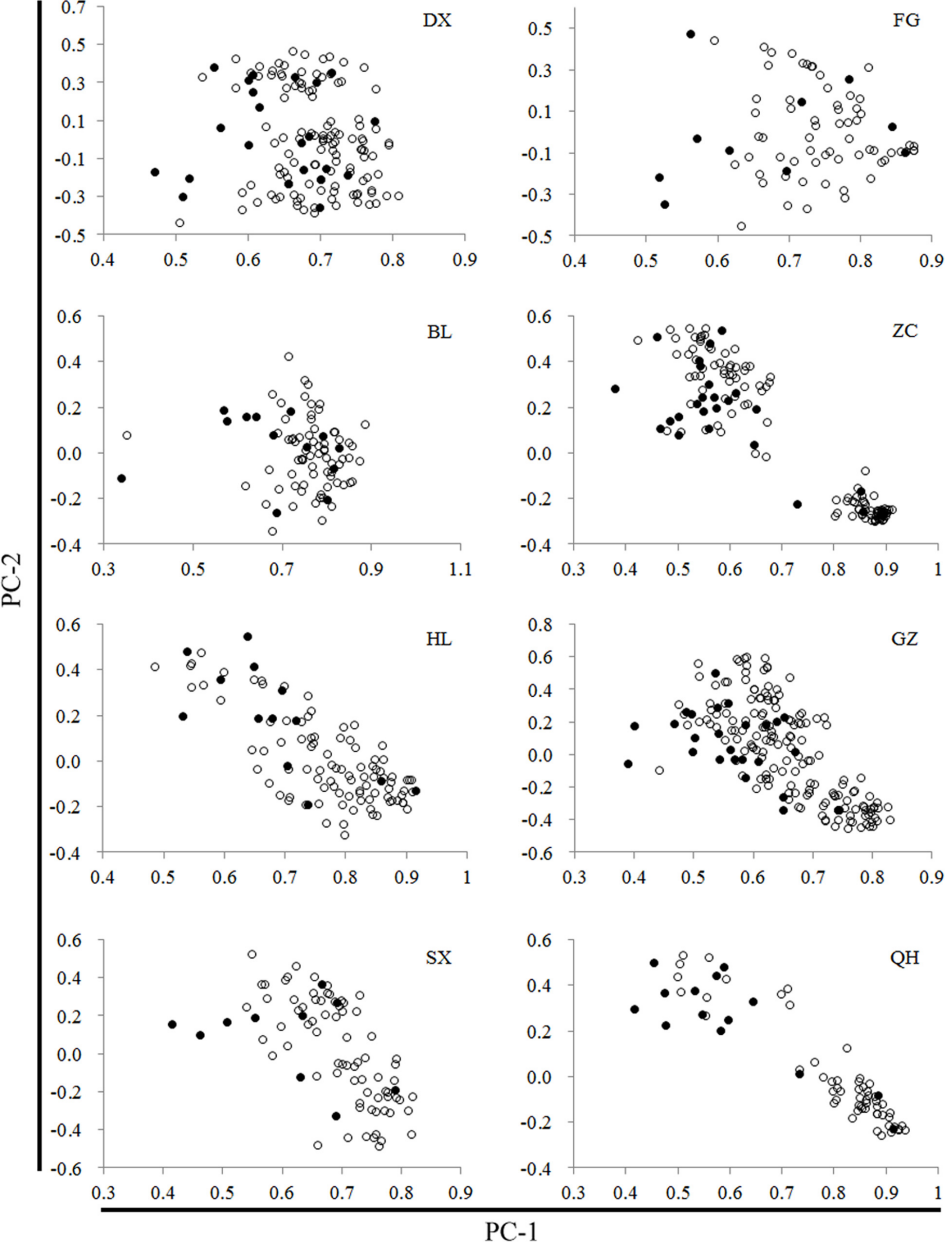
PCA distribution graphs of wild rice populations and core collections. Hollow dots represent the entire population and solid dots represent the accessions selected as core collection in each population. See Table 1 for details of populations.

Genetic diversity parameters were observed to estimate the coverage of core collections constructed from the entire common wild rice germplasm. The whole set of 36 SSR markers detected an average of 206.5 and 195.5 alleles in each population and core collection, respectively. Core collections retained 94.7% of alleles found in the whole germplasm. Alleles in the core collections were present in between 91.5 and 99.5% of the eight populations, and the coverage was above 90% of the alleles detected in the whole germplasm. The average number of alleles ranged from 1.338 for the RM201 marker to 1.969 for the RM413 marker, with an average of 1.806. PIC ranged from 0.235 for the RM201 marker to 0.754 for the RM283 marker, with an average of 0.640 (S4 Table). To examine differences between each core collection and its corresponding population, we then calculated *X* ^*2*^, the means, and standard errors for the five genetic parameters shown in Table 5. The differences between the means of the core and entire populations were non-significant for all five components of genetic diversity (Table 5). We constructed PCA distribution graphs using the first and second PCA scores (Fig 7). The core sets were distributed uniformly in the PCA distribution graph. These results suggest that the core collections maintained a high level of genetic diversity and were representative of the entire population.

**Table 5:** Genetic diversity comparison between an entire population and the core collection based on 36 marker loci.

Population	Groups	Number of alleles^a^	na	ne	h	I	PIC
Dongxiang (DX)	Entire population	186	1.778 (0.392)	1.431 (0.363)	0.254 (0.186)	0.384 (0.257)	0.618 (0.208)
	Core collection	185	1.757 (0.409)	1.455 (0.367)	0.264 (0.189)	0.394 (0.262)	0.628 (0.206)
	Allele coverage (%)	99.46	98.82	101.68	103.94	102.60	101.62
Boluo (BL)	Entire population	159	1.830 (0.377)	1.413 (0.365)	0.245 (0.187)	0.374 (0.255)	0.577 (0.211)
	Core collection	148	1.723 (0.449)	1.437 (0.385)	0.252 (0.199)	0.376 (0.278)	0.580 (0.211)
	Allele coverage (%)	93.08	94.15	101.70	102.86	100.53	100.52
Zengcheng (ZC)	Entire population	318	1.910 (0.274)	1.406 (0.347)	0.246 (0.176)	0.381 (0.238)	0.777 (0.083)
	Core collection	299	1.879 (0.296)	1.480 (0.342)	0.284 (0.168)	0.431 (0.223)	0.799 (0.078)
	Allele coverage (%)	94.03	98.38	105.26	115.45	113.12	102.83
Gaozhou (GZ)	Entire population	185	1.886 (0.274)	1.476 (0.339)	0.282 (0.168)	0.427 (0.224)	0.659 (0.165)
	Core collection	184	1.860 (0.312)	1.502 (0.338)	0.294 (0.167)	0.442 (0.225)	0.669 (0.173)
	Allele coverage (%)	99.46	98.62	101.76	104.26	103.51	101.52
Huilai (HL)	Entire population	174	1.695 (0.422)	1.305 (0.356)	0.184 (0.184)	0.287 (0.254)	0.551 (0.234)
	Core collection	163	1.631 (0.445)	1.318 (0.348)	0.193 (0.183)	0.296 (0.257)	0.550 (0.268)
	Allele coverage (%)	93.68	96.22	101.00	104.89	103.14	99.82
Fogang (FG)	Entire population	196	1.857 (0.351)	1.388 (0.355)	0.233 (0.183)	0.360 (0.250)	0.621 (0.181)
	Core collection	178	1.750 (0.440)	1.410 (0.360)	0.250 (0.190)	0.370 (0.260)	0.608 (0.237)
	Allele coverage (%)	90.82	94.24	101.59	107.30	102.78	97.91
Suixi (SX)	Entire population	211	1.862 (0.346)	1.473 (0.349)	0.281 (0.173)	0.426 (0.235)	0.736 (0.075)
	Core collection	203	1.754 (0.432)	1.504 (0.382)	0.286 (0.196)	0.421 (0.273)	0.743 (0.067)
	Allele coverage (%)	96.21	94.20	102.10	101.78	98.83	100.95
Qionghai (QH)	Entire population	223	1.784 (0.302)	1.314 (0.365)	0.186 (0.189)	0.288 (0.256)	0.578 (0.230)
	Core collection	204	1.722 (0.327)	1.291 (0.339)	0.187 (0.169)	0.299 (0.227)	0.583 (0.286)
	Allele coverage (%)	91.48	96.52	98.25	100.54	103.82	100.87
Mean	Entire population	205.9 (50.7)	1.806 (0.081)	1.403 (0.061)	0.239 (0.035)	0.365 (0.050)	0.640 (0.075)
	Core collection	195.5 (42.9)	1.760 (0.074)	1.425 (0.076)	0.252 (0.038)	0.380 (0.052)	0.645 (0.081)
	Allele coverage (%)	94.96	97.41	101.53	105.04	103.84	100.83

^a^Total alleles detected at the 36 marker loci.

na, ne, h, I, and PIC indicate the number of alleles, effective number of alleles, Nei's gene diversity, Shannon's information index, and polymorphism information content, respectively.

The values in parentheses are standard errors.

There was a non-significant difference between the core collection and entire population for these genetic diversity parameters.

## Discussion

### Evaluation of genetic diversity of common wild rice and development of a core collection

We subjected both SSR markers and morphological data to genetic diversity analysis and established core collections from eight wild rice populations. We detected high levels of genetic diversity in common wild rice populations from Guangdong province. For example, the ZC population had the highest total number of alleles, polymorphic alleles, percentage of polymorphic bands, average number of alleles, and polymorphism information content, while the GZ and SX populations had higher effective numbers of alleles, Nei's gene diversity, and Shannon's information index than the other populations. The high number of effective alleles indicates the importance of alleles in the ZC, GZ, and SX populations. These three populations from Guangdong province had higher levels of genetic diversity than the other populations. These results are in agreement with earlier studies [8,13–15]. The GZ population had high morphological and genetic diversity, and is an important source of genetic differentiation and diversity in Chinese wild rice [27,33]. The ZC, GZ, and SX populations exist within the same water system (Zhujiang River), and the influence of the water system may be one reason for the gene flow between these populations [13,47]. Further, the DX population from Jiangxi province had the lowest level of diversity compared to the other populations, possibly because of the disappearance of subpopulations due to habitat loss. Similarly, the DX population was found to have a low level of genetic diversity in a previous study [10].

Morphological and molecular data can be analyzed separately or in combination to determine genetic diversity based on genetic distance and hierarchical cluster analysis [39,48]. Here, the number of molecular markers used was greater than the number of morphological traits examined. Thus, if the morphological and molecular data were combined for phylogenetic analysis, the weight of the molecular marker data would be greater than that of the morphological data. The dendrograms generated in this study were very similar for the separate and combined analyses; however, the combined analysis failed to detect some important individual accessions that showed high genetic distance in the separate analyses. For example, accession 4w-102 had agronomically important traits such as high seed setting percentage and number of filled spikelets per panicle, but was not selected in the combined analysis of phenotypic and genotypic data (Figs D and L in S1 File). Our comparative analyses also revealed that analyzing the molecular and morphological data separately was more effective for constructing a core collection of wild rice. Further, ANOVA and AMOVA of morphological and molecular data indicated that the molecular data were superior to the morphological data. Therefore, we analyzed the genetic distances and hierarchical clustering of these two data sets separately in this study. Similarly, the morphological and molecular characteristics of common wild rice were analyzed separately in previous studies [20,38,49]. However, these studies did not compare combined and separate analysis of morphological and molecular data. ANOVA analysis of the morphological data revealed that ten quantitative traits exhibited significant differences (p<0.05) among populations. The BL population had the highest mean CV for the ten quantitative traits. Only a few reports have been published regarding this population of common wild rice. Some morphological traits, such as leaf color, flowering rate, number of spikelets per panicle, and seed setting percentage showed differences among the eight populations. The SX population, from the lower north latitude, had a higher proportion of dark green leaves, but a lower flowering rate, creeping growth habit, number of spikelets per panicle, and seed setting percentage than did other populations. Differences in morphological traits of common wild rice were significantly correlated with latitude. These results are in agreement with earlier studies showing that differences in growing conditions could lead to gradual changes in morphological traits of common wild rice [46,50].

### A core collection to capture maximum genetic diversity of common wild rice

Three methods are commonly used to develop core collections, and each of these methods has advantages and disadvantages [26,34,51]. The genetic diversity of core collections assembled using the first method, in which software is used to establish the core collection, does no need to be validated if an appropriate sampling proportion (i.e., 5–30%) has been achieved. However, individuals with excellent traits that would be useful genetic resources could easily be overlooked if using only software [51]. The second method, which involves developing a hierarchical core collection system to retain the main types of alleles present in a population, is the most effective method for selecting core collections and allows for the flexible use of genetic resources [34]; however, it is technically challenging and time consuming. Nonetheless, this approach retains accessions with excellent traits [26]. In the third method, the un-weighted pair group method of arithmetic average (UPGMA), genetic relevance and genetic distance are the major parameters used to develop core collections. Using this approach, accessions with excellent traits and high genetic distance were selected as core collections directly [17,26,52]. For instance, a mini core collection consisting of 189 varieties of *Oryza sativa* was developed in China [34] and a core set of 701 accessions was developed that accounted for approximately 10% of accessions from the total North-Eastern region of India, representing 99.9% of the allelic diversity [53]. Furthermore, core collections consisting of 150 accessions were selected from the 2262 accessions present in Ting’s collection of cultivated Chinese rice, and retained 100% of the phenotypic characteristics of all the collections [54].

In this study, we used the UPGMA and genetic distance to develop the core collection. We validated the percentage of alleles retained, the genetic diversity, and the PCA between the core and entire collection to ensure the genetic diversity of the core collection. Common wild rice has some excellent traits, such as those exhibited by the 4w-102 and 4w-103 accessions, and these accessions were not selected when using the first method described above (i.e., software).

An ideal core collection encompasses the maximum genetic diversity of the entire germplasm with minimum repetitiveness. Various methods are used to construct a core collection, including phenotypic, isozyme, protein, SNP, and DNA marker data. However, there is no universally accepted method for constructing a core collection; all methods have advantages and disadvantages. Phenotypic or genotypic traits have been recognized as useful parameters for developing core collections [49,55]. Therefore, a combination of both phenotypic and genotypic data is thought to be more useful than either one of these individually when constructing a core collection [38]. In this study, representatives of the established core collection were identified by observing both phenotypic and genotypic descriptors. Further, cluster analysis was performed to develop an efficient core collection by selecting accessions with a range of genetic distances. This strategy maintained the genetic diversity of the entire population. Thus, phenotypic and genotypic analyses are useful for constructing a core collection of common wild rice germplasm.

The sampling proportion of a core collection is important for selecting a suitable sampling percentage to obtain the maximum genetic diversity and the maximum range of geographical types. Usually, 5–30% of the sampling percentage is selected from the entire germplasm [28,56]. In this study, we selected 130 accessions as the core collection and the average ratio of selected core accessions to the total number of accessions in the eight populations was 14.69%. The coefficient of variation between an entire population and the core collection was non-significant, indicating that the core collection retained a high level of genetic diversity.

### Importance of *ex situ* conservation of common wild rice

Geographical isolation is one of the barriers that blocks the introgression of wild species and generates differentiation after long-term adaptive evolution [57,58]. All the populations used in this study were native to different geographical areas of China. Genetic diversity was higher among different populations than within them.

*Ex situ* conservation provides germplasm that breeders can use to improve elite cultivars. Although there are hundreds of natural populations of wild rice, conservation strategies favor the *ex situ* conservation of common wild rice. Evaluating genetic diversity is extremely important for the *ex situ* conservation of wild plants [59,60]. Considering the loss of natural habitats of common wild rice, *ex situ* conservation is the best strategy for preserving the genetic diversity of common wild rice. We have conserved (in complete isolation) accessions of common wild rice from three provinces of China in our wild rice germplasm garden (South China Agricultural University). We found that all of the conserved populations maintained a high level of genetic diversity in the last two decades. However, Xie et al. revealed that wild rice lost genetic diversity during *ex situ* conservation. Thus, *in situ* conservation is also required to preserve the genetic diversity of common wild rice [10].

In conclusion, we have assembled a core collection of common wild rice with abundant morphological and genetic diversity from different ecological regions of China and conserved this collection *ex situ*. Our strategy was highly successful in selecting representative accessions from the entire population based on phenotypic and genotypic data. In addition, we showed that individual analysis of morphological and molecular data is more effective than combined analysis of these data when constructing a core collection of wild rice. Furthermore, it is better to construct a core collection of wild rice based on UPGMA and genetic distance than on core collection construction software alone. The core collection not only represents the mean and variances but also the range of variables of the entire population. Therefore, morphological and genotypic data can be used to construct a highly representative core collection. To avoid inter- and intra-population introgression of wild rice, it is critical that the common wild rice germplasm be conserved (both *ex situ* and *in situ*) as core collections.

## Supporting Information

S1 File Development of a core collection based on phenotypic and genotypic data of common wild rice populations. *Ex situ* and *in situ* conservation of wild rice in China (**Figs A-C**). UPGMA dendrograms of the common wild rice populations from eight districts/counties based on combined phenotypic and genotypic data (**Figs D-K**). Dendrograms denoting UPGMA clustering analyses of the common wild rice populations from eight districts/counties based on phenotypic and genotypic data (**Figs L-R**). Conservation of the core collection of common wild rice (**Fig S**). Venn diagram representing the overlapping of core collections developed by QGA software and genetic distance (**Fig T**).
(PDF)

S1 Table Sampling information of common wild rice in China. (DOCX)

S2 Table List of SSR primers used in the present study. (DOCX)

S3 Table Analysis of variance (ANOVA) of ten morphological traits from eight populations. (DOCX)

S4 Table The genetic diversity detected by 36 marker loci in eight populations. (DOCX)

S5 Table Nei's genetic identity and genetic distance among eight populations. (DOCX)

S6 Table Comparison of the core collections developed by software and genetic distance. (DOCX)

S7 Table Morphological traits data of 130 accessions selected as the core collection. (DOCX)
