# Supplementary material for: Evaluation of Genetic Diversity and Development of a Core Collection of Wild Rice (Oryza rufipogon Griff.) Populations in China
Source: PLoS One. 2015 Dec 31;10(12):e0145990. doi: 10.1371/journal.pone.0145990 (PMC4703137; doi:10.1371/journal.pone.0145990)
Supplement: S1 File — Ex situ and in situ conservation of wild rice in China (Figs A-C). UPGMA dendrograms of the common wild rice populations from eight districts/counties based on combined phenotypic and genotypic data (Figs D-K). Dendrograms denoting UPGMA clustering analyses of the common wild rice populations from eight districts/counties based on phenotypic and genotypic data (Figs L-R). Conservation of the core collection of common wild rice (Fig S). Venn diagram representing the overlapping of core collections developed by QGA software and genetic distance (Fig T). (PDF) [file pone.0145990.s001.pdf]

Supporting Information

S1 File

Development of a core collection based on phenotypic and genotypic data of common wild rice populations

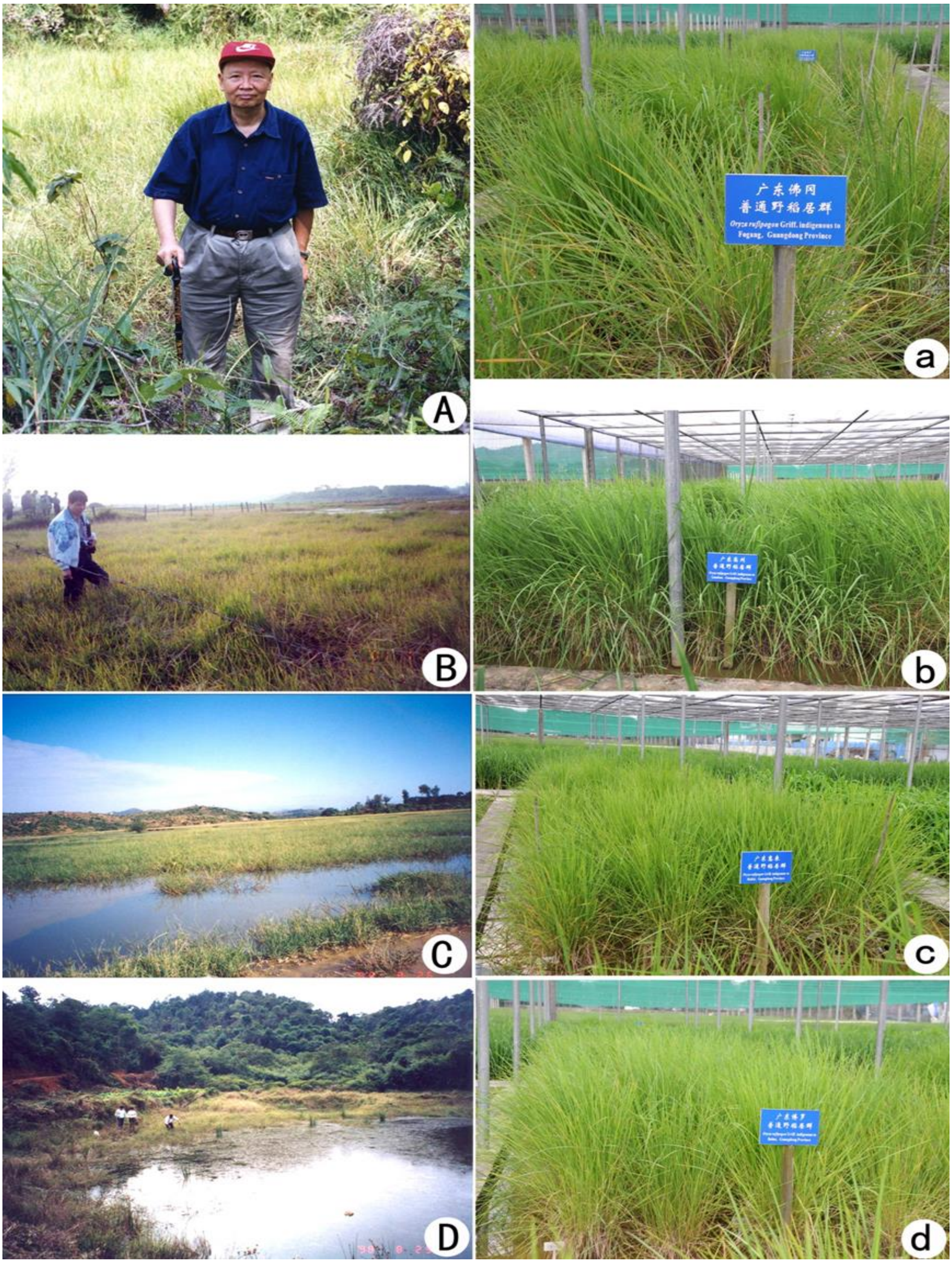

Figure A. ***Ex situ*** **and** ***in situ*** **conservation of wild rice in China.**

Uppercase letters represent natural habitats of wild rice, while lowercase letters represent *ex situ* conservation of wild rice.

A/a: Common wild rice at Fogang (Lu YG at Fogang *in situ* conservation); B/b, C/c and D/d represent the wild rice germplasm at Gaozhou, Huilai and Boluo, respectively.

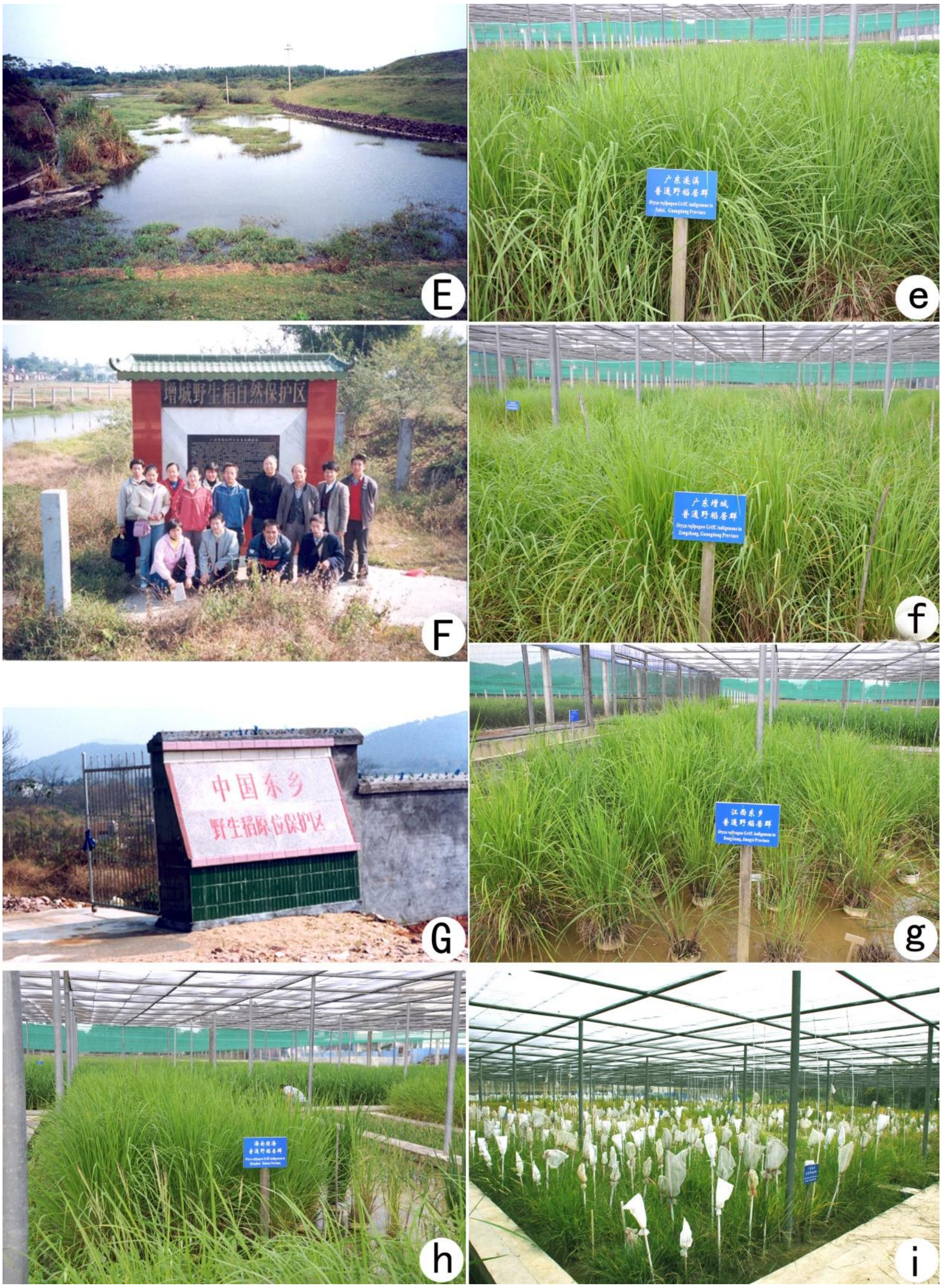

Figure B. *Ex situ* and *in situ* conservation of wild rice in China.

Uppercase letters represent natural habitats of wild rice, while lowercase letters represent *ex situ* conservation of wild rice.

E/e, F/f, and G/g represent common wild rice at Suixi, Zengcheng (Lu YG, Liu XD and other team members at Zengcheng *in situ* conservation) and Dongxiang, respectively. h: Qionghai *ex situ* conservation; i: Paper bags were used to ensure self-fertilization.

Note: Panels B, E and F are adapted from Li JQ (2011)

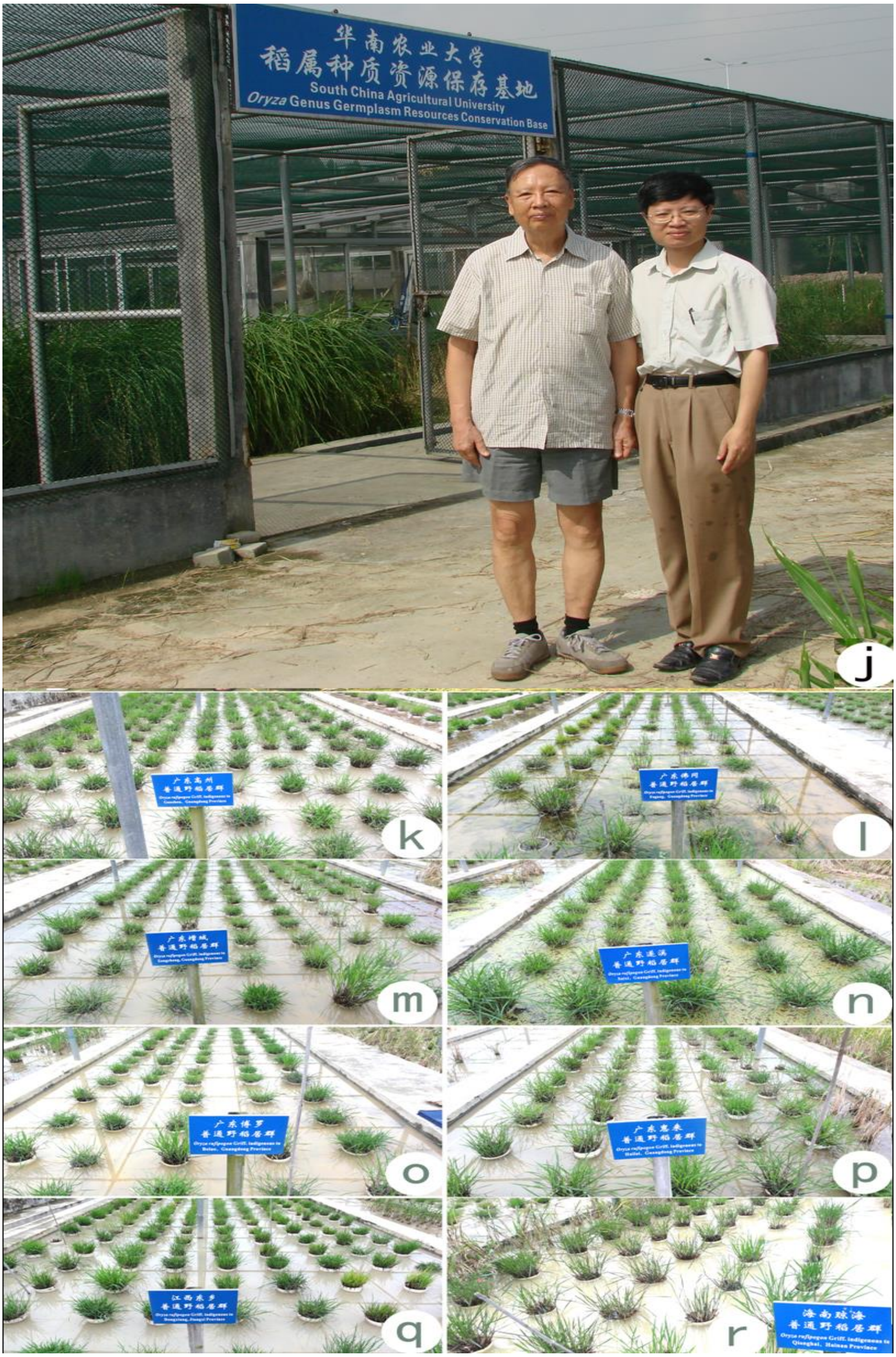

Figure C. ***Oryza*** **genus germplasm resources conservation base (*****ex situ*****) at South China Agricultural University.**

j: Wild rice germplasm garden of South China Agricultural University (Lu YG and Liu XD typical to list from left to right); k-r wild rice germplasm collections of Gaozhou, Fogang, Zengcheng, Suixi, Boluo, Huilai, Dongxiang and Qionghai, respectively, at the early growth stages.

- Our research group has published a few articles and a book chapter (Li 2011) on wild rice in other journals such as Shi et al. (2009), Wei et al. (2010) and Tong et al. (2011).

References

Li JQ (2011) Wild Rice: Identification, used and Conservation. In: Ryan E. Davis (eds) Wild Plants: identification, used and conservation. Nova Science publishers, Inc. New York, pp 269-287.

Shi LG, Liu XD, Liu B, Zhao XJ, Wang L, Li JQ* and Lu YG*. Identifying neutral allele S^b at pollen-sterility loci in cultivated rice with *Oryza rufipogon* origin. Chinese Sci Bull, 2009, 54: 3813-3821. (doi: 10.1007/s11434-009-0571-y).

Tong JF, Li YH, Yang YX, Shahid MQ, Chen ZX, Wang L, Liu XD* and Lu YG*. Molecular evolution of rice S_5^n and functional comparison among different sequences. Chinese Sci Bull, 2011, 56.(doi: 10.1007/s11434-011-4534-8).

Wei CM, Wang L, Yang YX, Chen AX, Shahid MQ, Li Jinquan, Liu XD*, Lu YG*. Identification of an S_5^n allele in *Oryza rufipogon* Griff. and its effect on embryo sac fertility. Chinese Science Bulletin. 2010, 55(13):1255-1262. (doi: 10.1007/s11434-010-0154-y).

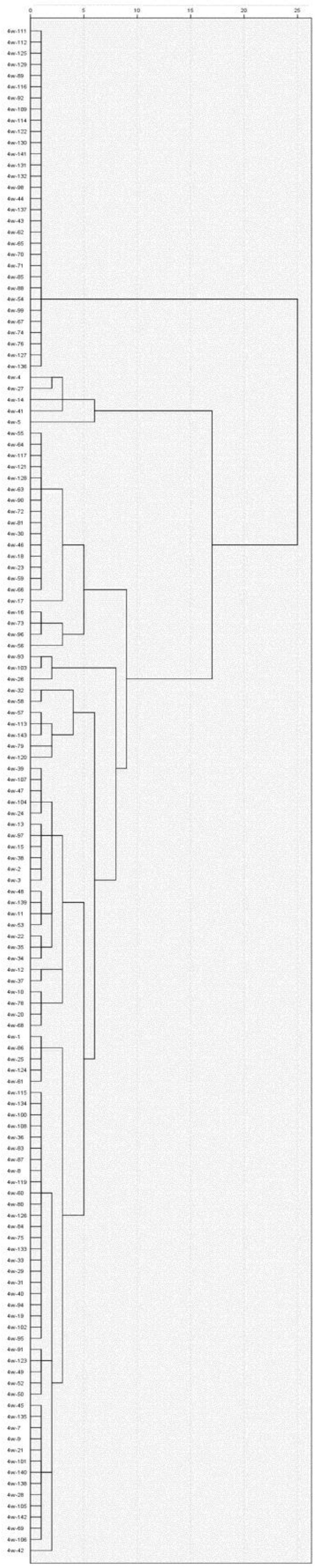

Figure D. UPGMA dendrogram of the Dongxiang common wild rice population based on combined phenotypic and genotypic data.

Numbers on the left side refer to accessions. Numbers on the top refer to genetic distance.

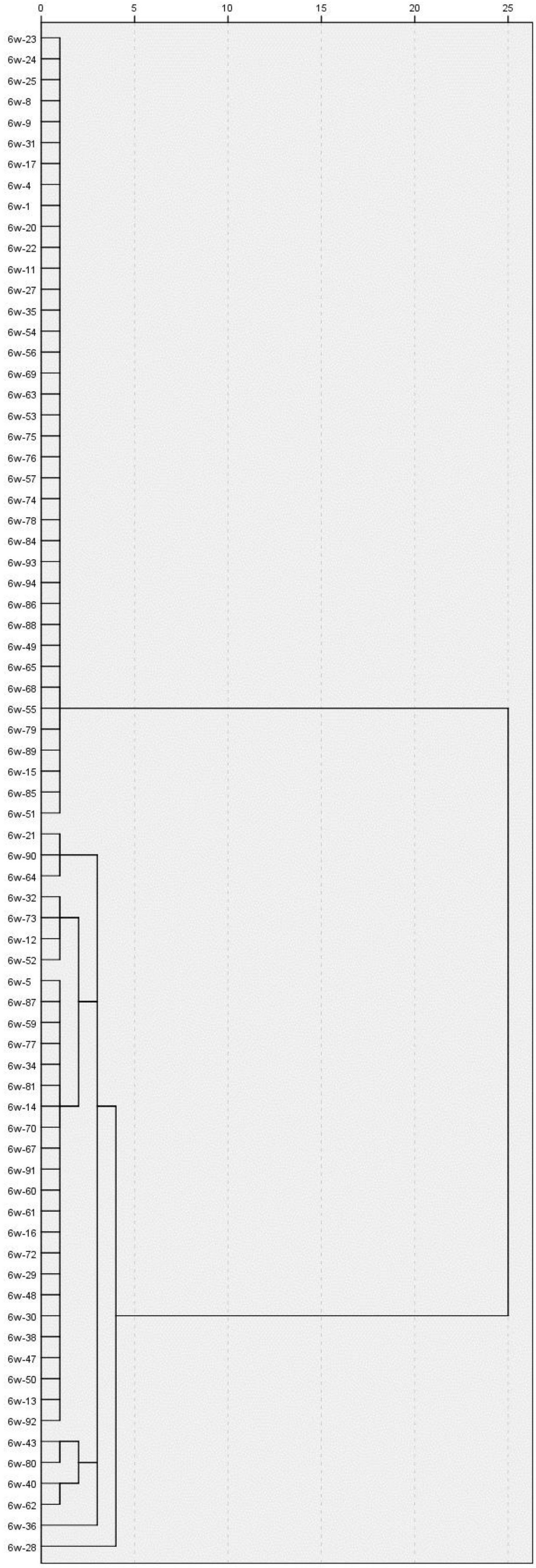

Figure E. UPGMA dendrogram of the Fogang common wild rice population based on combined phenotypic and genotypic data.

Numbers on the left side refer to accessions. Numbers on the top refer to genetic distance.

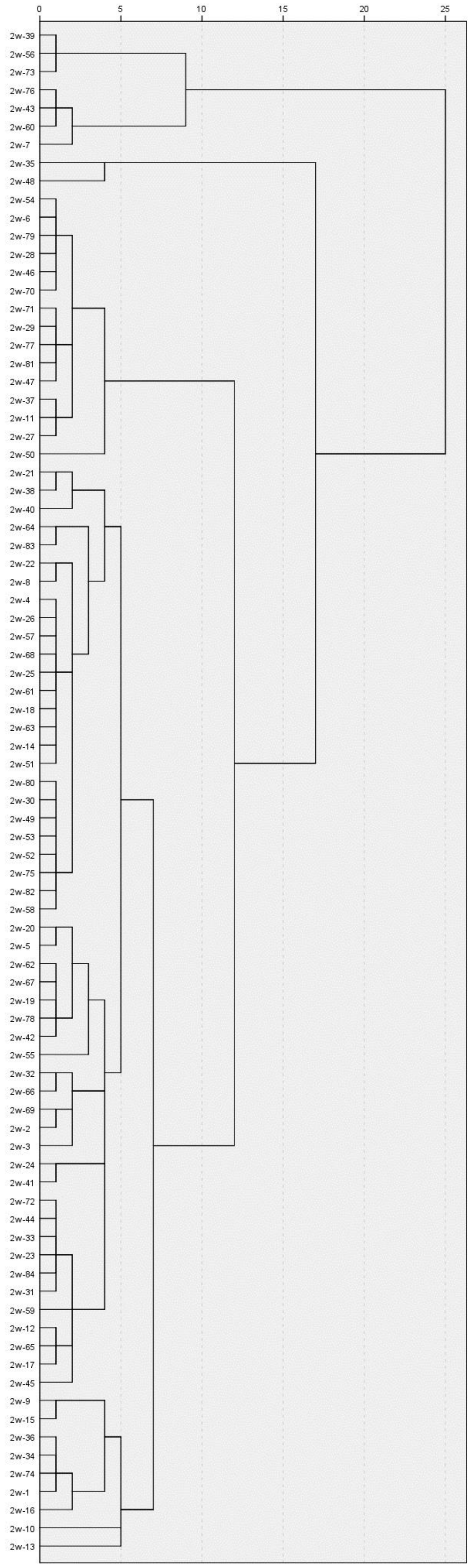

Figure F. UPGMA dendrogram of the Boluo common wild rice population based on combined phenotypic and genotypic data.

Numbers on the left side refer to accessions. Numbers on the top refer to genetic distance.

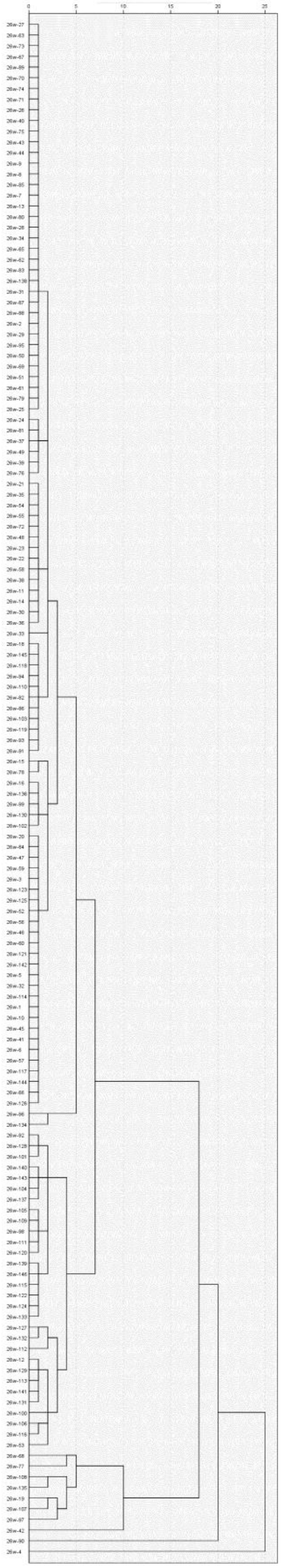

Figure G. UPGMA dendrogram of the Zengcheng common wild rice population based on combined phenotypic and genotypic data.

Numbers on the left side refer to accessions. Numbers on the top refer to genetic distance.

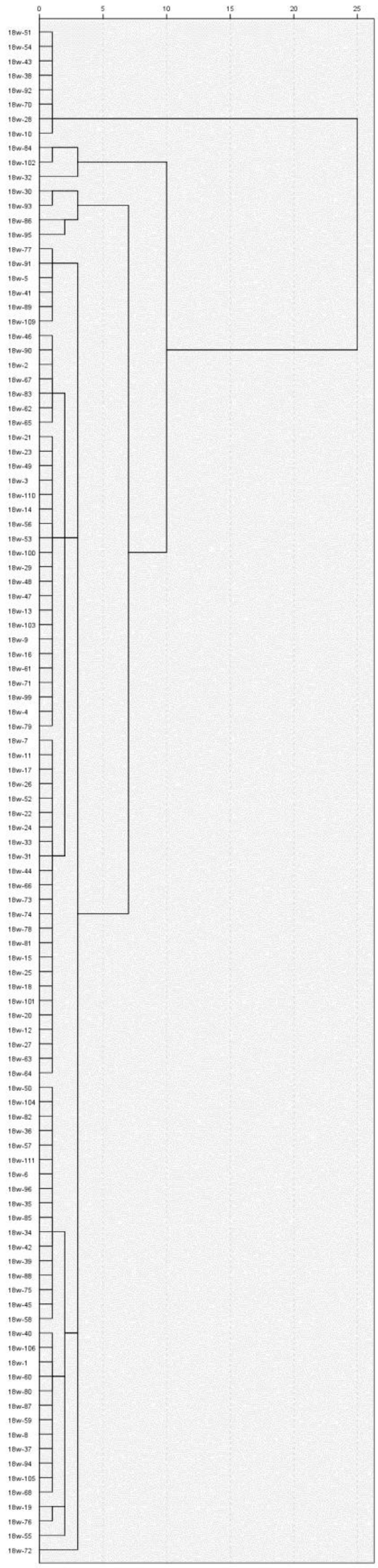

Figure H. UPGMA dendrogram of the Huilai common wild rice population based on combined phenotypic and genotypic data.

Numbers on the left side refer to accessions. Numbers on the top refer to genetic distance.

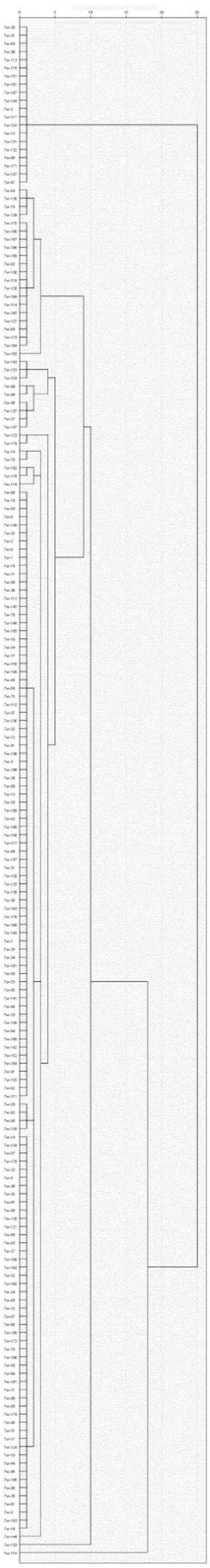

Figure I. UPGMA dendrogram of the Gaozhou common wild rice population based on combined phenotypic and genotypic data.

Numbers on the left side refer to accessions. Numbers on the top refer to genetic distance.

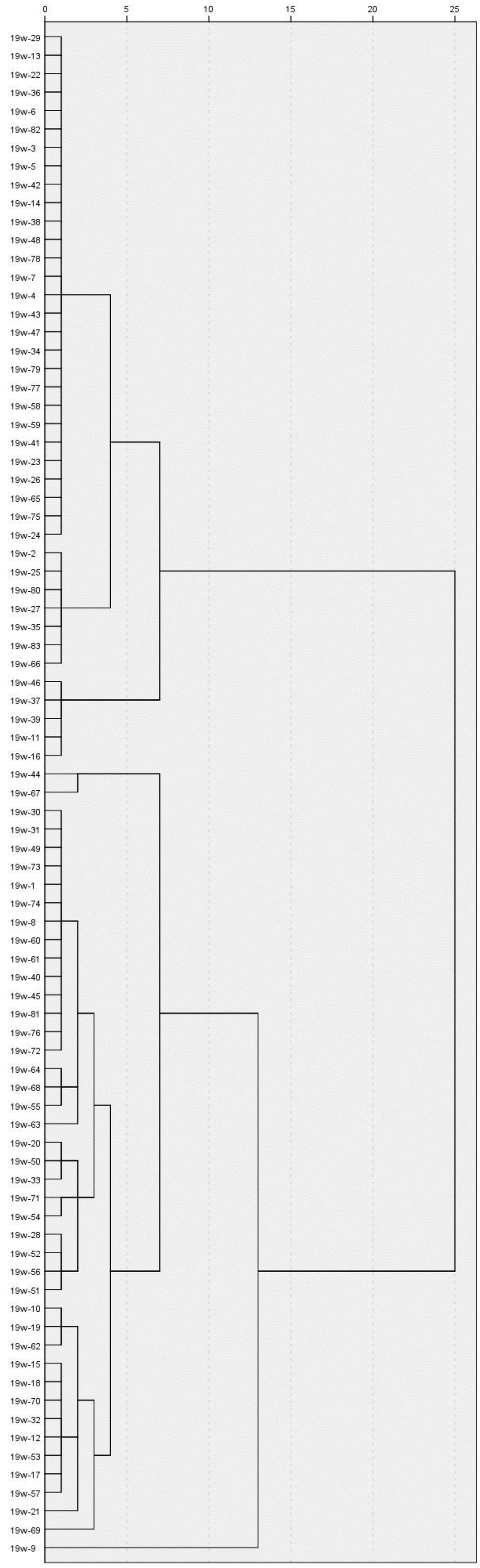

Figure J. UPGMA dendrogram of the Suixi common wild rice population based on combined phenotypic and genotypic data.

Numbers on the left side refer to accessions. Numbers on the top refer to genetic distance.

Figure K. UPGMA dendrogram of the Qionghai common wild rice population based on combined phenotypic and genotypic data.

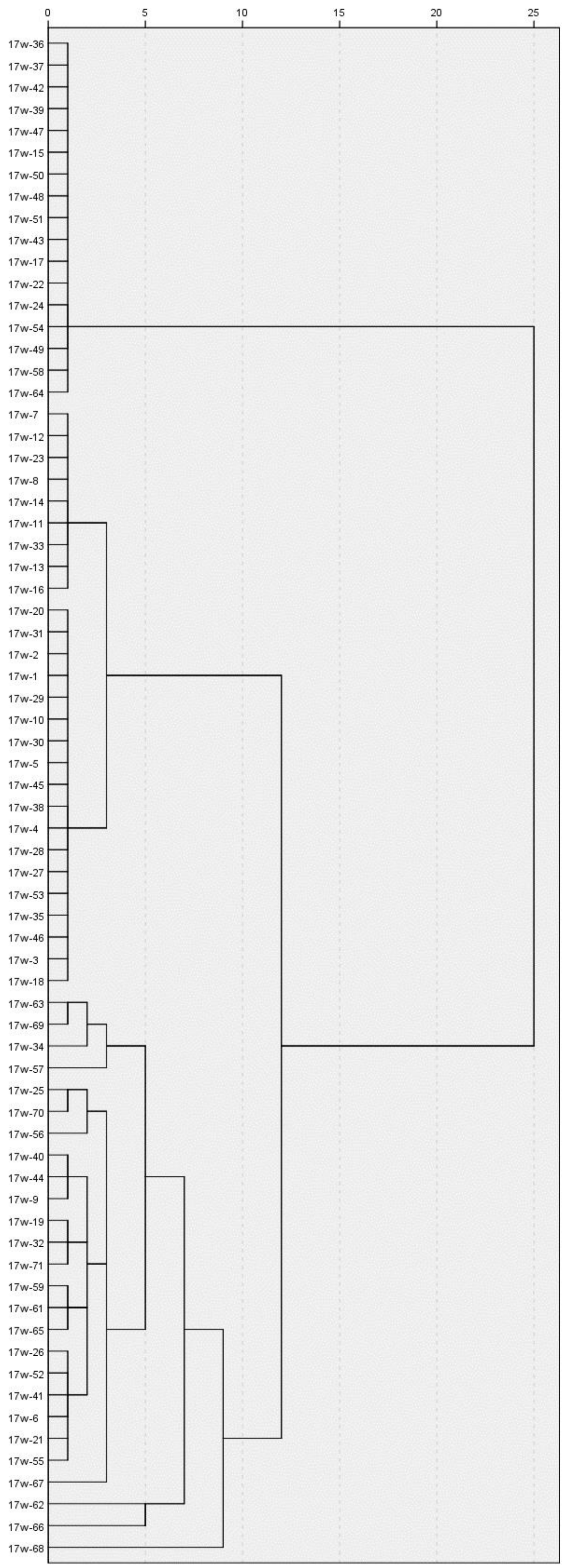

Numbers on the left side refer to accessions. Numbers on the top refer to genetic distance.

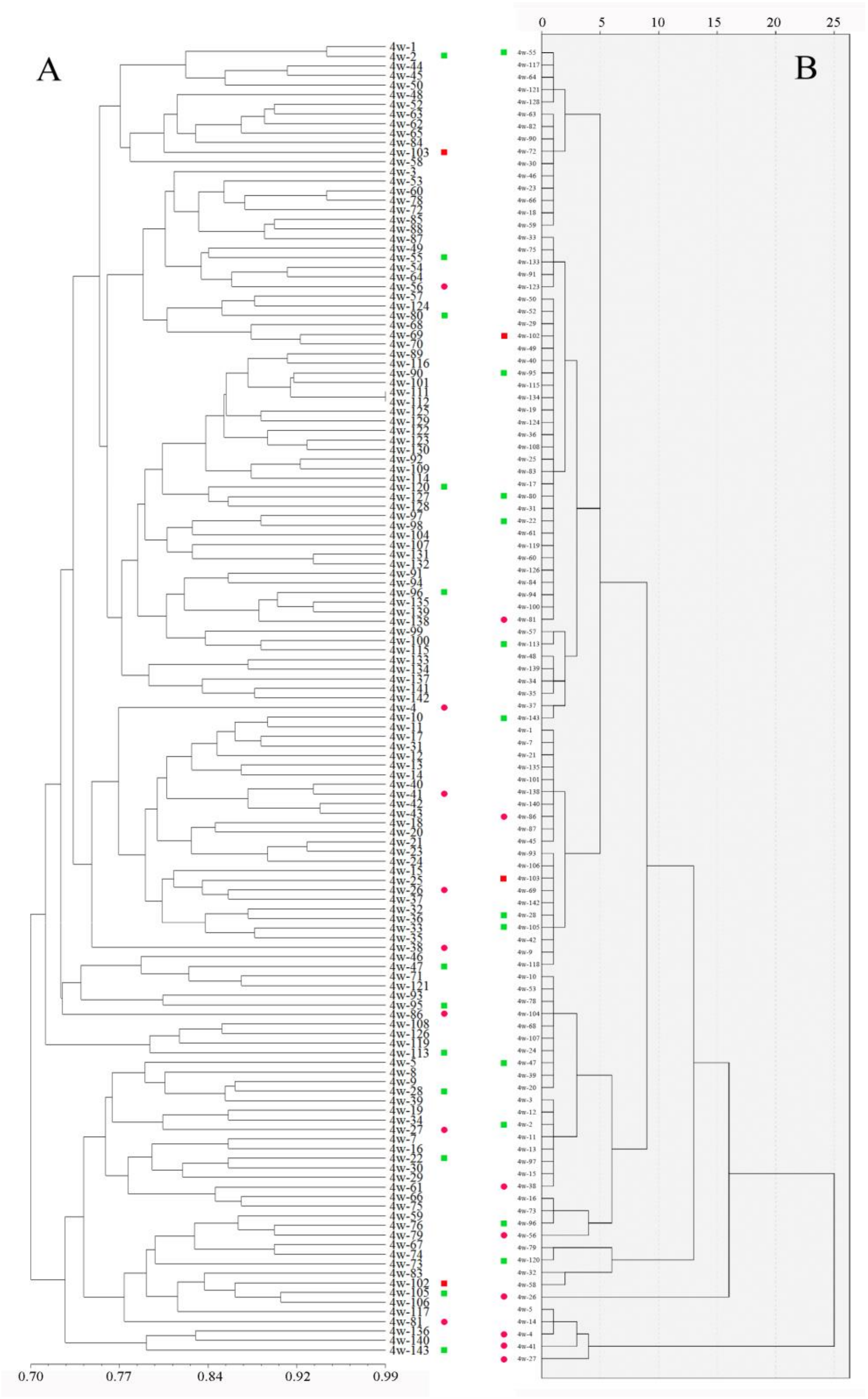

Figure L. Dendrograms denoting UPGMA clustering analyses of Dongxiang common wild rice accessions based on phenotypic and genotypic data.

A and B represent the dendrograms based on phenotypic and genotypic data, respectively. Red dots show accessions selected for the core collection with high genetic distance and green squares denote accessions selected for the core collection with a gradual increase in genetic distance (from low to high). Two non-shattering accessions (4w-102 and 4w-103) were selected as the core collections directly as shown by red squares. Numbers in vertical rows represent accessions. Numbers on the bottom of panel A represent genetic relevance and number on the top of panel B represent genetic distance.

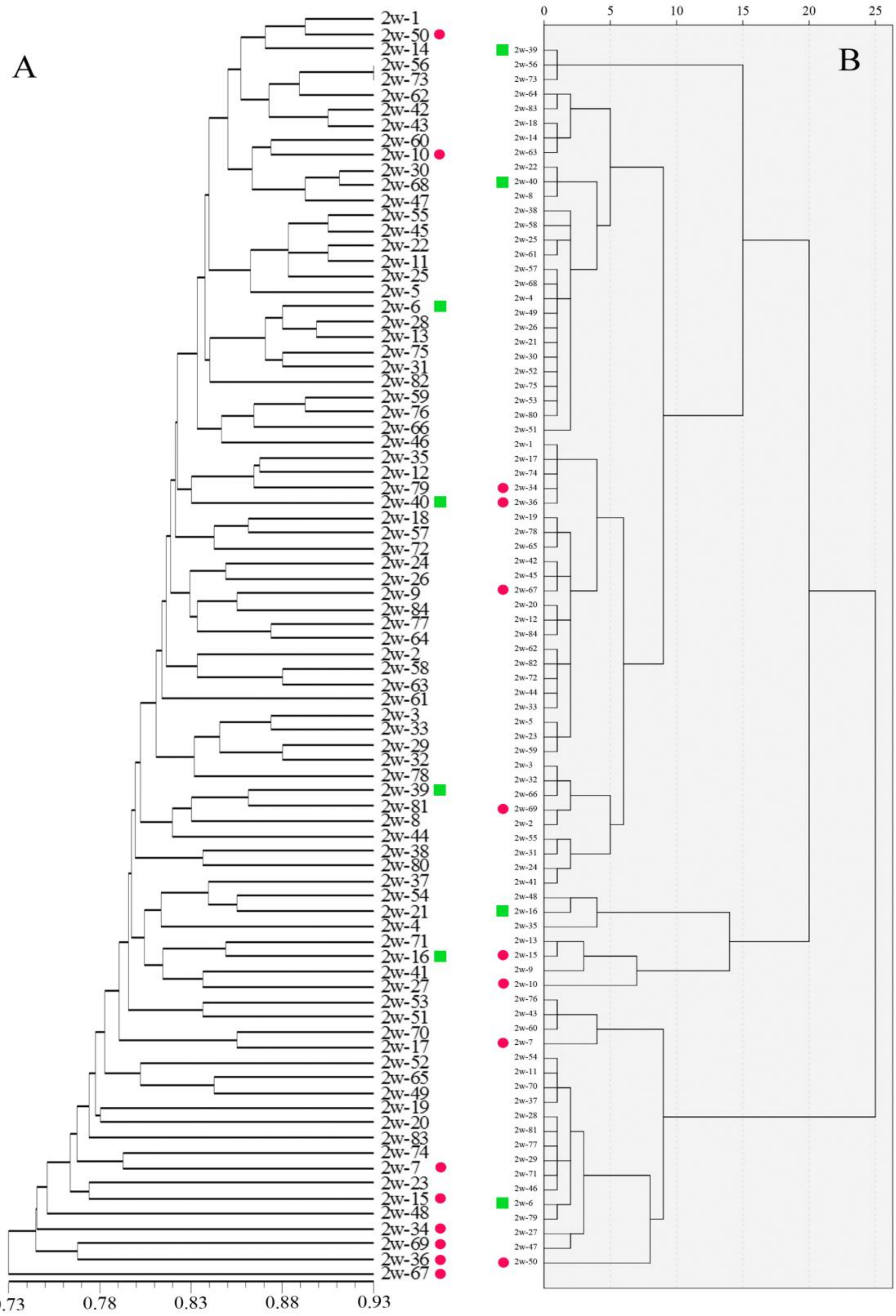

Figure M. Dendrograms denoting UPGMA clustering analyses of Boluo common wild rice accessions based on phenotypic and genotypic data.

A and B represent the dendrograms based on phenotypic and genotypic data, respectively. Red dots show accessions selected for the core collection with high genetic distance and green squares denote accessions selected for the core collection with a gradual increase in genetic distance (from low to high). Numbers in vertical rows represent accessions. Numbers on the bottom of panel A represent genetic relevance and number on the top of panel B represent genetic distance.

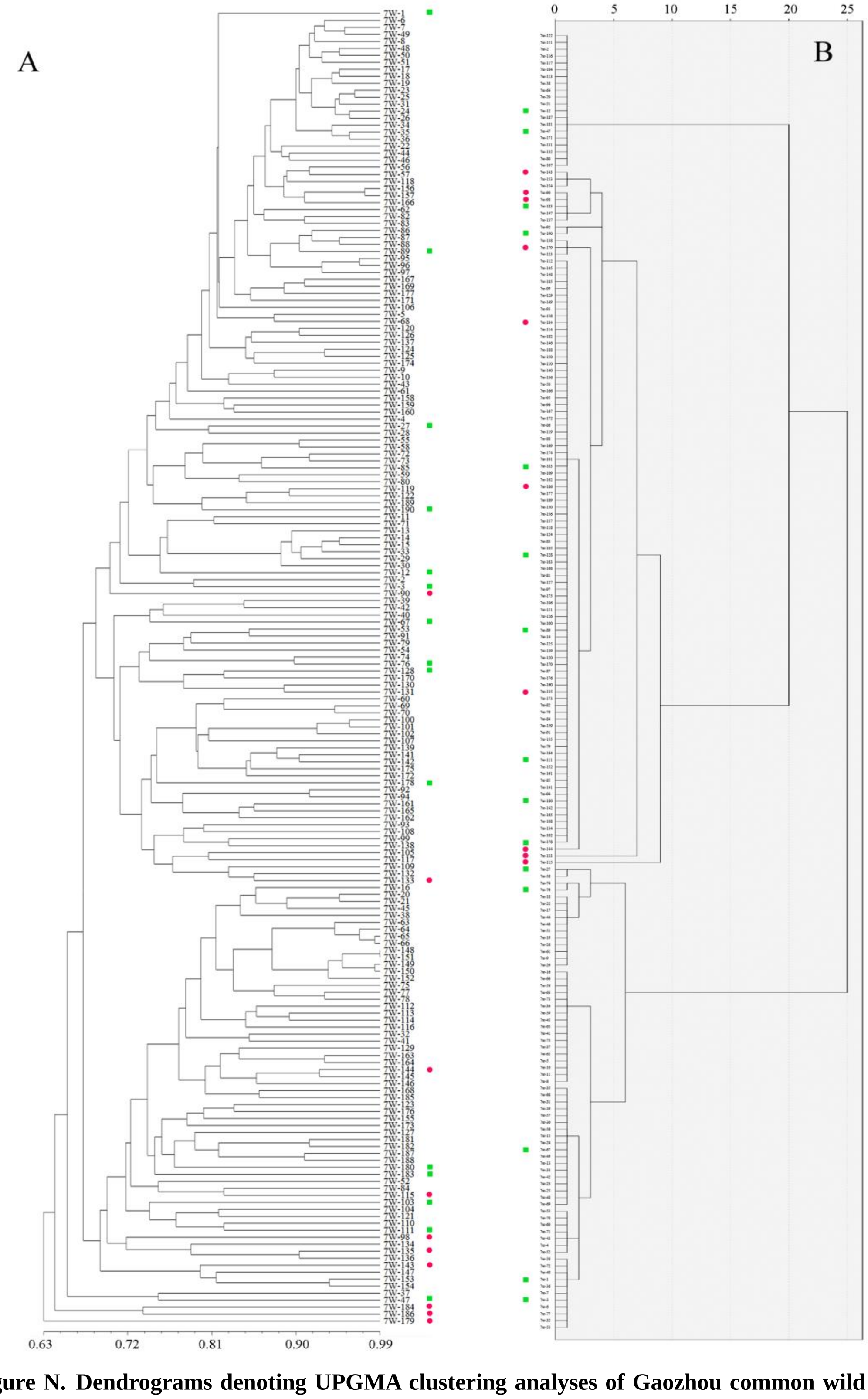

Figure N. Dendrograms denoting UPGMA clustering analyses of Gaozhou common wild rice accessions based on phenotypic and genotypic data.

A and B represent the dendrograms based on phenotypic and genotypic data, respectively. Red dots show accessions selected for the core collection with high genetic distance and green squares denote accessions selected for the core collection with a gradual increase in genetic distance (from low to high). Numbers in vertical rows represent accessions. Numbers on the bottom of panel A represent genetic relevance and number on the top of panel B represent genetic distance.

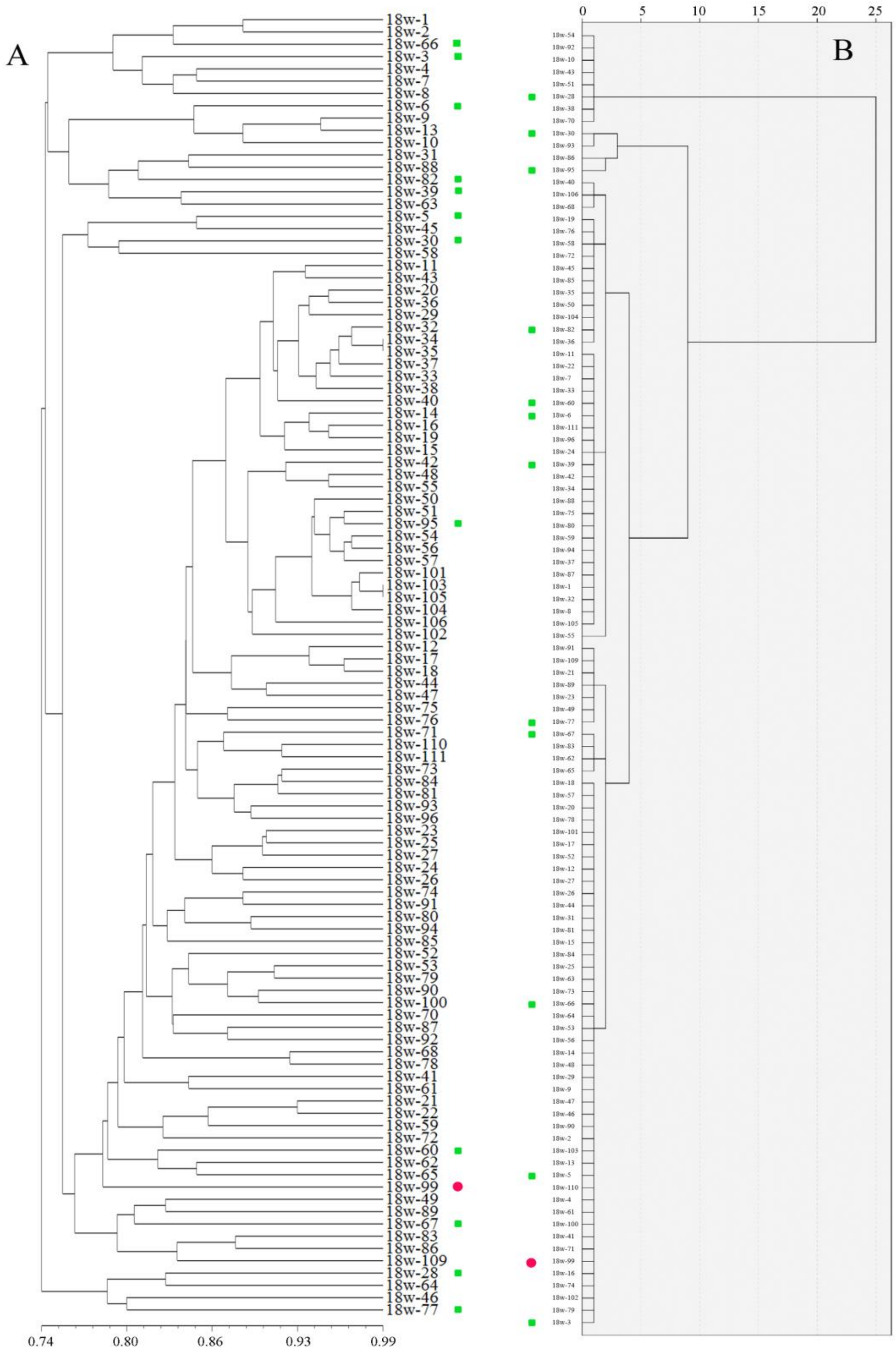

Figure O. Dendrograms denoting UPGMA clustering analyses of Huilai common wild rice accessions based on phenotypic and genotypic data.

A and B represent the dendrograms based on phenotypic and genotypic data, respectively. Red dots show accessions selected for the core collection with high genetic distance and green squares denote accessions selected for the core collection with a gradual increase in genetic distance (from low to high). Numbers in vertical rows represent accessions. Numbers at the bottom of A represent genetic relevance and numbers at the top of B represent genetic distance.

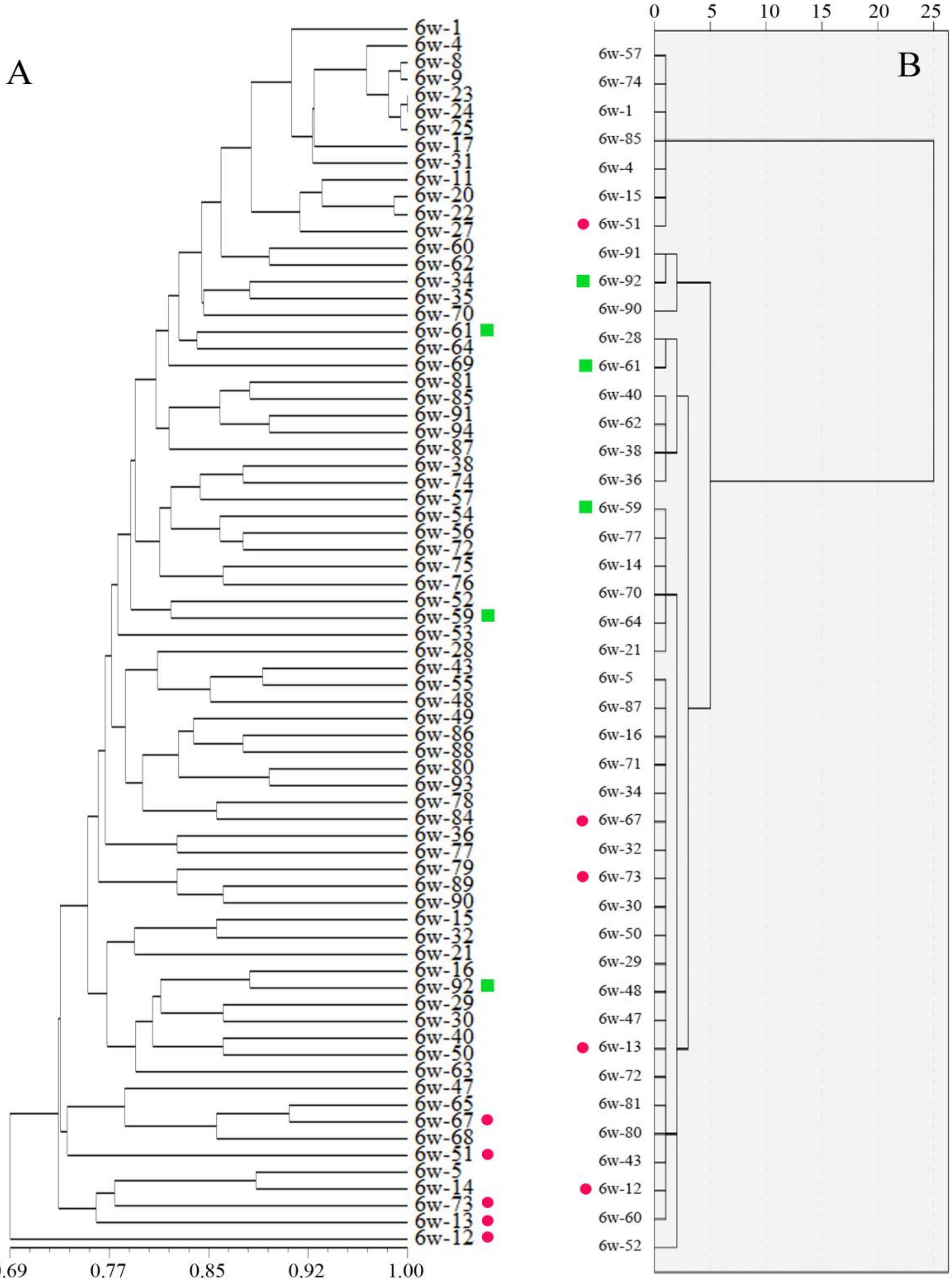

Figure P. Dendrograms denoting UPGMA clustering analyses of Fogang common wild rice accessions based on phenotypic and genotypic data.

A and B represent the dendrograms based on phenotypic and genotypic data, respectively. Red dots show accessions selected for the core collection with high genetic distance and green squares denote accessions selected for the core collection with a gradual increase in genetic distance (from low to high). Numbers in vertical rows represent accessions. Numbers at the bottom of A represent genetic relevance and numbers at the top of B represent genetic distance.

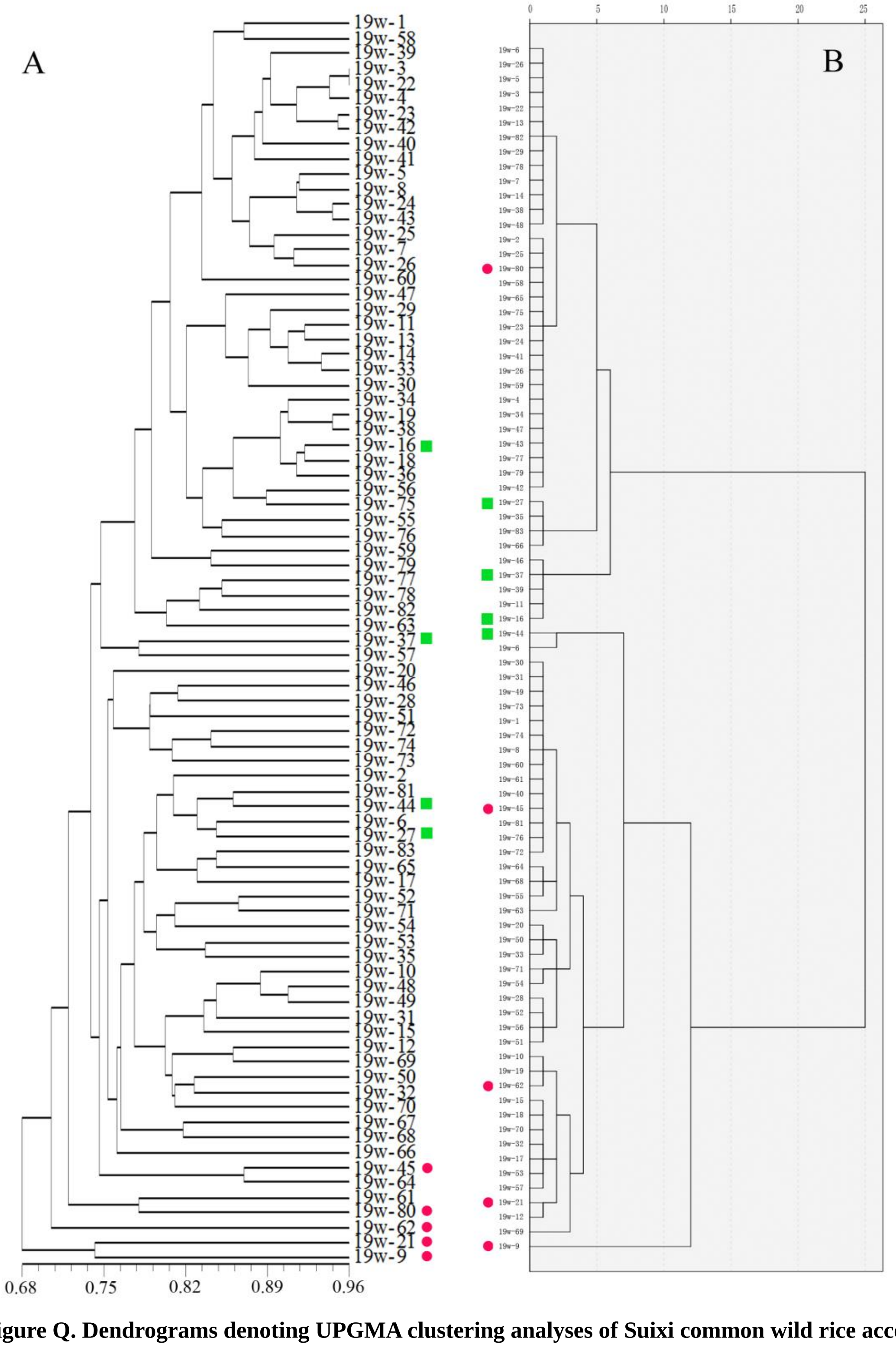

Figure Q. Dendrograms denoting UPGMA clustering analyses of Suixi common wild rice accessions based on phenotypic and genotypic data.

A and B represent the dendrograms based on phenotypic and genotypic data, respectively. Red dots accessions selected for the core collection with high genetic distance and green squares denote accessions selected for the core collection with a gradual increase in genetic distance (from low to high). Numbers in vertical rows represent accessions. Numbers at the bottom of A represent genetic relevance and numbers at the top of B represent genetic distance.

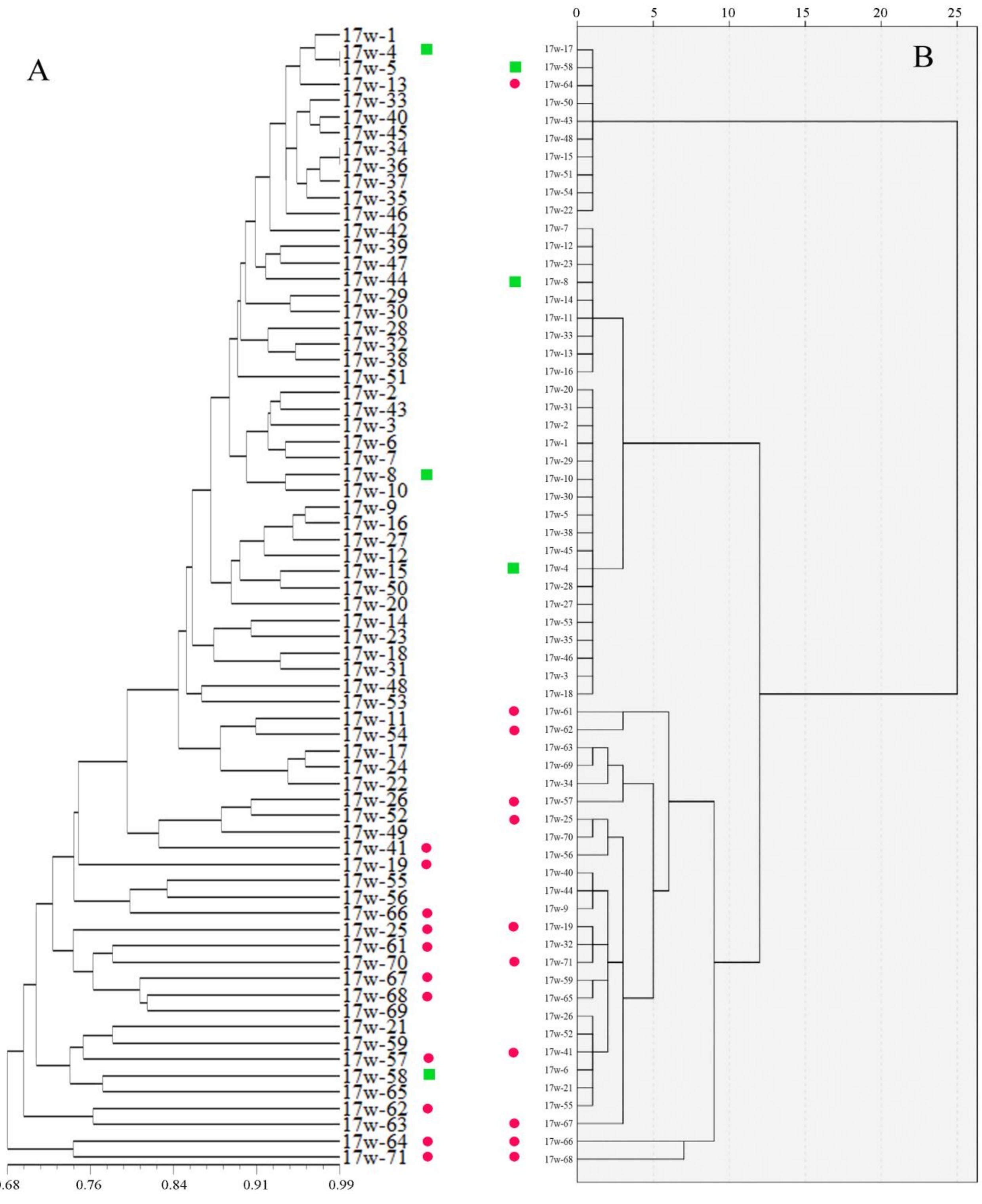

Figure R. Dendrograms denoting UPGMA clustering analyses of Qionghai common wild rice accessions based on phenotypic and genotypic data.

A and B represent the dendrograms based on phenotypic and genotypic data, respectively. Red dots show accessions selected for the core collection with high genetic distance and green squares denote accessions selected for the core collection with a gradual increase in genetic distance (from low to high). Numbers in vertical rows represent accessions. Numbers at the bottom of A represent genetic relevance and numbers at the top of B represent genetic distance.

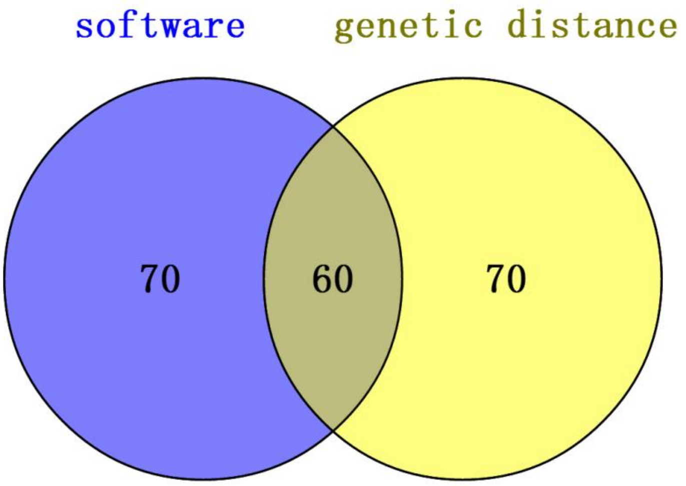

Figure S. Venn diagram representing the overlapping of core collections developed by QGA software and genetic distance.

Overlapping accessions identified using both QGA software (purple) and genetic distance data (yellow).

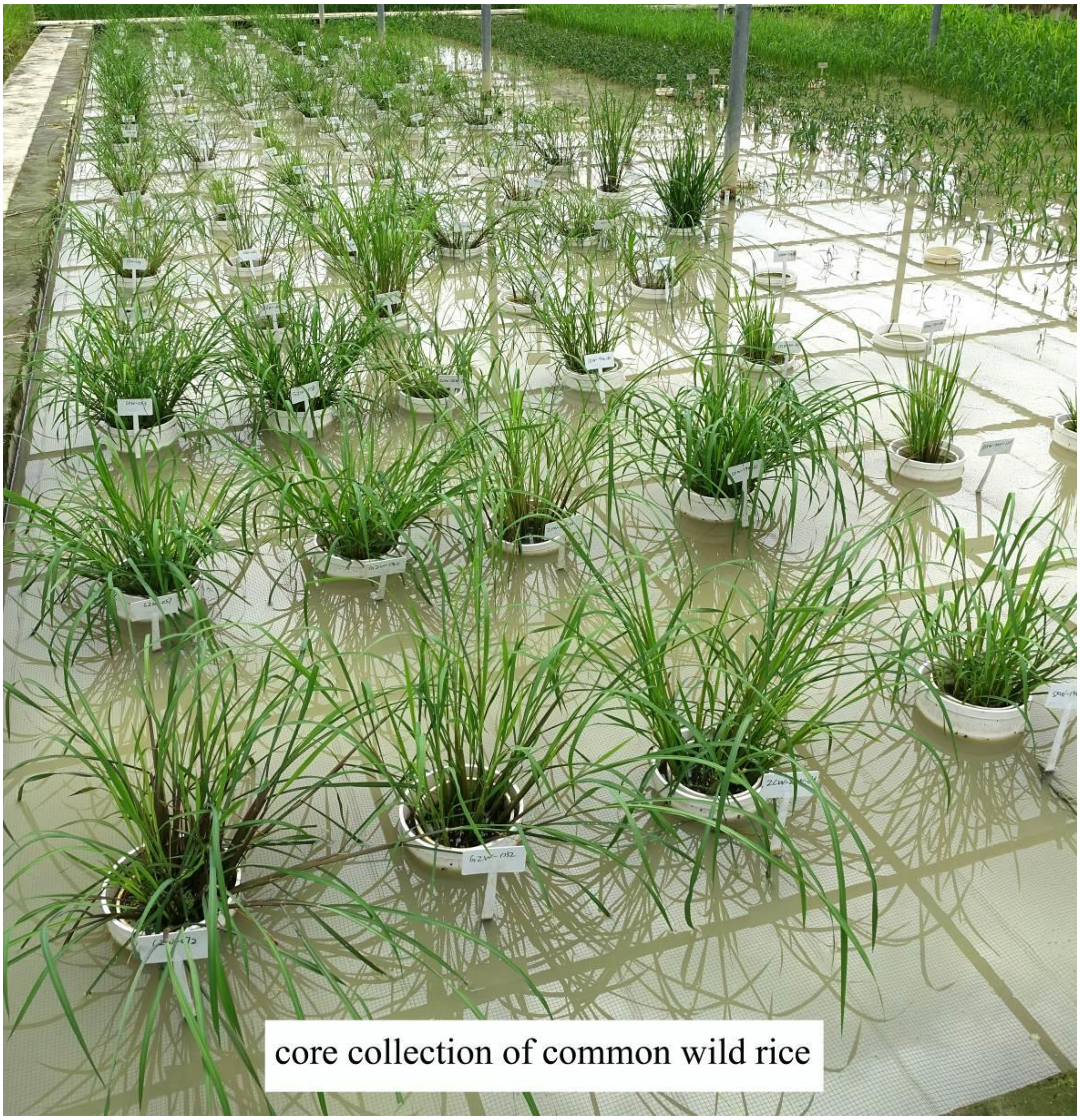

Figure T. Conservation of the core collection of common wild rice

All the core collections are kept under complete isolation from other accessions and cultivated rice.
