# Supplementary material for: Evaluation of Genetic Diversity and Development of a Core Collection of Wild Rice (Oryza rufipogon Griff.) Populations in China
Source: PLoS One. 2015 Dec 31;10(12):e0145990. doi: 10.1371/journal.pone.0145990 (PMC4703137; doi:10.1371/journal.pone.0145990)
Supplement: S1 Table — (DOCX) [file pone.0145990.s002.docx]

**S1 Table. Sampling information of common wild rice in China.**

| Populations | Groups | Sampling site | Ecotype of sampling site |
| --- | --- | --- | --- |
| Gaozhou | Daling village | Yakeng, Daling village, Zhenjiang town, Gaozhou city, Guangdong province | Wild rice is present in shallow old river that looks like a marsh. |
|  | Beitou village | Beitou village, Pengshan precinct, Zhenjiang town, Gaozhou city, Guangdong province | Wild rice is present on both sides of the river in deep water. |
|  | Libei village | Libei village, Fucun precinct, Gaozhou city, Guangdong province | The site is surrounded by mountains on three sides and a barrage on the fourth side. Wild rice is present in a deep marsh surrounded by a wall. We sampled the plants 5-6 meters from the barrage. |
|  | Xiangshandong | Xiangshandong, Xiangshan precinct, Xiangshan town, Gaozhou city, Guangdong province | Wild rice is present in marshes on both sides of the river. |
|  | Shuikudi | Shuikudi, Boshui village, Fushi precinct, Zhenjiang town, Gaozhou city, Guangdong province | There is a shallow water reservoir and wild rice is present in the shallow water of the reservoir. |
|  | Shanditan | Shanditan, Xiangshan precinct, Xiangshan town, Gaozhou city, Guangdong province | Wild rice is present along the river banks. |
| Dongxiang | Anjiashan | Anjiashan protection, Gangshangji town, Dongxiang county, Jiangxi province | The wild rice is located at the foot of a hill, and the hill is inside the natural conservation area. This group is near the reservoir and wild rice is present around the marsh, but isolated by a wall. |
|  | Zhangtang village | Zhangtang village, Gangshangji town, Dongxiang county, Jiangxi province | The wild rice is near fields of cultivated rice and sugarcane. There is a reservoir upstream and a pond downstream of the site. A stream flows past the site. |
|  | *Ex situ* garden | *Ex situ* garden of Dongxiang common wild rice, Institute of rice, Jiangxi Academy of Agricultural Sciences | This *ex situ* garden was established in the 1980s and consisted of 9 groups. We sampled 30 plants from this site. |
| Fogang | Shiguling | Shiguling, Banzhen village, Longshan town, Fogang county, Guangdong province | There is a water pool about 200 m up the side of the mountain. The pool is surrounded by wild plants. The wild rice grows around the pool and spans an area of about 70 m^2^. |
| Huilai | Houshan village | Houshan village, Xihou town, Huilai county, Jieyang city, Guangdong province | The site is near a fish pond and surrounded by mountains (cultivated rice was grown at this site before the 1960s). |
| Boluo | Xialangshankeng | Xialangshankeng, Xingxing village, Huzhen town, Boluo county, Huizhou city, Guangdong province | A pond surrounded by mountains. |
| Suixi | Neitang reservoir | Neitang reservoir, Suixi county, Zhanjiang city, Guangdong province | The site is surrounded by mountains. There is a water reservoir upstream of the site, and the wild rice is present at the base of a dam. |
| Zengcheng | Yangtian River | Yangtian River, Zengcheng city, Guangdong province | The wild rice grows on both banks of the river. |
| Qionghai | Fenglou village | Fenglou village, Zhongyuan town, Qionghai city, Hainan province | Wild rice exists on the hilly area. |
