# Supplementary material for: Evaluation of Genetic Diversity and Development of a Core Collection of Wild Rice (Oryza rufipogon Griff.) Populations in China
Source: PLoS One. 2015 Dec 31;10(12):e0145990. doi: 10.1371/journal.pone.0145990 (PMC4703137; doi:10.1371/journal.pone.0145990)
Supplement: S2 Table — (DOCX) [file pone.0145990.s003.docx]

**S2 Table. List of SSR primers used in the present study.**

| Chr NO. | Primer | Loci (cM) | Repeat type | Forward primer | Reverse primer |
| --- | --- | --- | --- | --- | --- |
| Chr1 | RM283 | 31.5 | AG | GGCATGAGAGTCTGTGATGTTGG | TAGTACTGCTCCATCTGCCTTGG |
|  | RM5 | 94.9 | AG | CACACTCCCATGCTAACAACTGG | CATCAAGAAGAGCAGTCCTGTGC |
|  | RM128 | 134.8 | AAG | TGATTTCTTGGAAGCGAAGAGTGAGG | CCTCCTTGTGCTCAGCCATGC |
| Chr2 | RM236 | 14.4 | AG | GTGAAGCACATGTGGCTAGTTGC | TTCCCTCAAGAATCTGTGTCTTCC |
|  | RM530 | 170.1 | AG | TTCTTTATTCCCTCGCACTGACC | CAATGATGCCACAAACCGTAACC |
|  | RM498 | 194.6 | AC | AATGACGTCTAAGGTGCTTTCAGC | CTGGGCTTAGTTCTTGACAGTGG |
| Chr3 | RM175 | 23.9 | CCG | GACGGAGGAGTTCGAGAGGAAGC | GTGAAGCGACTAGGCGGAGAAGG |
|  | RM135 | 157.3 | CCG | TCCATGCTCTTCAGCTTCTGG | GCTTCTACTGGAGGAGAGCAGAGG |
|  | RM130 | 208.2 | AG | CGAGAAGAAGGAGCCCATCTCG | GCGAAGCGAAGAGGAGCAAGG |
| Chr4 | RM518 | 25.5 | AG | AAGACACAAGCAAACAGCTCAACC | AAGCTTGCTTGGTTCAAGAGAGG |
|  | RM255 | 135.4 | AG | GAGGAGGAGGAGGAGAGATCAGG | AACGAAACCGCTCAGTTCAACC |
|  | RM559 | 155.8 | AAAC | AGAGCGATGGGTGTCAGTTTGC | CGTACGTACACTTGGCCCTATGC |
| Chr5 | RM413 | 26.7 | AG | CCAATCTTGTCTTCCGGATCTTGC | AGATAGCCATGGGCGATTCTTGG |
|  | RM31 | 118.8 | AG | CGCTCCTCCACTCTTCTCCTACC | CGTGCAGAAAGTCCATTACTCTCC |
|  | RM538 | 132.7 | AG | CAGTTTGACACAAGCAGCAAGC | CTCCACAGCTTGAGGGAATGC |
| Chr6 | RM204 | 25.1 | AG | CTAGCTAGCCATGCTCTCGTACC | CTGTGACTGACTTGGTCATAGGG |
|  | RM162 | 108.3 | AC | TTGTTCCAGTTCAGGTCTTGTGC | CCCTACAAACACCATAAGAAGCAACC |
|  | RM400 | 134.5 | AAT | TTACACCAGGCTACCCAAACTCG | TTGCTGAGTTCCCTCGTCTATCC |
| Chr7 | RM180 | 30.1 | AAT | CCTTCTCCTTCTTTCAGCTTCTGC | CAACTTGCTCTACTTGTGGTGAGG |
|  | RM429 | 96.9 | AC | TGAAGCCAAACTCAGTGTTTACCC | CCTCCAGCAATGTCTTTCTTATGC |
|  | RM248 | 116.6 | AG | AGAGAGCAAGTTTGAAGCGAAGC | ACCAAGAGGGTAGCCTAGCATGG |
| Chr8 | RM407 | 5.7 | AG | GACTACGAGACGAGTGATTTGAACC | GCGTGGGAAATGACTAGGAGTAGG |
|  | RM80 | 103.7 | AAG | CTCATCTCCGCCCTTGATTCC | GCCCATCAACCTCGTCTTCACC |
|  | RM447 | 124.6 | AAG | ACGGGCTTCTTCTCCTTCTCTCC | TCCCTTGTGCTGTCTCCTCTCC |
| Chr9 | RM444 | 3.3 | AT | TGCATCTTTCACCGTAGTCCTAGC | CTTGCTGGAGCTCGTAGATGC |
|  | RM201 | 81.2 | AG | GTACTCTCGCCGTTCACAACTCC | TTAGTGACCGGGATGACACAGC |
|  | RM205 | 114.7 | AG | CCTAAGAGGAGCCATCTAACAACTGG | CTTGGATATACTGGCCCTTCACG |
| Chr10 | RM216 | 17.6 | AG | GATGGTAAAGGAAGAACGTGTGC | CACTCATAGACGCATCACATAGCC |
|  | RM333 | 110.4 | AAT | GATGTACTTGCCAACATGCTCTCC | AGCACACGCGAGTGATGTAACG |
|  | RM591 | 118.3 | AC | CTCATAGGTGGGTTAGTTTCTTGG | GCTGGTTTACAACTTGCTACTCTACC |
| Chr11 | RM167 | 20.3 | AG | CTCCGAGTCCGACCACAAGG | TCCAGCCCTTCCTATCATATTGC |
|  | RM206 | 102.9 | AG | ATCGATCCGTATGGGTTCTAGC | GTCCATGTAGCCAATCTTATGTGG |
|  | RM144 | 123.2 | AAT | CATGTTGTGCTTGTCCTACTGC | AGCTAGAGGAGATCAGATGGTAGTGC |
| Chr12 | RM20A | 3.2 | AAT | TGTATGCACAGCTGCTCTACTCC | GCACGACCAGAAATTAACAAGG |
|  | RM235 | 91.3 | AG | AAGCTAGGGCTAACGAACGAACG | TCTCCATCTCCATCTCCATCTCC |
|  | RM17 | 109.1 | AG | GGAGAAAGAGAGGTGATCCTTTCC | CATGTCTTGGTGAGTGATGTTGC |

Chr represents chromosome number
