# Supplementary material for: Evaluation of Genetic Diversity and Development of a Core Collection of Wild Rice (Oryza rufipogon Griff.) Populations in China
Source: PLoS One. 2015 Dec 31;10(12):e0145990. doi: 10.1371/journal.pone.0145990 (PMC4703137; doi:10.1371/journal.pone.0145990)
Supplement: S3 Table — (DOCX) [file pone.0145990.s004.docx]

**S3 Table. Analysis of variance (ANOVA) of ten morphological traits from eight populations**

| **Traits** | **Population** | **Sum of squares** | **df** | **Mean square** | **F** |
| --- | --- | --- | --- | --- | --- |
| Panicle length (cm) | Among populations | 3013.63 | 7 | 430.52 | 8.55 |
|  | deviation | 2444.45 | 6 | 407.41 | 8.09 |
|  | Within population | 40870.90 | 878 | 50.33 |  |
|  | Total | 43884.53 | 885 |  |  |
| Secondary branches | Among populations | 518.24 | 7 | 74.04 | 16.96 |
|  | deviation | 469.00 | 6 | 78.17 | 17.91 |
|  | Within population | 3544.03 | 878 | 4.37 |  |
|  | Total | 4062.27 | 885 |  |  |
| Awn length (mm) | Among populations | 82684.25 | 7 | 11812.04 | 20.96 |
|  | deviation | 77415.01 | 6 | 12902.50 | 22.89 |
|  | Within population | 457620.33 | 878 | 563.57 |  |
|  | Total | 540304.57 | 885 |  |  |
| Number of spikelets per panicle | Among populations | 54132.66 | 7 | 7733.24 | 23.83 |
|  | deviation | 51128.50 | 6 | 8521.42 | 26.26 |
|  | Within population | 263470.03 | 878 | 324.47 |  |
|  | Total | 317602.68 | 885 |  |  |
| Filled spikelets per panicle | Among populations | 59451.21 | 7 | 8493.03 | 62.36 |
|  | deviation | 46154.22 | 6 | 7692.37 | 56.48 |
|  | Within population | 110594.78 | 878 | 136.20 |  |
|  | Total | 170045.99 | 885 |  |  |
| Seed setting (%) | Among populations | 15.89 | 7 | 2.27 | 44.36 |
|  | deviation | 13.95 | 6 | 2.33 | 45.44 |
|  | Within population | 41.54 | 878 | 0.05 |  |
|  | Total | 57.43 | 885 |  |  |
| Spikelet length | Among populations | 674431 | 7 | 96347.30 | 140.78 |
|  | deviation | 575493.44 | 6 | 95916 | 140.14 |
|  | Within population | 555055.42 | 878 | 684 |  |
|  | Total | 1229486.50 | 885 |  |  |
| Spikelet width | Among populations | 56322 | 7 | 8046.00 | 132.01 |
|  | deviation | 47584.10 | 6 | 7931 | 130.12 |
|  | Within population | 49430.75 | 878 | 61 |  |
|  | Total | 105752.78 | 885 |  |  |
| Spikelet length-width ratio | Among populations | 357.59 | 7 | 51.08 | 33.75 |
|  | deviation | 253.30 | 6 | 42.22 | 27.89 |
|  | Within population | 1229.13 | 878 | 1.51 |  |
|  | Total | 1586.72 | 885 |  |  |
| Plant height (cm) | Among populations | 18390.46 | 7 | 2627.21 | 2.50 |
|  | deviation | 16821.15 | 6 | 2803.53 | 2.67 |
|  | Within population | 854313.70 | 878 | 1052.11 |  |
|  | Total | 872704.16 | 885 |  |  |

df represent the degrees of freedom; F represents mean square in the group.

All the traits showed significant difference (p<0.05) among populations
