# Supplementary material for: Evaluation of Genetic Diversity and Development of a Core Collection of Wild Rice (Oryza rufipogon Griff.) Populations in China
Source: PLoS One. 2015 Dec 31;10(12):e0145990. doi: 10.1371/journal.pone.0145990 (PMC4703137; doi:10.1371/journal.pone.0145990)
Supplement: S4 Table — (DOCX) [file pone.0145990.s005.docx]

**S4 Table. The genetic diversity detected by 36 marker loci in eight populations.**

| Population | Chr NO. | Markers | Polymorphic alleles | Total alleles | PPB (%) | na | ne | h | I | PIC | |  |
| --- | --- | --- | --- | --- | --- | --- | --- | --- | --- | --- | --- | --- |
| Dongxiang (DX) | | chr1 | RM283 | 7 | 8 | 87.50 | 1.875 | 1.563 | 0.334 | 0.495 | 0.836 | |
|  |  | RM5 | 3 | 9 | 33.33 | 1.333 | 1.054 | 0.046 | 0.086 | 0.835 | |  |
|  |  | RM128 | 5 | 6 | 83.33 | 1.833 | 1.121 | 0.102 | 0.198 | 0.564 | |  |
|  | chr2 | RM236 | 2 | 3 | 66.67 | 1.667 | 1.548 | 0.301 | 0.429 | 0.568 | |  |
|  |  | RM530 | 3 | 4 | 75.00 | 1.750 | 1.338 | 0.212 | 0.329 | 0.580 | |  |
|  |  | RM498 | 2 | 3 | 66.67 | 1.667 | 1.651 | 0.329 | 0.458 | 0.585 | |  |
|  | chr3 | RM175 | 0 | 3 | 0.00 | 1.000 | 1.000 | 0.000 | 0.000 | 0.000 | |  |
|  |  | RM135 | 2 | 3 | 66.67 | 1.667 | 1.603 | 0.316 | 0.445 | 0.576 | |  |
|  |  | RM130 | 3 | 3 | 100.00 | 2.000 | 1.344 | 0.251 | 0.415 | 0.475 | |  |
|  | chr4 | RM518 | 4 | 4 | 100.00 | 2.000 | 1.424 | 0.278 | 0.435 | 0.563 | |  |
|  |  | RM255 | 8 | 9 | 88.89 | 1.889 | 1.667 | 0.368 | 0.533 | 0.860 | |  |
|  |  | RM559 | 1 | 2 | 50.00 | 1.500 | 1.095 | 0.080 | 0.149 | 0.216 | |  |
|  | chr5 | RM413 | 3 | 3 | 100.00 | 2.000 | 1.670 | 0.392 | 0.579 | 0.550 | |  |
|  |  | RM31 | 3 | 3 | 100.00 | 2.000 | 1.411 | 0.263 | 0.420 | 0.459 | |  |
|  |  | RM538 | 5 | 5 | 100.00 | 2.000 | 1.533 | 0.302 | 0.452 | 0.642 | |  |
|  | chr6 | RM204 | 5 | 6 | 83.33 | 1.833 | 1.553 | 0.327 | 0.483 | 0.782 | |  |
|  |  | RM162 | 5 | 6 | 83.33 | 1.833 | 1.470 | 0.286 | 0.434 | 0.758 | |  |
|  |  | RM400 | 5 | 5 | 100.00 | 2.000 | 1.750 | 0.419 | 0.607 | 0.745 | |  |
|  | chr7 | RM180 | 5 | 6 | 83.33 | 1.833 | 1.392 | 0.243 | 0.373 | 0.757 | |  |
|  |  | RM429 | 3 | 4 | 75.00 | 1.750 | 1.536 | 0.304 | 0.444 | 0.662 | |  |
|  |  | RM248 | 2 | 3 | 66.67 | 1.667 | 1.456 | 0.270 | 0.396 | 0.569 | |  |
|  | chr8 | RM407 | 2 | 3 | 66.67 | 1.667 | 1.558 | 0.304 | 0.432 | 0.577 | |  |
|  |  | RM80 | 5 | 7 | 71.43 | 1.714 | 1.380 | 0.238 | 0.365 | 0.793 | |  |
|  |  | RM447 | 7 | 8 | 87.50 | 1.875 | 1.202 | 0.131 | 0.220 | 0.661 | |  |
|  | chr9 | RM444 | 2 | 2 | 100.00 | 2.000 | 1.746 | 0.417 | 0.605 | 0.354 | |  |
|  |  | RM201 | 0 | 3 | 0.00 | 1.000 | 1.000 | 0.000 | 0.000 | 0.000 | |  |
|  |  | RM205 | 8 | 8 | 100.00 | 2.000 | 1.494 | 0.293 | 0.448 | 0.784 | |  |
|  | chr10 | RM216 | 4 | 6 | 66.67 | 1.667 | 1.161 | 0.106 | 0.172 | 0.637 | |  |
|  |  | RM333 | 5 | 5 | 100.00 | 2.000 | 1.331 | 0.228 | 0.378 | 0.690 | |  |
|  |  | RM591 | 1 | 4 | 25.00 | 1.250 | 1.030 | 0.027 | 0.054 | 0.624 | |  |
|  | chr11 | RM167 | 11 | 11 | 100.00 | 2.000 | 1.588 | 0.332 | 0.496 | 0.862 | |  |
|  |  | RM206 | 7 | 7 | 100.00 | 2.000 | 1.753 | 0.409 | 0.592 | 0.814 | |  |
|  |  | RM144 | 4 | 4 | 100.00 | 2.000 | 1.616 | 0.364 | 0.545 | 0.664 | |  |
|  | chr12 | RM20A | 10 | 10 | 100.00 | 2.000 | 1.288 | 0.194 | 0.321 | 0.761 | |  |
|  |  | RM235 | 3 | 4 | 75.00 | 1.750 | 1.514 | 0.299 | 0.439 | 0.668 | |  |
|  |  | RM17 | 6 | 6 | 100.00 | 2.000 | 1.695 | 0.398 | 0.584 | 0.793 | |  |
|  |  | Means | 4.194 | 5.167 | 81.18 | 1.778 | 1.431 | 0.254 | 0.384 | 0.618 | |  |
|  |  | St.Dev | 2.558 | 2.339 | 0.268 | 0.268 | 0.222 | 0.118 | 0.167 | 0.205 | |  |
| Boluo (BL) | chr1 | RM283 | 7 | 8 | 87.50 | 1.857 | 1.691 | 0.367 | 0.524 | 0.807 | |  |
|  |  | RM5 | 3 | 6 | 50.00 | 1.500 | 1.356 | 0.202 | 0.296 | 0.793 | |  |
|  |  | RM128 | 4 | 5 | 80.00 | 1.800 | 1.337 | 0.212 | 0.334 | 0.624 | |  |
|  | chr2 | RM236 | 3 | 6 | 50.00 | 1.500 | 1.097 | 0.079 | 0.144 | 0.669 | |  |
|  |  | RM530 | 7 | 7 | 100.00 | 2.000 | 1.351 | 0.217 | 0.351 | 0.712 | |  |
|  |  | RM498 | 1 | 3 | 33.33 | 1.333 | 1.312 | 0.161 | 0.225 | 0.590 | |  |
|  | chr3 | RM175 | 1 | 2 | 50.00 | 1.500 | 1.500 | 0.250 | 0.347 | 0.367 | |  |
|  |  | RM135 | 2 | 4 | 50.00 | 1.500 | 1.500 | 0.250 | 0.347 | 0.692 | |  |
|  |  | RM130 | 3 | 4 | 75.00 | 1.611 | 1.521 | 0.268 | 0.379 | 0.681 | |  |
|  | chr4 | RM518 | 5 | 6 | 83.33 | 1.600 | 1.260 | 0.175 | 0.278 | 0.704 | |  |
|  |  | RM255 | 0 | 1 | 0.00 | 1.000 | 1.000 | 0.000 | 0.000 | 0.000 | |  |
|  |  | RM559 | 5 | 8 | 62.50 | 1.625 | 1.272 | 0.153 | 0.236 | 0.766 | |  |
|  | chr5 | RM413 | 4 | 4 | 100.00 | 2.000 | 1.545 | 0.328 | 0.495 | 0.604 | |  |
|  |  | RM31 | 2 | 3 | 66.67 | 1.667 | 1.465 | 0.274 | 0.401 | 0.592 | |  |
|  |  | RM538 | 3 | 4 | 75.00 | 1.750 | 1.227 | 0.174 | 0.295 | 0.370 | |  |
|  | chr6 | RM204 | 2 | 4 | 50.00 | 1.500 | 1.427 | 0.229 | 0.324 | 0.685 | |  |
|  |  | RM162 | 3 | 4 | 75.00 | 1.750 | 1.446 | 0.248 | 0.368 | 0.611 | |  |
|  |  | RM400 | 3 | 3 | 100.00 | 2.000 | 1.607 | 0.351 | 0.524 | 0.447 | |  |
|  | chr7 | RM180 | 7 | 7 | 100.00 | 1.833 | 1.439 | 0.269 | 0.409 | 0.737 | |  |
|  |  | RM429 | 2 | 3 | 66.67 | 1.667 | 1.341 | 0.188 | 0.284 | 0.496 | |  |
|  |  | RM248 | 3 | 5 | 60.00 | 1.600 | 1.365 | 0.227 | 0.339 | 0.724 | |  |
|  | chr8 | RM407 | 2 | 4 | 50.00 | 1.500 | 1.222 | 0.142 | 0.224 | 0.600 | |  |
|  |  | RM80 | 4 | 5 | 80.00 | 1.800 | 1.409 | 0.226 | 0.341 | 0.667 | |  |
|  |  | RM447 | 4 | 7 | 57.14 | 1.571 | 1.191 | 0.142 | 0.237 | 0.749 | |  |
|  | chr9 | RM444 | 3 | 4 | 75.00 | 1.750 | 1.457 | 0.283 | 0.424 | 0.697 | |  |
|  |  | RM201 | 0 | 1 | 0.00 | 1.000 | 1.000 | 0.000 | 0.000 | 0.000 | |  |
|  |  | RM205 | 0 | 1 | 0.00 | 1.000 | 1.000 | 0.000 | 0.000 | 0.000 | |  |
|  | chr10 | RM216 | 3 | 3 | 100.00 | 2.000 | 1.963 | 0.490 | 0.683 | 0.590 | |  |
|  |  | RM333 | 2 | 2 | 100.00 | 2.000 | 1.573 | 0.345 | 0.522 | 0.439 | |  |
|  |  | RM591 | 3 | 3 | 100.00 | 2.000 | 1.776 | 0.435 | 0.626 | 0.608 | |  |
|  | chr11 | RM167 | 7 | 8 | 87.50 | 1.875 | 1.524 | 0.314 | 0.472 | 0.817 | |  |
|  |  | RM206 | 2 | 3 | 66.67 | 1.667 | 1.544 | 0.298 | 0.426 | 0.579 | |  |
|  |  | RM144 | 5 | 5 | 100.00 | 2.000 | 1.876 | 0.466 | 0.658 | 0.764 | |  |
|  | chr12 | RM20A | 3 | 3 | 100.00 | 2.000 | 1.693 | 0.399 | 0.586 | 0.504 | |  |
|  |  | RM235 | 3 | 3 | 100.00 | 1.667 | 1.424 | 0.252 | 0.376 | 0.497 | |  |
|  |  | RM17 | 3 | 3 | 100.00 | 2.000 | 1.829 | 0.452 | 0.644 | 0.574 | |  |
|  |  | Means | 3.167 | 4.222 | 75.00 | 1.678 | 1.432 | 0.246 | 0.364 | 0.577 | |  |
|  |  | St.Dev | 1.833 | 1.931 | 0.288 | 0.279 | 0.231 | 0.121 | 0.170 | 0.208 | |  |
| Zengcheng (ZC) | chr1 | RM283 | 5 | 6 | 83.33 | 1.833 | 1.417 | 0.244 | 0.369 | 0.717 | |  |
|  |  | RM5 | 5 | 6 | 83.33 | 1.833 | 1.306 | 0.194 | 0.308 | 0.629 | |  |
|  |  | RM128 | 7 | 7 | 100.00 | 2.000 | 1.376 | 0.254 | 0.409 | 0.756 | |  |
|  | chr2 | RM236 | 4 | 8 | 50.00 | 1.500 | 1.087 | 0.068 | 0.123 | 0.623 | |  |
|  |  | RM530 | 9 | 10 | 90.00 | 1.900 | 1.592 | 0.324 | 0.476 | 0.856 | |  |
|  |  | RM498 | 8 | 10 | 80.00 | 1.800 | 1.134 | 0.104 | 0.192 | 0.734 | |  |
|  | chr3 | RM175 | 5 | 6 | 83.33 | 1.833 | 1.277 | 0.167 | 0.265 | 0.639 | |  |
|  |  | RM135 | 4 | 7 | 57.14 | 1.571 | 1.295 | 0.173 | 0.261 | 0.812 | |  |
|  |  | RM130 | 10 | 10 | 100.00 | 2.000 | 1.605 | 0.349 | 0.523 | 0.858 | |  |
|  | chr4 | RM518 | 8 | 8 | 100.00 | 2.000 | 1.429 | 0.261 | 0.410 | 0.774 | |  |
|  |  | RM255 | 4 | 4 | 100.00 | 2.000 | 1.293 | 0.203 | 0.331 | 0.595 | |  |
|  |  | RM559 | 11 | 11 | 100.00 | 2.000 | 1.360 | 0.213 | 0.326 | 0.813 | |  |
|  | chr5 | RM413 | 7 | 7 | 100.00 | 2.000 | 1.487 | 0.274 | 0.410 | 0.728 | |  |
|  |  | RM31 | 8 | 9 | 88.89 | 1.889 | 1.497 | 0.304 | 0.462 | 0.846 | |  |
|  |  | RM538 | 13 | 14 | 92.86 | 1.929 | 1.386 | 0.230 | 0.358 | 0.863 | |  |
|  | chr6 | RM204 | 14 | 14 | 100.00 | 2.000 | 1.423 | 0.252 | 0.391 | 0.869 | |  |
|  |  | RM162 | 14 | 14 | 100.00 | 2.000 | 1.428 | 0.249 | 0.383 | 0.865 | |  |
|  |  | RM400 | 12 | 12 | 100.00 | 2.000 | 1.521 | 0.316 | 0.481 | 0.875 | |  |
|  | chr7 | RM180 | 11 | 11 | 100.00 | 2.000 | 1.339 | 0.209 | 0.335 | 0.803 | |  |
|  |  | RM429 | 9 | 10 | 90.00 | 1.900 | 1.377 | 0.235 | 0.360 | 0.827 | |  |
|  |  | RM248 | 9 | 10 | 90.00 | 1.900 | 1.469 | 0.287 | 0.439 | 0.849 | |  |
|  | chr8 | RM407 | 6 | 8 | 75.00 | 1.750 | 1.373 | 0.239 | 0.371 | 0.839 | |  |
|  |  | RM80 | 10 | 10 | 100.00 | 2.000 | 1.323 | 0.211 | 0.339 | 0.769 | |  |
|  |  | RM447 | 9 | 10 | 90.00 | 1.900 | 1.307 | 0.212 | 0.351 | 0.823 | |  |
|  | chr9 | RM444 | 7 | 8 | 87.50 | 1.875 | 1.590 | 0.339 | 0.497 | 0.833 | |  |
|  |  | RM201 | 5 | 5 | 100.00 | 2.000 | 1.503 | 0.287 | 0.437 | 0.626 | |  |
|  |  | RM205 | 9 | 9 | 100.00 | 2.000 | 1.429 | 0.270 | 0.420 | 0.795 | |  |
|  | chr10 | RM216 | 9 | 9 | 100.00 | 2.000 | 1.403 | 0.253 | 0.395 | 0.792 | |  |
|  |  | RM333 | 7 | 7 | 100.00 | 2.000 | 1.449 | 0.271 | 0.418 | 0.727 | |  |
|  |  | RM591 | 10 | 10 | 100.00 | 2.000 | 1.500 | 0.289 | 0.441 | 0.830 | |  |
|  | chr11 | RM167 | 8 | 10 | 80.00 | 1.800 | 1.500 | 0.293 | 0.437 | 0.865 | |  |
|  |  | RM206 | 5 | 6 | 83.33 | 1.833 | 1.387 | 0.257 | 0.402 | 0.746 | |  |
|  |  | RM144 | 9 | 9 | 100.00 | 2.000 | 1.377 | 0.216 | 0.327 | 0.740 | |  |
|  | chr12 | RM20A | 8 | 8 | 100.00 | 2.000 | 1.564 | 0.323 | 0.480 | 0.798 | |  |
|  |  | RM235 | 9 | 10 | 90.00 | 1.900 | 1.401 | 0.252 | 0.394 | 0.832 | |  |
|  |  | RM17 | 4 | 5 | 80.00 | 1.800 | 1.403 | 0.252 | 0.389 | 0.620 | |  |
|  |  | Means | 8.111 | 8.833 | 91.82 | 1.910 | 1.406 | 0.246 | 0.381 | 0.777 | |  |
|  |  | St.Dev | 2.726 | 2.444 | 0.120 | 0.120 | 0.112 | 0.059 | 0.081 | 0.082 | |  |
| Gaozhou (GZ) | chr1 | RM283 | 5 | 5 | 100.00 | 2.000 | 1.550 | 0.314 | 0.474 | 0.664 | |  |
|  |  | RM5 | 5 | 6 | 83.33 | 2.000 | 1.385 | 0.250 | 0.403 | 0.746 | |  |
|  |  | RM128 | 6 | 6 | 100.00 | 2.000 | 1.436 | 0.266 | 0.412 | 0.700 | |  |
|  | chr2 | RM236 | 7 | 8 | 87.50 | 1.875 | 1.436 | 0.271 | 0.418 | 0.824 | |  |
|  |  | RM530 | 5 | 5 | 100.00 | 2.000 | 1.664 | 0.383 | 0.565 | 0.724 | |  |
|  |  | RM498 | 4 | 4 | 100.00 | 2.000 | 1.563 | 0.327 | 0.487 | 0.595 | |  |
|  | chr3 | RM175 | 3 | 4 | 75.00 | 1.750 | 1.317 | 0.193 | 0.300 | 0.591 | |  |
|  |  | RM135 | 4 | 4 | 100.00 | 2.000 | 1.362 | 0.249 | 0.407 | 0.622 | |  |
|  |  | RM130 | 4 | 4 | 100.00 | 2.000 | 1.402 | 0.228 | 0.346 | 0.419 | |  |
|  | chr4 | RM518 | 5 | 5 | 100.00 | 2.000 | 1.453 | 0.274 | 0.424 | 0.647 | |  |
|  |  | RM255 | 5 | 5 | 100.00 | 2.000 | 1.871 | 0.458 | 0.649 | 0.753 | |  |
|  |  | RM559 | 1 | 2 | 50.00 | 1.500 | 1.493 | 0.248 | 0.345 | 0.368 | |  |
|  | chr5 | RM413 | 4 | 4 | 100.00 | 2.000 | 1.594 | 0.345 | 0.516 | 0.621 | |  |
|  |  | RM31 | 5 | 5 | 100.00 | 2.000 | 1.604 | 0.333 | 0.495 | 0.672 | |  |
|  |  | RM538 | 6 | 6 | 100.00 | 2.000 | 1.661 | 0.397 | 0.586 | 0.809 | |  |
|  | chr6 | RM204 | 5 | 5 | 100.00 | 2.000 | 1.748 | 0.402 | 0.581 | 0.721 | |  |
|  |  | RM162 | 7 | 7 | 100.00 | 2.000 | 1.624 | 0.363 | 0.535 | 0.803 | |  |
|  |  | RM400 | 5 | 5 | 100.00 | 2.000 | 1.394 | 0.253 | 0.402 | 0.670 | |  |
|  | chr7 | RM180 | 6 | 6 | 100.00 | 2.000 | 1.284 | 0.190 | 0.311 | 0.584 | |  |
|  |  | RM429 | 8 | 8 | 100.00 | 2.000 | 1.526 | 0.319 | 0.488 | 0.821 | |  |
|  |  | RM248 | 3 | 7 | 42.86 | 1.429 | 1.162 | 0.101 | 0.163 | 0.791 | |  |
|  | chr8 | RM407 | 1 | 6 | 16.67 | 1.167 | 1.137 | 0.075 | 0.107 | 0.804 | |  |
|  |  | RM80 | 6 | 6 | 100.00 | 2.000 | 1.441 | 0.296 | 0.468 | 0.789 | |  |
|  |  | RM447 | 6 | 6 | 100.00 | 2.000 | 1.296 | 0.221 | 0.377 | 0.688 | |  |
|  | chr9 | RM444 | 1 | 2 | 50.00 | 1.500 | 1.398 | 0.222 | 0.318 | 0.353 | |  |
|  |  | RM201 | 0 | 1 | 0.00 | 1.000 | 1.000 | 0.000 | 0.000 | 0.000 | |  |
|  |  | RM205 | 7 | 7 | 100.00 | 2.000 | 1.514 | 0.305 | 0.465 | 0.767 | |  |
|  | chr10 | RM216 | 8 | 8 | 100.00 | 2.000 | 1.501 | 0.298 | 0.456 | 0.804 | |  |
|  |  | RM333 | 5 | 5 | 100.00 | 2.000 | 1.568 | 0.310 | 0.459 | 0.647 | |  |
|  |  | RM591 | 2 | 3 | 66.67 | 1.667 | 1.523 | 0.293 | 0.421 | 0.592 | |  |
|  | chr11 | RM167 | 6 | 6 | 100.00 | 2.000 | 1.489 | 0.306 | 0.477 | 0.736 | |  |
|  |  | RM206 | 4 | 4 | 100.00 | 2.000 | 1.531 | 0.306 | 0.456 | 0.548 | |  |
|  |  | RM144 | 5 | 5 | 100.00 | 2.000 | 1.629 | 0.380 | 0.566 | 0.757 | |  |
|  | chr12 | RM20A | 6 | 6 | 100.00 | 2.000 | 1.684 | 0.396 | 0.581 | 0.794 | |  |
|  |  | RM235 | 4 | 4 | 100.00 | 2.000 | 1.361 | 0.259 | 0.423 | 0.637 | |  |
|  |  | RM17 | 5 | 5 | 100.00 | 2.000 | 1.553 | 0.327 | 0.490 | 0.679 | |  |
|  |  | Means | 4.694 | 5.139 | 91.35 | 1.886 | 1.476 | 0.282 | 0.427 | 0.659 | |  |
|  |  | St.Dev | 1.898 | 1.601 | 0.249 | 0.249 | 0.171 | 0.091 | 0.131 | 0.163 | |  |
| Huilai (HL) | chr1 | RM283 | 3 | 7 | 42.86 | 1.429 | 1.084 | 0.067 | 0.122 | 0.766 | |  |
|  |  | RM5 | 1 | 3 | 33.33 | 1.333 | 1.030 | 0.028 | 0.060 | 0.431 | |  |
|  |  | RM128 | 3 | 4 | 75.00 | 1.750 | 1.117 | 0.099 | 0.191 | 0.436 | |  |
|  | chr2 | RM236 | 4 | 7 | 57.14 | 1.571 | 1.093 | 0.078 | 0.148 | 0.717 | |  |
|  |  | RM530 | 4 | 4 | 100.00 | 2.000 | 1.654 | 0.383 | 0.568 | 0.646 | |  |
|  |  | RM498 | 4 | 5 | 80.00 | 1.800 | 1.435 | 0.263 | 0.402 | 0.692 | |  |
|  | chr3 | RM175 | 0 | 2 | 0.00 | 1.000 | 1.000 | 0.000 | 0.000 | 0.000 | |  |
|  |  | RM135 | 2 | 4 | 50.00 | 1.500 | 1.177 | 0.115 | 0.184 | 0.585 | |  |
|  |  | RM130 | 6 | 6 | 100.00 | 2.000 | 1.366 | 0.224 | 0.355 | 0.701 | |  |
|  | chr4 | RM518 | 3 | 6 | 50.00 | 1.500 | 1.183 | 0.123 | 0.197 | 0.752 | |  |
|  |  | RM255 | 4 | 4 | 100.00 | 2.000 | 1.454 | 0.273 | 0.424 | 0.516 | |  |
|  |  | RM559 | 2 | 3 | 66.67 | 1.667 | 1.321 | 0.166 | 0.237 | 0.380 | |  |
|  | chr5 | RM413 | 5 | 5 | 100.00 | 2.000 | 1.347 | 0.228 | 0.372 | 0.579 | |  |
|  |  | RM31 | 3 | 4 | 75.00 | 1.750 | 1.539 | 0.308 | 0.450 | 0.665 | |  |
|  |  | RM538 | 1 | 4 | 25.00 | 1.250 | 1.012 | 0.012 | 0.028 | 0.607 | |  |
|  | chr6 | RM204 | 6 | 6 | 100.00 | 2.000 | 1.380 | 0.229 | 0.367 | 0.620 | |  |
|  |  | RM162 | 5 | 5 | 100.00 | 2.000 | 1.396 | 0.240 | 0.382 | 0.143 | |  |
|  |  | RM400 | 2 | 2 | 100.00 | 2.000 | 1.397 | 0.248 | 0.391 | 0.571 | |  |
|  | chr7 | RM180 | 2 | 3 | 66.67 | 1.667 | 1.179 | 0.128 | 0.215 | 0.377 | |  |
|  |  | RM429 | 8 | 8 | 100.00 | 2.000 | 1.544 | 0.316 | 0.477 | 0.795 | |  |
|  |  | RM248 | 2 | 7 | 28.57 | 1.286 | 1.225 | 0.126 | 0.181 | 0.834 | |  |
|  | chr8 | RM407 | 3 | 5 | 60.00 | 1.600 | 1.564 | 0.291 | 0.407 | 0.766 | |  |
|  |  | RM80 | 2 | 3 | 66.67 | 1.667 | 1.427 | 0.240 | 0.357 | 0.506 | |  |
|  |  | RM447 | 9 | 9 | 100.00 | 2.000 | 1.384 | 0.231 | 0.369 | 0.754 | |  |
|  | chr9 | RM444 | 0 | 1 | 0.00 | 1.000 | 1.000 | 0.000 | 0.000 | 0.000 | |  |
|  |  | RM201 | 0 | 1 | 0.00 | 1.000 | 1.000 | 0.000 | 0.000 | 0.000 | |  |
|  |  | RM205 | 9 | 9 | 100.00 | 2.000 | 1.404 | 0.237 | 0.374 | 0.757 | |  |
|  | chr10 | RM216 | 2 | 4 | 50.00 | 1.500 | 1.035 | 0.032 | 0.072 | 0.459 | |  |
|  |  | RM333 | 3 | 3 | 100.00 | 2.000 | 1.661 | 0.397 | 0.586 | 0.561 | |  |
|  |  | RM591 | 11 | 11 | 100.00 | 2.000 | 1.391 | 0.255 | 0.406 | 0.864 | |  |
|  | chr11 | RM167 | 2 | 3 | 66.67 | 1.667 | 1.474 | 0.266 | 0.389 | 0.535 | |  |
|  |  | RM206 | 7 | 7 | 100.00 | 2.000 | 1.424 | 0.254 | 0.391 | 0.738 | |  |
|  |  | RM144 | 3 | 4 | 75.00 | 1.750 | 1.159 | 0.126 | 0.227 | 0.485 | |  |
|  | chr12 | RM20A | 6 | 6 | 100.00 | 2.000 | 1.398 | 0.232 | 0.364 | 0.617 | |  |
|  |  | RM235 | 1 | 3 | 33.33 | 1.333 | 1.073 | 0.060 | 0.108 | 0.231 | |  |
|  |  | RM17 | 6 | 6 | 100.00 | 2.000 | 1.640 | 0.360 | 0.536 | 0.754 | |  |
|  |  | Means | 3.722 | 4.833 | 77.01 | 1.695 | 1.305 | 0.184 | 0.287 | 0.551 | |  |
|  |  | St.Dev | 2.652 | 2.255 | 0.321 | 0.321 | 0.202 | 0.113 | 0.165 | 0.231 | |  |
| Gogang (FG) | chr1 | RM283 | 5 | 5 | 100.00 | 2.000 | 1.388 | 0.248 | 0.389 | 0.603 | |  |
|  |  | RM5 | 5 | 6 | 83.33 | 1.833 | 1.114 | 0.093 | 0.174 | 0.635 | |  |
|  |  | RM128 | 6 | 6 | 100.00 | 2.000 | 1.416 | 0.271 | 0.432 | 0.713 | |  |
|  | chr2 | RM236 | 2 | 5 | 40.00 | 1.096 | 0.066 | 0.108 | 1.096 | 0.672 | |  |
|  |  | RM530 | 7 | 8 | 87.50 | 1.875 | 1.547 | 0.314 | 0.466 | 0.823 | |  |
|  |  | RM498 | 7 | 7 | 100.00 | 1.231 | 0.157 | 0.269 | 1.231 | 0.585 | |  |
|  | chr3 | RM175 | 0 | 4 | 0.00 | 1.000 | 1.000 | 0.000 | 0.000 | 0.000 | |  |
|  |  | RM135 | 3 | 3 | 100.00 | 1.698 | 0.407 | 0.596 | 1.698 | 0.592 | |  |
|  |  | RM130 | 7 | 7 | 100.00 | 2.000 | 1.312 | 0.204 | 0.336 | 0.687 | |  |
|  | chr4 | RM518 | 6 | 6 | 100.00 | 2.000 | 1.495 | 0.293 | 0.444 | 0.710 | |  |
|  |  | RM255 | 2 | 2 | 100.00 | 2.000 | 1.425 | 0.261 | 0.407 | 0.158 | |  |
|  |  | RM559 | 2 | 3 | 66.67 | 1.667 | 1.412 | 0.247 | 0.369 | 0.529 | |  |
|  | chr5 | RM413 | 3 | 4 | 75.00 | 1.750 | 1.213 | 0.129 | 0.206 | 0.422 | |  |
|  |  | RM31 | 3 | 3 | 100.00 | 2.000 | 1.431 | 0.259 | 0.409 | 0.387 | |  |
|  |  | RM538 | 7 | 7 | 100.00 | 2.000 | 1.383 | 0.235 | 0.363 | 0.691 | |  |
|  | chr6 | RM204 | 3 | 4 | 75.00 | 1.750 | 1.515 | 0.305 | 0.447 | 0.669 | |  |
|  |  | RM162 | 7 | 8 | 87.50 | 1.875 | 1.420 | 0.251 | 0.389 | 0.786 | |  |
|  |  | RM400 | 7 | 7 | 100.00 | 2.000 | 1.544 | 0.311 | 0.466 | 0.769 | |  |
|  | chr7 | RM180 | 3 | 4 | 75.00 | 1.750 | 1.249 | 0.172 | 0.279 | 0.548 | |  |
|  |  | RM429 | 5 | 6 | 83.33 | 1.833 | 1.498 | 0.259 | 0.376 | 0.715 | |  |
|  |  | RM248 | 3 | 6 | 50.00 | 1.500 | 1.295 | 0.177 | 0.268 | 0.774 | |  |
|  | chr8 | RM407 | 6 | 6 | 100.00 | 2.000 | 1.457 | 0.275 | 0.426 | 0.683 | |  |
|  |  | RM80 | 8 | 8 | 100.00 | 2.000 | 1.407 | 0.240 | 0.377 | 0.729 | |  |
|  |  | RM447 | 5 | 5 | 100.00 | 2.000 | 1.525 | 0.308 | 0.468 | 0.657 | |  |
|  | chr9 | RM444 | 1 | 2 | 50.00 | 1.500 | 1.149 | 0.115 | 0.195 | 0.267 | |  |
|  |  | RM201 | 3 | 4 | 75.00 | 1.800 | 1.410 | 0.239 | 0.366 | 0.542 | |  |
|  |  | RM205 | 3 | 4 | 75.00 | 1.750 | 1.252 | 0.185 | 0.308 | 0.671 | |  |
|  | chr10 | RM216 | 10 | 10 | 100.00 | 2.000 | 1.313 | 0.194 | 0.313 | 0.763 | |  |
|  |  | RM333 | 4 | 4 | 100.00 | 2.000 | 1.530 | 0.297 | 0.450 | 0.553 | |  |
|  |  | RM591 | 8 | 8 | 100.00 | 2.000 | 1.500 | 0.301 | 0.466 | 0.798 | |  |
|  | chr11 | RM167 | 2 | 5 | 40.00 | 1.400 | 1.070 | 0.057 | 0.105 | 0.666 | |  |
|  |  | RM206 | 9 | 9 | 100.00 | 2.000 | 1.446 | 0.288 | 0.452 | 0.849 | |  |
|  |  | RM144 | 4 | 4 | 100.00 | 2.000 | 1.728 | 0.372 | 0.524 | 0.596 | |  |
|  | chr12 | RM20A | 6 | 6 | 100.00 | 2.000 | 1.648 | 0.368 | 0.545 | 0.760 | |  |
|  |  | RM235 | 2 | 4 | 50.00 | 1.500 | 1.348 | 0.200 | 0.293 | 0.665 | |  |
|  |  | RM17 | 3 | 5 | 60.00 | 1.600 | 1.288 | 0.171 | 0.256 | 0.678 |  |  |
|  |  | Means | 4.639 | 5.440 | 85.27 | 1.857 | 1.388 | 0.233 | 0.360 | 0.621 |  |  |
|  |  | St.Dev | 2.394 | 1.920 | 0.239 | 0.272 | 0.361 | 0.102 | 0.304 | 0.179 |  |  |
| Suixi (SX) | chr1 | RM283 | 7 | 7 | 100.00 | 2.000 | 1.549 | 0.324 | 0.492 | 0.807 |  |  |
|  |  | RM5 | 5 | 7 | 71.43 | 1.714 | 1.244 | 0.175 | 0.290 | 0.800 |  |  |
|  |  | RM128 | 4 | 4 | 100.00 | 2.000 | 1.557 | 0.300 | 0.440 | 0.589 |  |  |
|  | chr2 | RM236 | 5 | 6 | 83.33 | 1.833 | 1.468 | 0.286 | 0.435 | 0.760 |  |  |
|  |  | RM530 | 10 | 11 | 90.91 | 1.909 | 1.445 | 0.272 | 0.415 | 0.855 |  |  |
|  |  | RM498 | 6 | 6 | 100.00 | 2.000 | 1.770 | 0.428 | 0.618 | 0.795 |  |  |
|  | chr3 | RM175 | 5 | 5 | 100.00 | 2.000 | 1.482 | 0.311 | 0.485 | 0.711 |  |  |
|  |  | RM135 | 4 | 5 | 80.00 | 1.800 | 1.516 | 0.284 | 0.418 | 0.698 |  |  |
|  |  | RM130 | 4 | 4 | 100.00 | 1.800 | 1.436 | 0.260 | 0.391 | 0.589 |  |  |
|  | chr4 | RM518 | 4 | 5 | 80.00 | 1.800 | 1.165 | 0.136 | 0.248 | 0.668 |  |  |
|  |  | RM255 | 4 | 4 | 100.00 | 2.000 | 1.667 | 0.364 | 0.533 | 0.612 |  |  |
|  |  | RM559 | 0 | 5 | 0.00 | 1.500 | 1.012 | 0.012 | 0.033 | 0.677 |  |  |
|  | chr5 | RM413 | 6 | 6 | 100.00 | 2.000 | 1.731 | 0.412 | 0.600 | 0.791 |  |  |
|  |  | RM31 | 9 | 9 | 100.00 | 2.000 | 1.242 | 0.183 | 0.318 | 0.800 |  |  |
|  |  | RM538 | 4 | 5 | 80.00 | 1.800 | 1.375 | 0.235 | 0.369 | 0.694 |  |  |
|  | chr6 | RM204 | 8 | 8 | 100.00 | 2.000 | 1.700 | 0.398 | 0.583 | 0.841 |  |  |
|  |  | RM162 | 4 | 4 | 100.00 | 2.000 | 1.628 | 0.358 | 0.530 | 0.612 |  |  |
|  |  | RM400 | 5 | 6 | 83.33 | 1.833 | 1.550 | 0.312 | 0.460 | 0.770 |  |  |
|  | chr7 | RM180 | 4 | 5 | 80.00 | 1.800 | 1.544 | 0.316 | 0.466 | 0.732 |  |  |
|  |  | RM429 | 9 | 9 | 100.00 | 2.000 | 1.265 | 0.201 | 0.344 | 0.695 |  |  |
|  |  | RM248 | 5 | 6 | 83.33 | 1.833 | 1.485 | 0.290 | 0.438 | 0.808 |  |  |
|  | chr8 | RM407 | 5 | 7 | 71.43 | 1.714 | 1.467 | 0.281 | 0.416 | 0.825 |  |  |
|  |  | RM80 | 8 | 8 | 100.00 | 2.000 | 1.647 | 0.374 | 0.553 | 0.839 |  |  |
|  |  | RM447 | 6 | 6 | 100.00 | 2.000 | 1.708 | 0.388 | 0.567 | 0.802 |  |  |
|  | chr9 | RM444 | 4 | 5 | 80.00 | 1.900 | 1.456 | 0.281 | 0.434 | 0.703 |  |  |
|  |  | RM201 | 5 | 5 | 100.00 | 1.900 | 1.456 | 0.281 | 0.434 | 0.709 |  |  |
|  |  | RM205 | 3 | 5 | 60.00 | 1.600 | 1.130 | 0.103 | 0.185 | 0.661 |  |  |
|  | chr10 | RM216 | 5 | 5 | 100.00 | 2.000 | 1.732 | 0.411 | 0.597 | 0.740 |  |  |
|  |  | RM333 | 3 | 5 | 60.00 | 1.600 | 1.094 | 0.079 | 0.149 | 0.647 |  |  |
|  |  | RM591 | 5 | 7 | 71.43 | 1.714 | 1.303 | 0.197 | 0.315 | 0.826 |  |  |
|  | chr11 | RM167 | 4 | 5 | 80.00 | 1.800 | 1.510 | 0.289 | 0.426 | 0.720 |  |  |
|  |  | RM206 | 4 | 5 | 80.00 | 1.800 | 1.611 | 0.325 | 0.469 | 0.725 |  |  |
|  |  | RM144 | 7 | 8 | 87.50 | 1.875 | 1.309 | 0.210 | 0.343 | 0.819 |  |  |
|  | chr12 | RM20A | 5 | 5 | 100.00 | 2.000 | 1.707 | 0.383 | 0.556 | 0.708 |  |  |
|  |  | RM235 | 4 | 5 | 80.00 | 1.600 | 1.580 | 0.295 | 0.411 | 0.762 |  |  |
|  |  | RM17 | 5 | 5 | 100.00 | 2.000 | 1.581 | 0.350 | 0.529 | 0.709 |  |  |
|  |  | Means | 5.139 | 5.917 | 86.85 | 1.862 | 1.473 | 0.281 | 0.426 | 0.736 |  |  |
|  |  | St.Dev | 1.903 | 1.588 | 0.190 | 0.142 | 0.192 | 0.096 | 0.129 | 0.074 |  |  |
| Qionghai (QH) | chr1 | RM283 | 10 | 10 | 100.00 | 2.000 | 1.513 | 0.299 | 0.453 | 0.832 |  |  |
|  |  | RM5 | 4 | 5 | 80.00 | 1.800 | 1.563 | 0.292 | 0.413 | 0.694 |  |  |
|  |  | RM128 | 2 | 3 | 66.67 | 1.667 | 1.156 | 0.126 | 0.224 | 0.405 |  |  |
|  | chr2 | RM236 | 5 | 7 | 71.43 | 1.714 | 1.282 | 0.160 | 0.248 | 0.732 |  |  |
|  |  | RM530 | 8 | 8 | 100.00 | 2.000 | 1.963 | 0.491 | 0.684 | 0.711 |  |  |
|  |  | RM498 | 7 | 8 | 87.50 | 1.667 | 1.015 | 0.014 | 0.039 | 0.078 |  |  |
|  | chr3 | RM175 | 4 | 4 | 100.00 | 2.000 | 1.815 | 0.428 | 0.612 | 0.661 |  |  |
|  |  | RM135 | 4 | 5 | 80.00 | 1.800 | 1.450 | 0.275 | 0.419 | 0.736 |  |  |
|  |  | RM130 | 7 | 7 | 100.00 | 2.000 | 1.368 | 0.249 | 0.401 | 0.740 |  |  |
|  | chr4 | RM518 | 7 | 7 | 100.00 | 2.000 | 1.419 | 0.253 | 0.399 | 0.717 |  |  |
|  |  | RM255 | 11 | 11 | 100.00 | 2.000 | 1.338 | 0.208 | 0.328 | 0.783 |  |  |
|  |  | RM559 | 6 | 6 | 100.00 | 2.000 | 1.455 | 0.252 | 0.376 | 0.634 |  |  |
|  | chr5 | RM413 | 7 | 7 | 100.00 | 2.000 | 1.269 | 0.164 | 0.265 | 0.554 |  |  |
|  |  | RM31 | 14 | 14 | 100.00 | 2.000 | 1.267 | 0.166 | 0.271 | 0.801 |  |  |
|  |  | RM538 | 7 | 7 | 100.00 | 2.000 | 1.449 | 0.269 | 0.414 | 0.736 |  |  |
|  | chr6 | RM204 | 4 | 4 | 100.00 | 2.000 | 1.204 | 0.149 | 0.256 | 0.416 |  |  |
|  |  | RM162 | 5 | 5 | 100.00 | 2.000 | 1.419 | 0.256 | 0.392 | 0.596 |  |  |
|  |  | RM400 | 2 | 3 | 66.67 | 1.667 | 1.094 | 0.075 | 0.136 | 0.385 |  |  |
|  | chr7 | RM180 | 11 | 11 | 100.00 | 2.000 | 1.475 | 0.286 | 0.434 | 0.859 |  |  |
|  |  | RM429 | 1 | 4 | 25.00 | 1.250 | 1.245 | 0.124 | 0.172 | 0.697 |  |  |
|  |  | RM248 | 5 | 5 | 100.00 | 2.000 | 1.233 | 0.155 | 0.264 | 0.420 |  |  |
|  | chr8 | RM407 | 0 | 5 | 0.00 | 1.000 | 1.000 | 0.000 | 0.000 | 0.768 |  |  |
|  |  | RM80 | 0 | 2 | 0.00 | 1.000 | 1.000 | 0.000 | 0.000 | 0.375 |  |  |
|  |  | RM447 | 8 | 8 | 100.00 | 2.000 | 1.446 | 0.255 | 0.390 | 0.751 |  |  |
|  | chr9 | RM444 | 0 | 1 | 0.00 | 1.000 | 1.000 | 0.000 | 0.000 | 0.000 |  |  |
|  |  | RM201 | 0 | 1 | 0.00 | 1.000 | 1.000 | 0.000 | 0.000 | 0.000 |  |  |
|  |  | RM205 | 7 | 7 | 100.00 | 2.000 | 1.352 | 0.211 | 0.337 | 0.656 |  |  |
|  | chr10 | RM216 | 8 | 8 | 100.00 | 2.000 | 1.445 | 0.268 | 0.409 | 0.787 |  |  |
|  |  | RM333 | 8 | 8 | 100.00 | 2.000 | 1.293 | 0.186 | 0.307 | 0.676 |  |  |
|  |  | RM591 | 8 | 8 | 100.00 | 2.000 | 1.285 | 0.167 | 0.269 | 0.597 |  |  |
|  | chr11 | RM167 | 11 | 11 | 100.00 | 2.000 | 1.351 | 0.211 | 0.334 | 0.788 |  |  |
|  |  | RM206 | 5 | 5 | 100.00 | 2.000 | 1.179 | 0.112 | 0.186 | 0.215 |  |  |
|  |  | RM144 | 6 | 6 | 100.00 | 2.000 | 1.217 | 0.143 | 0.240 | 0.463 |  |  |
|  | chr12 | RM20A | 0 | 2 | 0.00 | 1.000 | 1.000 | 0.000 | 0.000 | 0.375 |  |  |
|  |  | RM235 | 2 | 3 | 66.67 | 1.667 | 1.422 | 0.259 | 0.384 | 0.544 |  |  |
|  |  | RM17 | 7 | 7 | 100.00 | 2.000 | 1.343 | 0.202 | 0.316 | 0.636 |  |  |
|  |  | Means | 5.583 | 6.194 | 90.13 | 1.784 | 1.314 | 0.186 | 0.288 | 0.578 |  |  |
|  |  | St.Dev | 3.531 | 2.942 | 0.353 | 0.353 | 0.214 | 0.114 | 0.165 | 0.227 |  |  |

PPB, na, ne, h, I, and PIC are indicating percentage of polymorphic bands, number of alleles, effective number of alleles, Nei's gene diversity, Shannon's information index, and polymorphism information content, respectively.
