# Supplementary material for: Evaluation of Genetic Diversity and Development of a Core Collection of Wild Rice (Oryza rufipogon Griff.) Populations in China
Source: PLoS One. 2015 Dec 31;10(12):e0145990. doi: 10.1371/journal.pone.0145990 (PMC4703137; doi:10.1371/journal.pone.0145990)
Supplement: S5 Table — (DOCX) [file pone.0145990.s006.docx]

**S5 Table. Nei's genetic identity and genetic distance among eight populations.**

| Population | DX | BL | ZC | GZ | HL | FG | SX | QH |
| --- | --- | --- | --- | --- | --- | --- | --- | --- |
| DX |  | 0.852 | 0.746 | 0.844 | 0.832 | 0.814 | 0.810 | 0.805 |
| BL | 0.160 |  | 0.722 | 0.851 | 0.832 | 0.820 | 0.761 | 0.823 |
| ZC | 0.294 | 0.326 |  | 0.760 | 0.736 | 0.732 | 0.732 | 0.740 |
| GZ | 0.170 | 0.162 | 0.275 |  | 0.835 | 0.834 | 0.812 | 0.846 |
| HL | 0.184 | 0.184 | 0.307 | 0.181 |  | 0.799 | 0.771 | 0.816 |
| FG | 0.206 | 0.198 | 0.312 | 0.182 | 0.224 |  | 0.788 | 0.828 |
| SX | 0.210 | 0.273 | 0.312 | 0.208 | 0.261 | 0.238 |  | 0.772 |
| QH | 0.217 | 0.194 | 0.301 | 0.167 | 0.203 | 0.189 | 0.258 |  |

Upper and lower halves of table indicates Nei's genetic identity and genetic distance, respectively.

Dongxiang (DX) from Jiangxi province, Qionghai (QH) from Hainan province, and Fogang (FG), Boluo (BL), Zengcheng (ZC), Huilai (HL), Gaozhou (GZ) and Suixi (SX) from Guangdong province.
