# Supplementary material for: Evaluation of Genetic Diversity and Development of a Core Collection of Wild Rice (Oryza rufipogon Griff.) Populations in China
Source: PLoS One. 2015 Dec 31;10(12):e0145990. doi: 10.1371/journal.pone.0145990 (PMC4703137; doi:10.1371/journal.pone.0145990)
Supplement: S6 Table — (DOCX) [file pone.0145990.s007.docx]

**S6 Table. Comparison of the core collections developed by software and genetic distance**

| Population | Genetic distance | Software | Common accessions |
| --- | --- | --- | --- |
| Dongxiang | 4w-2, 4w-4, 4w-22, 4w-26, 4w-27, 4w-28, 4w-38, 4w-41, 4w-47, 4w-55, 4w-56, 4w-80, 4w-81, 4w-86, 4w-95, 4w-96, 4w-102, 4w-103, 4w-105, 4w-113, 4w-120, 4w-143 | 4w-3, 4w-4, 4w-7, 4w-27, 4w-30, 4w-33, 4w-37, 4w-46, 4w-47, 4w-48, 4w-55, 4w-58, 4w-65, 4w-83, 4w-86, 4w-93, 4w-95, 4w-99, 4w-103, 4w-120, 4w-126, 4w-140 | 4w-4, 4w-27, 4w-47, 4w-55, 4w-86, 4w-95, 4w-103, 4w-120 |
| Boluo | 2w-6, 2w-7, 2w-10, 2w-15, 2w-16, 2w-34, 2w-36, 2w-39, 2w-40, 2w-50, 2w-67, 2w-69 | 2w-4, 2w-7, 2w-20, 2w-23, 2w-34, 2w-36, 2w-44, 2w-48, 2w-61, 2w-67, 2w-69, 2w-80 | 2w-69, 2w-36, 2w-7, 2w-67, 2w-34 |
| Zengcheng | 26w-1, 26w-4, 26w-12, 26w-23, 26w-25, 26w-42, 26w-49, 26w-58, 26w-60, 26w-66, 26w-68, 26w-76, 26w-77, 26w-78, 26w-90, 26w-95, 26w-97, 26w-98, 26w-107, 26w-112, 26w-121, 26w-130, 26w-132, 26w-143 | 26w-1, 26w-4, 26w-12, 26w-15, 26w-16, 26w-19, 26w-25, 26w-47, 26w-49, 26w-60, 26w-68, 26w-77, 26w-78, 26w-84, 26w-90, 26w-92, 26w-95, 26w-98, 26w-125, 26w-127, 26w-129, 26w-138, 26w-140, 26w-146 | 26w-1, 26w-4, 26w-12, 26w-25, 26w-49, 26w-60, 26w-68, 26w-77, 26w-78, 26w-90, 26w-95, 26w-98 |
| Gaozhou | 7w-1, 7w-3, 7w-12, 7w-27, 7w-47, 7w-67, 7w-76, 7w-89, 7w-90, 7w-98, 7w-103, 7w-111, 7w-115, 7w-128, 7w-133, 7w-135, 7w-143, 7w-144, 7w-178, 7w-179, 7w-180, 7w-183, 7w-184, 7w-186, 7w-190 | 7w-2, 7w-3, 7w-12, 7w-37, 7w-40, 7w-47, 7w-67, 7w-79, 7w-90, 7w-98, 7w-103, 7w-108, 7w-130, 7w-134, 7w-143, 7w-160, 7w-164, 7w-171, 7w-173, 7w-178, 7w-179, 7w-180, 7w-183, 7w-184, 7w-190 | 7w-3, 7w-12, 7w-47, 7w-67, 7w-90, 7w-98, 7w-103, 7w-143, 7w-178, 7w-179, 7w-180, 7w-183, 7w-184, 7w-190 |
| Huilai | 18w-3, 18w-5, 18w-6, 18w-28, 18w-30, 18w-39, 18w-60, 18w-66, 18w-67, 18w-77, 18w-82, 18w-95, 18w-99 | 18w-28, 18w-30, 18w-31, 18w-41, 18w-58, 18w-65, 18w-66, 18w-67, 18w-72, 18w-73, 18w-82, 18w-86, 18w-99 | 18w-28, 18w-30, 18w-66, 18w-67, 18w-82, 18w-99 |
| Fogang | 6w-1, 6w-12, 6w-13, 6w-51, 6w-59, 6w-60, 6w-61, 6w-67, 6w-73, 6w-92 | 6w-4, 6w-13, 6w-14, 6w-15, 6w-36, 6w-47, 6w-48, 6w-51, 6w-52, 6w-89 | 6w-13, 6w-51 |
| Suixi | 19w-9, 19w-16, 19w-21, 19w-27, 19w-37, 19w-44, 19w-45, 19w-60, 19w-62, 19w-80 | 19w-21, 19w-28, 19w-57, 19w-60, 19w-62, 19w-66, 19w-68, 19w-71, 19w-77, 19w-80 | 19w-21, 19w-60, 19w-80, 19w-62 |
| Qinghai | 17w-4, 17w-8, 17w-19, 17w-25, 17w-41, 17w-57, 17w-58, 17w-61, 17w-62, 17w-64, 17w-66, 17w-67, 17w-68, 17w-71 | 17w-10, 17w-19, 17w-25, 17w-41, 17w-57, 17w-58, 17w-59, 17w-61, 17w-63, 17w-64, 17w-66, 17w-69, 17w-70, 17w-71 | 17w-19, 17w-25, 17w-41, 17w-57, 17w-58, 17w-61, 17w-64, 17w-66, 17w-71 |
