# Supplementary material for: Evaluation of Genetic Diversity and Development of a Core Collection of Wild Rice (Oryza rufipogon Griff.) Populations in China
Source: PLoS One. 2015 Dec 31;10(12):e0145990. doi: 10.1371/journal.pone.0145990 (PMC4703137; doi:10.1371/journal.pone.0145990)
Supplement: S7 Table — (DOCX) [file pone.0145990.s008.docx]

**S7 Table. Morphological traits data of 130 accessions selected as the core collection.**

| Material NO. | Panicle length (cm) | Secondary branches | Awn length (mm) | Number of spikelets per panicle | Filled spikelets per panicle | Seed set (%) | Spikelet length (mm/ten spikelets) | Spikelet width (mm/ten spikelets) | Spikelet length-width ratio | Plant height (cm) | Flowering | Growth habit | Leaf color |
| --- | --- | --- | --- | --- | --- | --- | --- | --- | --- | --- | --- | --- | --- |
| 4w-2 | 15.00 | 0.00 | 54.50 | 22.50 | 19.00 | 84.44 | 7.85 | 2.25 | 3.49 | 125 | 1 | 2 | 2 |
| 4w-4 | 19.45 | 0.50 | 65.00 | 43.00 | 27.00 | 62.79 | 8.00 | 2.20 | 3.64 | 152 | 1 | 2 | 1 |
| 4w-22 | 16.70 | 0.00 | 60.00 | 27.00 | 7.00 | 25.93 | 8.60 | 2.30 | 3.74 | 100 | 1 | 3 | 1 |
| 4w-26 | 12.60 | 2.00 | 71.00 | 23.00 | 5.00 | 21.74 | 8.00 | 2.00 | 4.00 | 33 | 1 | 1 | 2 |
| 4w-27 | 15.50 | 2.50 | 48.00 | 31.00 | 6.00 | 19.35 | 7.85 | 2.30 | 3.41 | 155 | 1 | 3 | 2 |
| 4w-28 | 7.30 | 0.00 | 37.00 | 10.00 | 5.00 | 50.00 | 8.10 | 2.35 | 3.45 | 60 | 1 | 3 | 1 |
| 4w-38 | 18.40 | 1.00 | 49.00 | 29.00 | 16.00 | 55.17 | 7.50 | 2.35 | 3.19 | 132 | 1 | 2 | 2 |
| 4w-41 | 22.80 | 2.00 | 64.00 | 51.50 | 35.00 | 67.96 | 8.05 | 2.35 | 3.43 | 180 | 1 | 2 | 2 |
| 4w-47 | 14.95 | 0.00 | 62.50 | 23.50 | 2.00 | 8.51 | 7.80 | 2.60 | 3.00 | 125 | 1 | 2 | 2 |
| 4w-55 | 0.00 | 0.00 | 0.00 | 0.00 | 0.00 | 0.00 | 0.00 | 0.00 | 0.00 | 99 | 2 | 3 | 2 |
| 4w-56 | 0.00 | 0.00 | 0.00 | 0.00 | 0.00 | 0.00 | 0.00 | 0.00 | 0.00 | 160 | 2 | 3 | 2 |
| 4W-80 | 21.80 | 2.00 | 66.00 | 51.50 | 35.00 | 55.50 | 8.05 | 2.35 | 3.43 | 184 | 1 | 2 | 2 |
| 4w-81 | 11.65 | 0.00 | 48.50 | 26.00 | 19.00 | 73.08 | 7.15 | 2.25 | 3.19 | 88 | 1 | 2 | 2 |
| 4w-86 | 13.20 | 0.00 | 74.00 | 25.00 | 11.00 | 44.00 | 7.65 | 2.15 | 3.58 | 73 | 1 | 3 | 1 |
| 4w-95 | 11.43 | 0.33 | 62.00 | 19.67 | 9.00 | 45.76 | 8.17 | 2.17 | 3.77 | 88 | 1 | 1 | 2 |
| 4w-96 | 0.00 | 0.00 | 0.00 | 0.00 | 0.00 | 0.00 | 0.00 | 0.00 | 0.00 | 123 | 2 | 3 | 2 |
| 4w-102 | 10.50 | 0.00 | 56.50 | 16.00 | 11.00 | 68.75 | 7.35 | 2.25 | 3.27 | 92 | 1 | 3 | 2 |
| 4w-103 | 4.80 | 0.00 | 61.00 | 5.00 | 4.00 | 80.00 | 7.70 | 2.00 | 3.85 | 43 | 1 | 4 | 1 |
| 4w-105 | 6.70 | 0.00 | 41.00 | 9.00 | 6.00 | 66.67 | 8.00 | 2.20 | 3.64 | 63 | 1 | 1 | 1 |
| 4w-113 | 16.00 | 4.50 | 56.00 | 47.50 | 16.00 | 33.68 | 8.15 | 2.25 | 3.62 | 90 | 1 | 2 | 2 |
| 4w-120 | 16.05 | 6.50 | 41.00 | 49.50 | 40.00 | 80.81 | 7.85 | 2.35 | 3.34 | 90 | 1 | 2 | 2 |
| 4w-143 | 20.45 | 3.00 | 58.50 | 47.00 | 18.00 | 38.30 | 8.20 | 2.30 | 3.57 | 107 | 1 | 3 | 2 |
| 2w-6 | 10.93 | 0.00 | 63.33 | 16.00 | 5.67 | 35.42 | 130.33 | 21.67 | 6.02 | 50 | 1 | 1 | 2 |
| 2w-7 | 0.00 | 0.00 | 0.00 | 0.00 | 0.00 | 0.00 | 0.00 | 0.00 | 0.00 | 44 | 2 | 1 | 2 |
| 2w-10 | 13.73 | 0.33 | 48.33 | 84.67 | 16.33 | 19.29 | 71.33 | 17.33 | 4.12 | 100 | 1 | 3 | 2 |
| 2w-15 | 13.90 | 0.00 | 48.33 | 61.00 | 16.67 | 27.32 | 70.67 | 20.33 | 3.48 | 82 | 1 | 2 | 1 |
| 2w-16 | 19.43 | 0.00 | 61.00 | 68.33 | 15.67 | 22.93 | 80.33 | 20.00 | 4.02 | 111 | 1 | 4 | 1 |
| 2w-34 | 16.87 | 0.00 | 69.33 | 51.00 | 17.00 | 33.33 | 79.00 | 17.33 | 4.56 | 105 | 1 | 3 | 2 |
| 2w-36 | 17.50 | 0.00 | 71.00 | 55.67 | 17.33 | 31.14 | 71.00 | 21.33 | 3.33 | 100 | 1 | 4 | 1 |
| 2w-39 | 0.00 | 0.00 | 0.00 | 0.00 | 0.00 | 0.00 | 0.00 | 0.00 | 0.00 | 110 | 2 | 2 | 2 |
| 2w-40 | 9.17 | 0.00 | 80.67 | 15.67 | 3.33 | 21.28 | 96.00 | 18.67 | 5.14 | 70 | 1 | 1 | 2 |
| 2w-50 | 7.00 | 0.00 | 73.33 | 16.67 | 2.67 | 16.00 | 70.00 | 20.00 | 3.50 | 25 | 2 | 1 | 1 |
| 2w-67 | 18.50 | 0.00 | 80.00 | 34.33 | 14.67 | 42.72 | 80.33 | 20.67 | 3.89 | 98 | 1 | 4 | 1 |
| 2w-69 | 16.97 | 0.00 | 63.33 | 32.33 | 18.33 | 56.70 | 91.33 | 21.00 | 4.35 | 125 | 1 | 4 | 1 |
| 26w-1 | 11.50 | 0.00 | 59.67 | 21.67 | 8.00 | 36.92 | 80.33 | 22.67 | 3.54 | 93 | 1 | 1 | 1 |
| 26w-4 | 7.17 | 0.00 | 115.50 | 13.00 | 4.50 | 34.62 | 81.00 | 22.00 | 3.68 | 0 | 2 | 3 | 2 |
| 26w-12 | 15.50 | 1.33 | 61.67 | 42.00 | 15.00 | 35.71 | 80.67 | 23.67 | 3.41 | 115 | 1 | 2 | 2 |
| 26w-23 | 8.83 | 0.00 | 60.67 | 12.67 | 3.33 | 26.32 | 86.00 | 23.00 | 3.74 | 58 | 1 | 1 | 1 |
| 26w-25 | 11.13 | 0.00 | 56.00 | 13.33 | 2.00 | 15.00 | 85.00 | 24.00 | 3.54 | 64 | 1 | 1 | 2 |
| 26w-42 | 21.90 | 12.67 | 73.33 | 102.00 | 17.33 | 16.99 | 81.33 | 24.00 | 3.39 | 72 | 1 | 4 | 2 |
| 26w-49 | 11.27 | 0.00 | 69.00 | 21.00 | 7.00 | 33.33 | 80.67 | 23.00 | 3.51 | 65 | 1 | 1 | 1 |
| 26w-58 | 10.87 | 0.00 | 64.33 | 18.67 | 9.00 | 48.21 | 81.67 | 23.00 | 3.55 | 60 | 1 | 1 | 1 |
| 26w-60 | 16.30 | 1.50 | 58.00 | 20.67 | 17.67 | 85.48 | 83.33 | 22.67 | 3.68 | 102 | 1 | 2 | 2 |
| 26w-66 | 9.47 | 0.00 | 67.67 | 16.33 | 13.67 | 83.69 | 80.00 | 23.00 | 3.48 | 96 | 1 | 2 | 2 |
| 26w-68 | 19.03 | 6.00 | 79.33 | 73.00 | 15.67 | 21.46 | 80.00 | 25.67 | 3.12 | 140 | 1 | 3 | 2 |
| 26w-76 | 10.83 | 0.00 | 71.67 | 18.67 | 14.67 | 78.57 | 78.33 | 23.00 | 3.41 | 68 | 1 | 3 | 2 |
| 26w-77 | 27.47 | 11.33 | 75.67 | 101.67 | 10.00 | 9.84 | 78.33 | 22.67 | 3.46 | 164 | 1 | 3 | 2 |
| 26w-78 | 17.87 | 1.50 | 82.33 | 33.33 | 13.00 | 39.00 | 83.67 | 22.00 | 3.80 | 102 | 1 | 3 | 2 |
| 26w-90 | 18.07 | 7.33 | 61.67 | 57.67 | 3.00 | 5.20 | 0.00 | 0.00 | 0.00 | 82 | 1 | 3 | 2 |
| 26w-95 | 13.13 | 0.00 | 48.67 | 19.33 | 11.67 | 60.34 | 84.33 | 23.67 | 3.56 | 78 | 1 | 1 | 1 |
| 26w-97 | 22.03 | 9.33 | 59.33 | 82.00 | 48.00 | 58.54 | 83.33 | 24.00 | 3.47 | 137 | 1 | 4 | 2 |
| 26w-98 | 17.70 | 5.00 | 59.67 | 54.00 | 13.67 | 25.31 | 88.33 | 24.00 | 3.68 | 86 | 1 | 4 | 1 |
| 26w-107 | 19.80 | 12.00 | 67.33 | 92.00 | 28.00 | 30.43 | 81.33 | 25.00 | 3.25 | 127 | 1 | 4 | 2 |
| 26w-112 | 16.50 | 0.00 | 76.00 | 21.00 | 10.00 | 47.62 | 85.50 | 23.50 | 3.64 | 120 | 1 | 2 | 2 |
| 26w-121 | 12.10 | 0.00 | 59.33 | 26.00 | 8.00 | 30.77 | 81.00 | 23.50 | 3.45 | 82 | 1 | 2 | 1 |
| 26w-130 | 15.30 | 1.67 | 59.33 | 37.00 | 4.00 | 10.81 | 80.00 | 22.00 | 3.64 | 94 | 1 | 3 | 1 |
| 26w-132 | 14.37 | 1.00 | 60.67 | 31.00 | 19.67 | 63.44 | 78.00 | 23.67 | 3.30 | 129 | 1 | 2 | 2 |
| 26w-143 | 18.97 | 5.33 | 80.33 | 53.00 | 15.33 | 28.93 | 81.33 | 23.00 | 3.54 | 106 | 1 | 4 | 2 |
| 7w-1 | 170.33 | 0.67 | 69.00 | 38.00 | 6.33 | 16.66 | 81.00 | 24.00 | 3.38 | 89 | 1 | 2 | 1 |
| 7w-3 | 159.00 | 0.00 | 80.33 | 25.00 | 17.67 | 70.68 | 82.00 | 23.00 | 3.57 | 88 | 1 | 3 | 2 |
| 7w-12 | 0.00 | 0.00 | 0.00 | 0.00 | 0.00 | 0.00 | 0.00 | 0.00 | 0.00 | 0 | 2 | 1 | 1 |
| 7w-27 | 241.00 | 3.00 | 86.00 | 73.33 | 60.00 | 81.82 | 80.00 | 25.00 | 3.20 | 146 | 1 | 2 | 1 |
| 7w-47 | 0.00 | 0.00 | 0.00 | 0.00 | 0.00 | 0.00 | 0.00 | 0.00 | 0.00 | 0 | 1 | 2 | 2 |
| 7w-67 | 201.50 | 0.00 | 79.50 | 32.50 | 4.50 | 13.85 | 88.00 | 23.00 | 3.83 | 118 | 1 | 2 | 1 |
| 7w-76 | 263.33 | 4.33 | 76.00 | 75.33 | 3.75 | 4.98 | 76.00 | 25.00 | 3.04 | 107 | 1 | 2 | 2 |
| 7w-89 | 185.00 | 1.00 | 73.67 | 34.67 | 14.00 | 40.38 | 75.00 | 23.00 | 3.26 | 117 | 2 | 2 | 2 |
| 7w-90 | 210.33 | 4.33 | 64.33 | 67.33 | 51.83 | 76.98 | 78.00 | 23.00 | 3.39 | 109 | 1 | 2 | 2 |
| 7w-98 | 220.33 | 6.00 | 59.33 | 72.33 | 48.00 | 66.36 | 82.00 | 24.00 | 3.42 | 123 | 1 | 3 | 2 |
| 7w-103 | 180.00 | 4.00 | 75.00 | 64.33 | 11.71 | 18.21 | 75.00 | 23.00 | 3.26 | 132 | 1 | 2 | 2 |
| 7w-111 | 133.67 | 0.00 | 59.67 | 24.00 | 18.00 | 75.00 | 79.00 | 22.00 | 3.59 | 96 | 1 | 2 | 2 |
| 7w-115 | 250.80 | 10.67 | 64.83 | 103.33 | 95.00 | 91.94 | 81.00 | 22.00 | 3.68 | 132 | 1 | 4 | 2 |
| 7w-128 | 179.00 | 0.00 | 81.33 | 26.25 | 19.25 | 73.33 | 84.00 | 22.00 | 3.82 | 111 | 1 | 2 | 2 |
| 7w-133 | 88.00 | 0.00 | 30.00 | 15.00 | 8.00 | 53.33 | 80.00 | 22.00 | 3.64 | 77 | 1 | 1 | 1 |
| 7w-135 | 148.00 | 0.33 | 65.67 | 33.33 | 38.25 | 114.75 | 78.00 | 23.00 | 3.39 | 108 | 1 | 1 | 1 |
| 7w-143 | 280.33 | 11.67 | 71.00 | 97.00 | 17.00 | 17.53 | 72.00 | 23.00 | 3.13 | 137 | 1 | 2 | 2 |
| 7w-144 | 129.00 | 0.00 | 108.00 | 21.00 | 10.00 | 47.62 | 83.00 | 23.00 | 3.61 | 105 | 1 | 1 | 1 |
| 7w-178 | 198.33 | 0.67 | 45.00 | 39.00 | 24.75 | 63.46 | 84.00 | 23.00 | 3.65 | 108 | 1 | 2 | 2 |
| 7w-179 | 150.00 | 0.00 | 56.00 | 27.50 | 2.00 | 7.27 | 80.00 | 25.00 | 3.20 | 64 | 1 | 1 | 1 |
| 7w-180 | 157.33 | 2.00 | 57.33 | 31.33 | 18.67 | 59.59 | 77.00 | 23.00 | 3.35 | 111 | 1 | 3 | 2 |
| 7w-183 | 226.00 | 1.00 | 72.50 | 62.67 | 44.00 | 70.21 | 78.00 | 24.00 | 3.25 | 120 | 1 | 3 | 2 |
| 7w-184 | 111.33 | 0.00 | 88.60 | 16.00 | 3.80 | 23.75 | 80.00 | 27.00 | 2.96 | 103 | 1 | 2 | 2 |
| 7w-186 | 192.83 | 3.00 | 80.83 | 32.20 | 10.00 | 31.06 | 82.00 | 25.00 | 3.28 | 135 | 1 | 4 | 2 |
| 7w-190 | 160.00 | 2.00 | 73.67 | 37.86 | 14.86 | 39.25 | 76.00 | 23.00 | 3.30 | 115 | 1 | 2 | 2 |
| 18w-3 | 8.03 | 0.00 | 75.23 | 8.33 | 5.33 | 63.99 | 91.00 | 23.00 | 3.96 | 64 | 1 | 3 | 2 |
| 18w-5 | 9.63 | 0.00 | 45.33 | 12.00 | 6.50 | 54.17 | 82.00 | 23.00 | 3.57 | 70 | 1 | 3 | 2 |
| 18w-6 | 14.57 | 0.67 | 73.00 | 23.00 | 10.75 | 46.74 | 84.00 | 22.00 | 3.82 | 110 | 1 | 2 | 1 |
| 18w-28 | 0.00 | 0.00 | 0.00 | 0.00 | 0.00 | 0.00 | 0.00 | 0.00 | 0.00 | 0 | 2 | 2 | 2 |
| 18w-30 | 19.77 | 11.00 | 62.67 | 90.20 | 47.20 | 52.33 | 78.00 | 24.00 | 3.25 | 126 | 1 | 4 | 2 |
| 18w-39 | 16.77 | 0.33 | 71.67 | 23.82 | 10.27 | 43.12 | 83.00 | 25.00 | 3.32 | 120 | 1 | 3 | 2 |
| 18w-60 | 11.47 | 0.00 | 78.17 | 14.83 | 9.42 | 63.52 | 80.00 | 22.00 | 3.64 | 130 | 1 | 1 | 1 |
| 18w-66 | 9.32 | 0.00 | 59.90 | 18.50 | 12.50 | 67.57 | 78.00 | 22.00 | 3.55 | 90 | 1 | 1 | 1 |
| 18w-67 | 15.50 | 1.33 | 69.33 | 38.00 | 32.40 | 85.26 | 80.00 | 23.00 | 3.49 | 85 | 1 | 3 | 2 |
| 18w-77 | 8.85 | 0.00 | 61.83 | 19.50 | 13.50 | 69.23 | 78.00 | 24.00 | 3.25 | 37 | 1 | 2 | 2 |
| 18w-82 | 17.62 | 1.67 | 62.93 | 34.40 | 23.60 | 68.60 | 84.00 | 25.00 | 3.36 | 110 | 1 | 3 | 2 |
| 18w-95 | 21.82 | 0.67 | 66.70 | 65.25 | 36.25 | 55.56 | 84.00 | 20.00 | 4.20 | 102 | 1 | 1 | 1 |
| 18w-99 | 7.52 | 0.00 | 73.20 | 10.43 | 4.00 | 38.35 | 86.00 | 24.00 | 3.58 | 80 | 1 | 2 | 2 |
| 6w-1 | 0.00 | 0.00 | 0.00 | 0.00 | 0.00 | 0.00 | 0.00 | 0.00 | 0.00 | 0 | 2 | 1 | 1 |
| 6w-12 | 190.67 | 0.00 | 65.00 | 29.33 | 15.40 | 52.50 | 88.00 | 25.00 | 3.52 | 135 | 1 | 4 | 1 |
| 6w-13 | 171.00 | 3.33 | 75.00 | 47.67 | 18.25 | 38.29 | 76.00 | 25.00 | 3.04 | 116 | 1 | 4 | 1 |
| 6w-51 | 0.00 | 0.00 | 0.00 | 0.00 | 0.00 | 0.00 | 0.00 | 0.00 | 0.00 | 0 | 2 | 2 | 1 |
| 6w-59 | 139.00 | 0.00 | 63.00 | 18.50 | 15.00 | 81.08 | 84.00 | 24.00 | 3.50 | 88 | 1 | 2 | 1 |
| 6w-60 | 199.00 | 1.00 | 52.00 | 31.67 | 6.67 | 21.06 | 86.00 | 26.00 | 3.31 | 88 | 1 | 3 | 1 |
| 6w-61 | 240.67 | 3.67 | 68.67 | 41.00 | 7.00 | 17.07 | 87.00 | 34.00 | 2.56 | 93 | 1 | 2 | 1 |
| 6w-67 | 178.00 | 0.00 | 68.67 | 25.00 | 18.00 | 72.00 | 78.00 | 24.00 | 3.25 | 92 | 1 | 2 | 1 |
| 6w-73 | 172.00 | 0.50 | 77.67 | 30.50 | 7.50 | 24.59 | 83.00 | 26.00 | 3.19 | 120 | 1 | 4 | 2 |
| 6w-92 | 106.00 | 0.00 | 59.00 | 45.00 | 7.00 | 15.56 | 81.00 | 24.00 | 3.38 | 104 | 1 | 2 | 2 |
| 19w-9 | 26.77 | 0.00 | 80.33 | 41.00 | 3.00 | 7.32 | 81.33 | 22.33 | 3.64 | 193 | 1 | 4 | 1 |
| 19w-16 | 0.00 | 0.00 | 0.00 | 0.00 | 0.00 | 0.00 | 0.00 | 0.00 | 0.00 | 147 | 2 | 3 | 1 |
| 19w-21 | 22.47 | 0.33 | 64.67 | 38.33 | 1.00 | 2.61 | 79.67 | 20.33 | 3.92 | 158 | 1 | 4 | 1 |
| 19w-27 | 0.00 | 0.00 | 0.00 | 0.00 | 0.00 | 0.00 | 0.00 | 0.00 | 0.00 | 38 | 2 | 1 | 1 |
| 19w-37 | 0.00 | 0.00 | 0.00 | 0.00 | 0.00 | 0.00 | 0.00 | 0.00 | 0.00 | 113 | 2 | 4 | 1 |
| 19w-44 | 23.90 | 0.67 | 63.67 | 51.67 | 15.33 | 29.68 | 74.33 | 22.67 | 3.28 | 66 | 1 | 3 | 1 |
| 19w-45 | 16.97 | 0.00 | 63.00 | 30.00 | 7.67 | 25.56 | 82.33 | 23.33 | 3.53 | 87 | 1 | 3 | 1 |
| 19w-60 | 14.70 | 0.00 | 57.33 | 28.00 | 0.00 | 0.00 | 77.33 | 22.33 | 3.47 | 93 | 1 | 2 | 1 |
| 19w-62 | 24.17 | 0.67 | 75.33 | 51.33 | 22.67 | 44.16 | 76.00 | 23.33 | 3.26 | 144 | 1 | 2 | 1 |
| 19w-80 | 0.00 | 0.00 | 0.00 | 0.00 | 0.00 | 0.00 | 0.00 | 0.00 | 0.00 | 49 | 2 | 2 | 1 |
| 17w-4 | 20.67 | 2.33 | 80.00 | 0.00 | 44.67 | 0.00 | 0.00 | 0.00 | 0.00 | 123 | 1 | 3 | 2 |
| 17w-8 | 20.70 | 0.33 | 59.67 | 0.00 | 32.00 | 0.00 | 0.00 | 0.00 | 0.00 | 75 | 1 | 3 | 2 |
| 17w-19 | 13.53 | 2.67 | 45.33 | 4.00 | 39.67 | 10.08 | 82.33 | 24.67 | 3.34 | 110 | 1 | 4 | 2 |
| 17w-25 | 18.00 | 0.33 | 80.67 | 25.71 | 44.00 | 58.44 | 81.50 | 22.33 | 3.65 | 139.5 | 1 | 1 | 1 |
| 17w-41 | 18.20 | 5.00 | 85.67 | 1.33 | 66.67 | 2.00 | 80.63 | 26.38 | 3.06 | 94 | 1 | 4 | 2 |
| 17w-57 | 17.80 | 10.00 | 39.67 | 35.08 | 77.00 | 45.56 | 84.00 | 27.33 | 3.07 | 71 | 1 | 4 | 2 |
| 17w-58 | 0.00 | 0.00 | 0.00 | 0.00 | 0.00 | 0.00 | 0.00 | 0.00 | 0.00 | 0 | 2 | 3 | 2 |
| 17w-61 | 21.50 | 4.33 | 67.00 | 77.00 | 58.33 | 44.00 | 92.33 | 23.67 | 3.90 | 115 | 1 | 3 | 2 |
| 17w-62 | 29.37 | 9.00 | 56.33 | 53.17 | 81.67 | 65.10 | 85.00 | 21.00 | 4.05 | 144 | 1 | 3 | 2 |
| 17w-64 | 0.00 | 0.00 | 0.00 | 0.00 | 0.00 | 0.00 | 0.00 | 0.00 | 0.00 | 0 | 2 | 4 | 2 |
| 17w-66 | 24.23 | 12.33 | 111.67 | 19.50 | 95.33 | 20.45 | 81.00 | 25.33 | 3.20 | 161 | 1 | 3 | 2 |
| 17w-67 | 24.30 | 14.33 | 59.00 | 7.20 | 91.67 | 7.85 | 86.33 | 29.00 | 2.98 | 118 | 1 | 4 | 1 |
| 17w-68 | 22.40 | 4.00 | 80.43 | 0.23 | 135.67 | 0.17 | 82.00 | 34.33 | 2.39 | 104 | 1 | 4 | 1 |
| 17w-71 | 15.03 | 0.00 | 59.00 | 18.23 | 20.67 | 88.21 | 85.33 | 29.00 | 2.94 | 113 | 1 | 1 | 2 |

"0" represents missing data because some individuals did not flower; 1 and 2 in Flowering column represent absence of reproductive parts and appearance of reproductive parts, respectively; 1-4 in Growth habit column represent erect, semi-erect, inclining, and creeping growth habit, respectively, 1 and 2 in Leaf color column represent dark green and light green leaves, respectively.
